# Supplementary material for: LIN37-DREAM prevents DNA end resection and homologous recombination at DNA double-strand breaks in quiescent cells
Source: eLife. 2021 Sep 3;10:e68466. doi: 10.7554/eLife.68466 (PMC8416021; doi:10.7554/eLife.68466)

Figure 2A

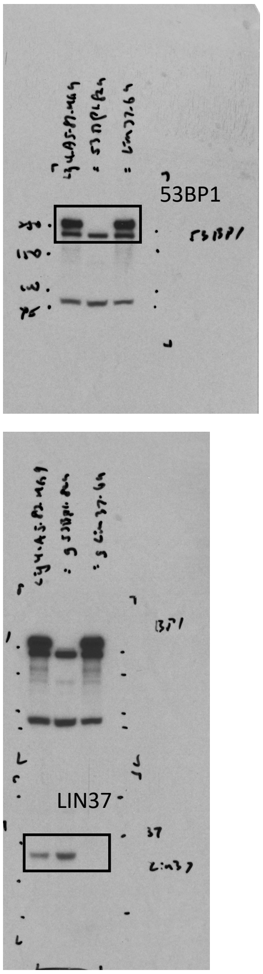

Figure 2C

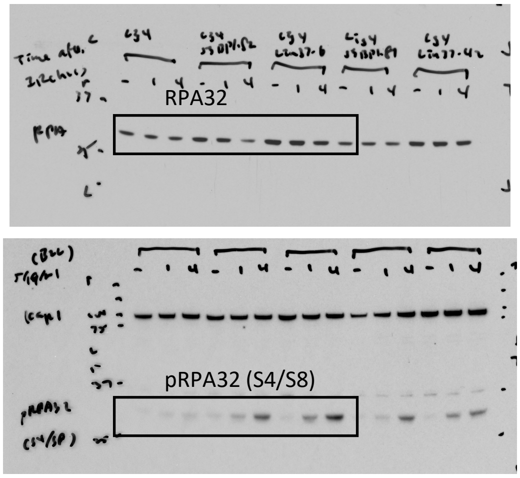

Figure 2F

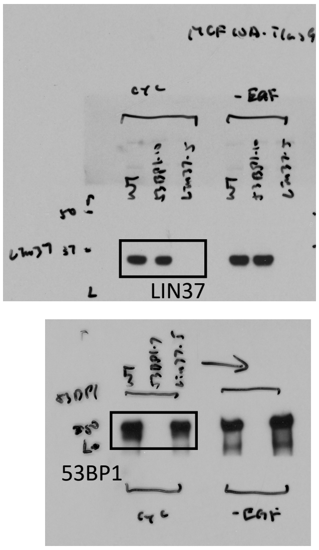

Figure 4A

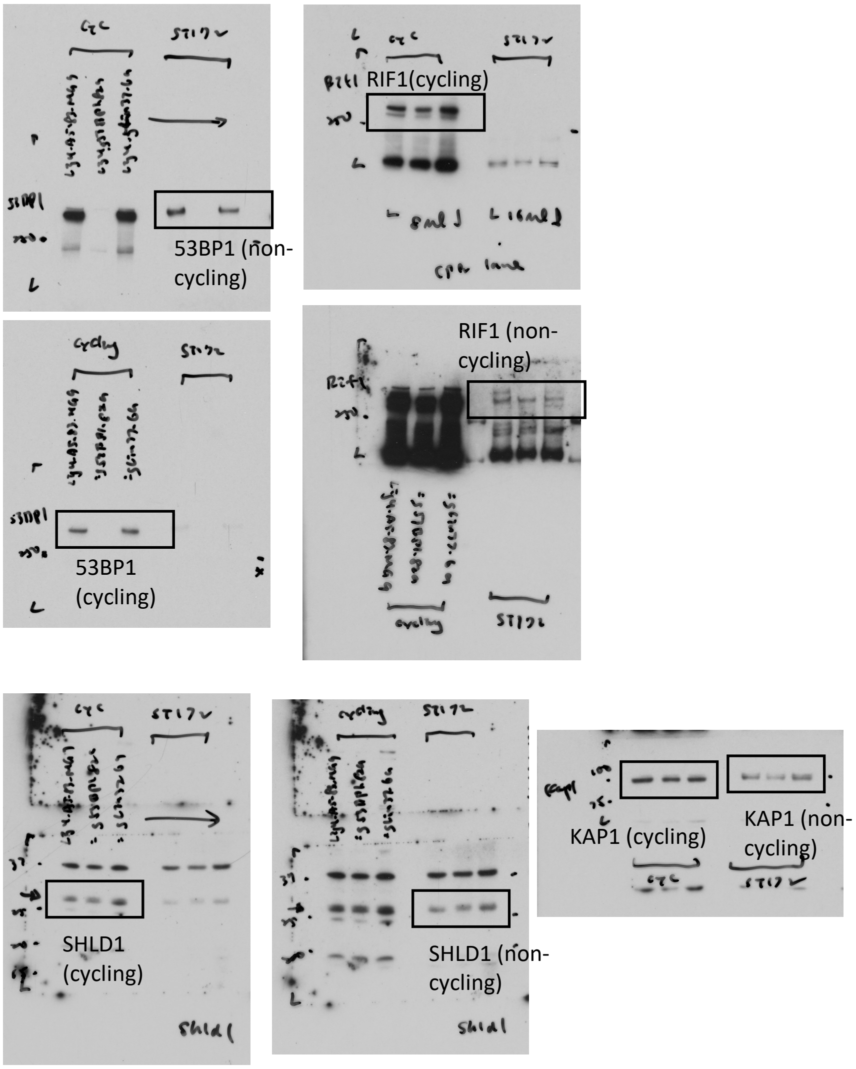

Figure 3A

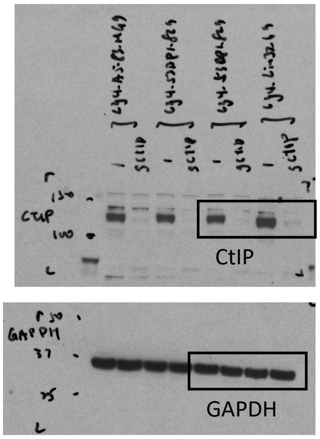

Figure 4D

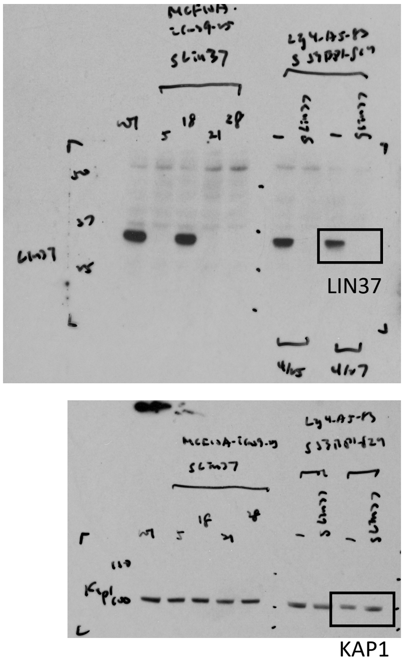

Figure 4E

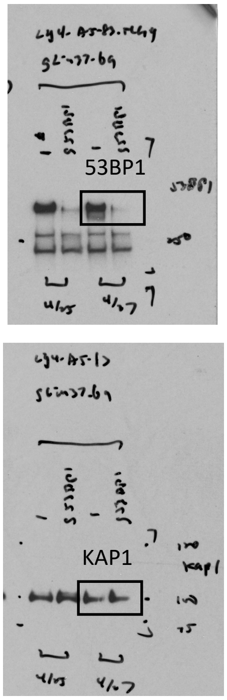

Figure 5A

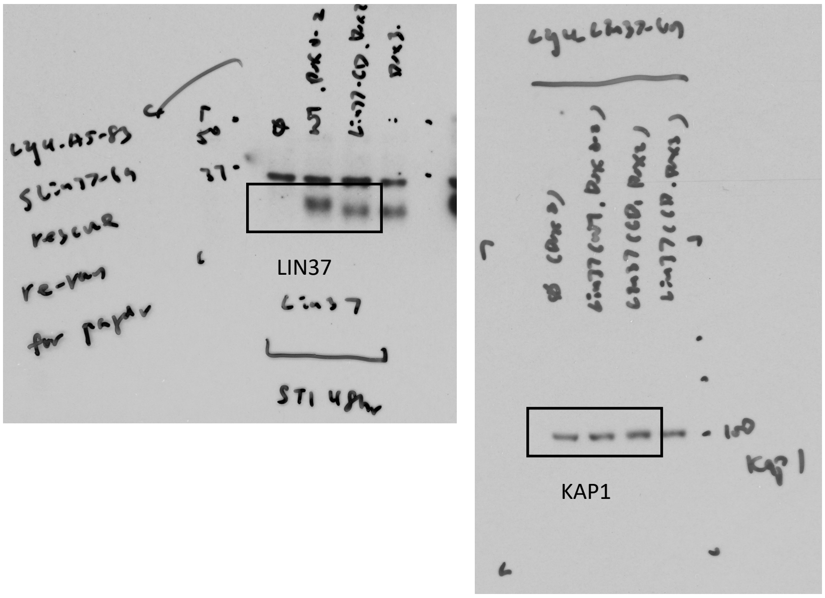

Figure 5C

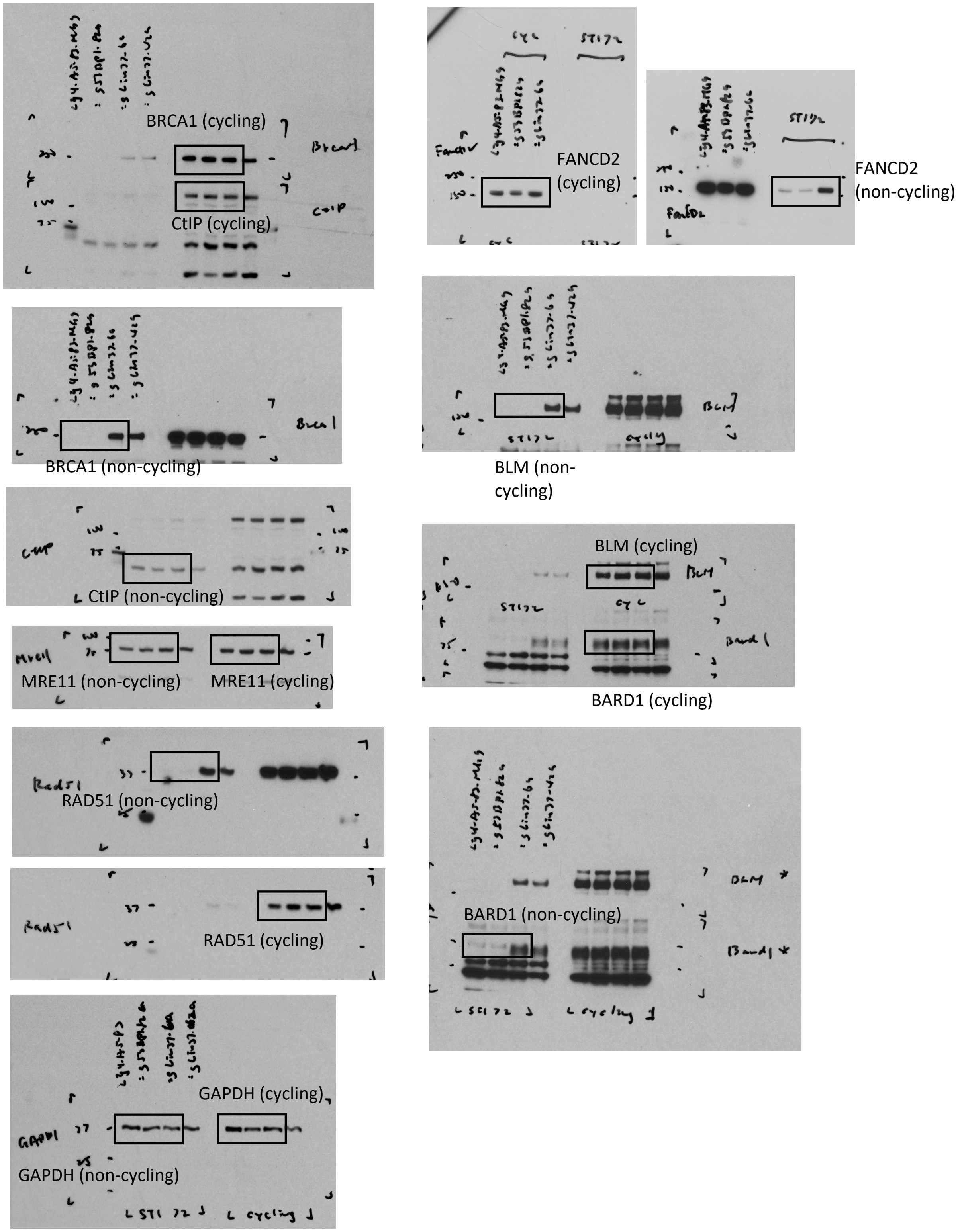
BRCA1 (cycling)
CtIP (cycling)
FANCD2 (cycling)
FANCD2 (non-cycling)
BRCA1 (non-cycling)
BLM (non-cycling)
CtIP (non-cycling)
BLM (cycling)
MRE11 (non-cycling)
MRE11 (cycling)
BARD1 (cycling)
RAD51 (non-cycling)
RAD51 (cycling)
BARD1 (non-cycling)
GAPDH (cycling)
GAPDH (non-cycling)

Figure 5D

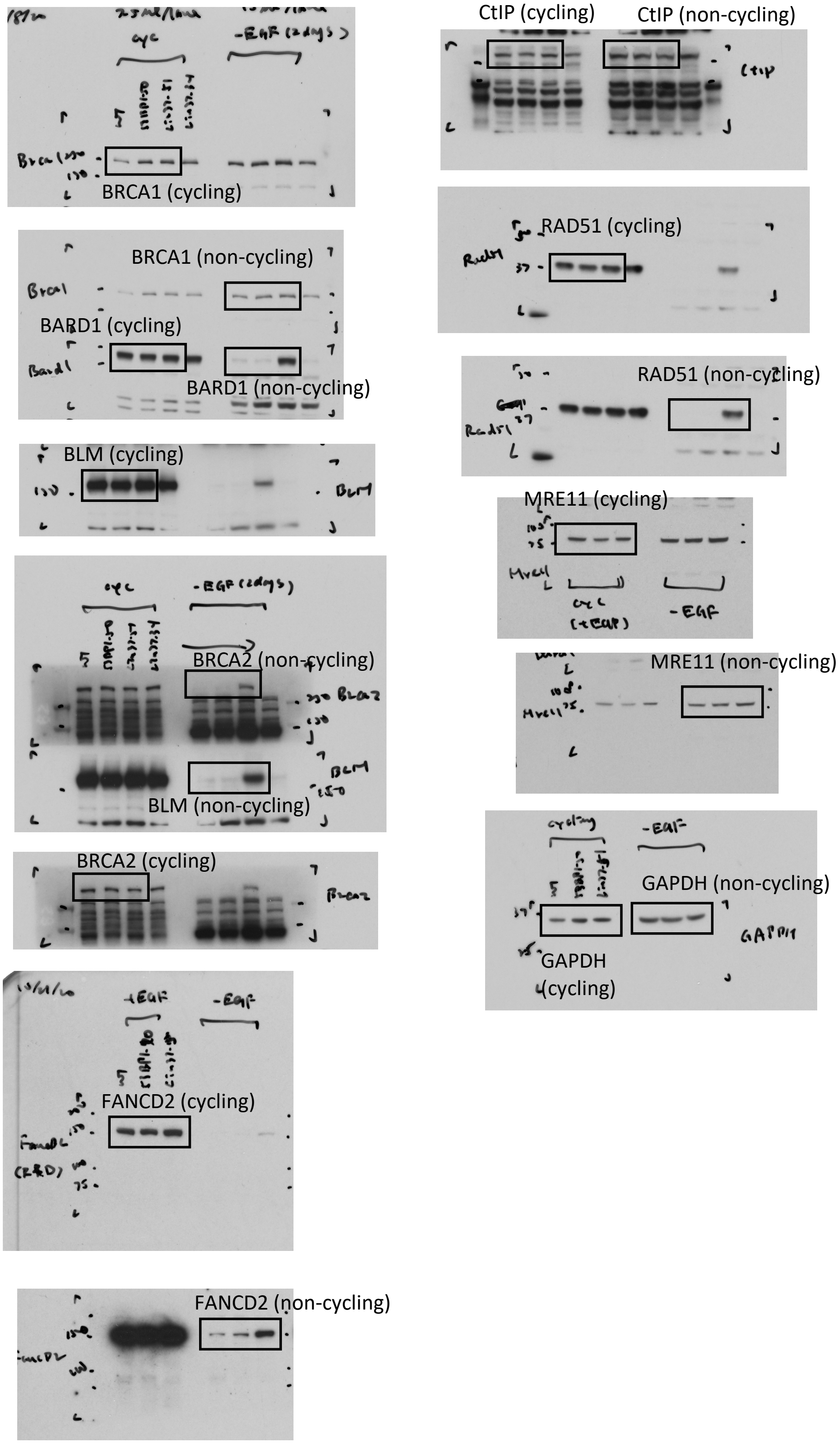

BRCA1 (cycling)
BRCA1 (non-cycling)
BARD1 (cycling)
BARD1 (non-cycling)
BLM (cycling)
BRCA2 (non-cycling)
BLM (non-cycling)
BRCA2 (cycling)
FANCD2 (cycling)
FANCD2 (non-cycling)
CtIP (cycling)
CtIP (non-cycling)
RAD51 (cycling)
RAD51 (non-cycling)
MRE11 (cycling)
MRE11 (non-cycling)
GAPDH (non-cycling)
GAPDH (cycling)

Figure 5E

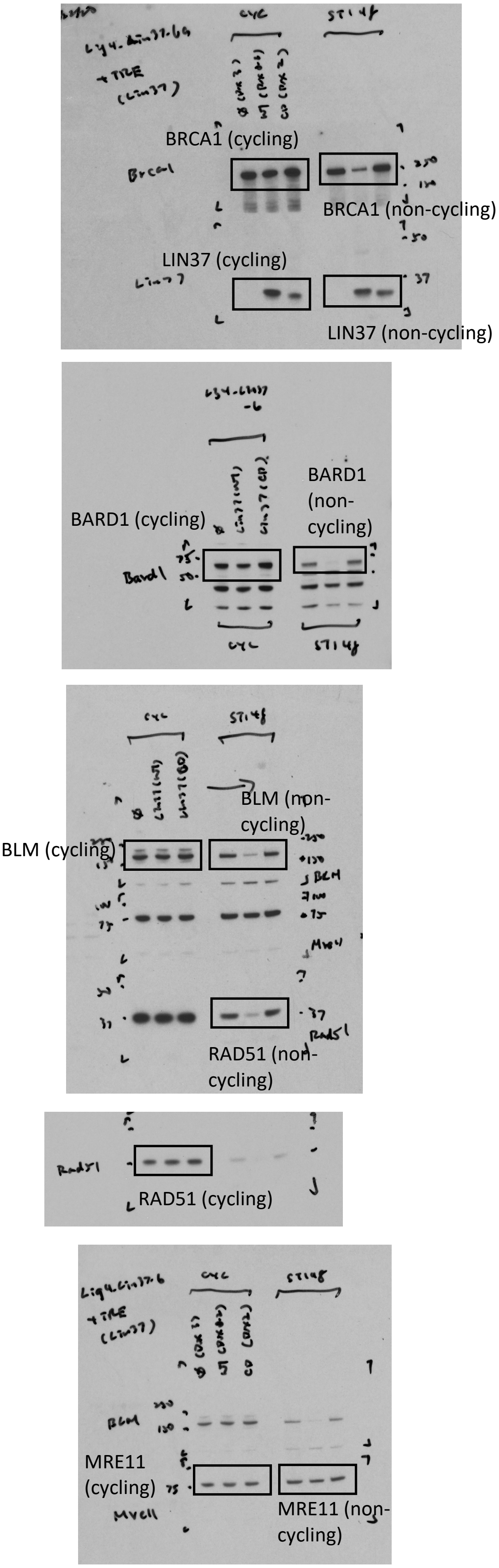

Figure 6A

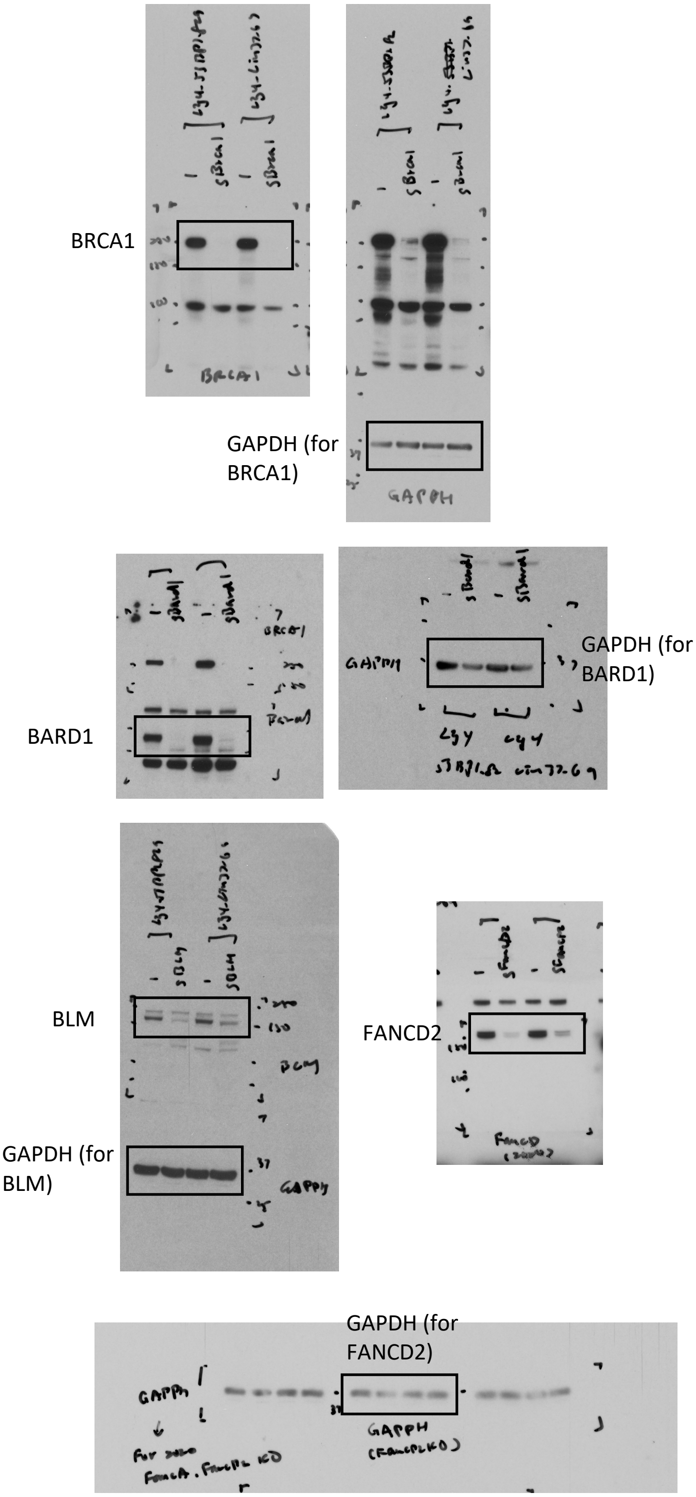

Figure 7A

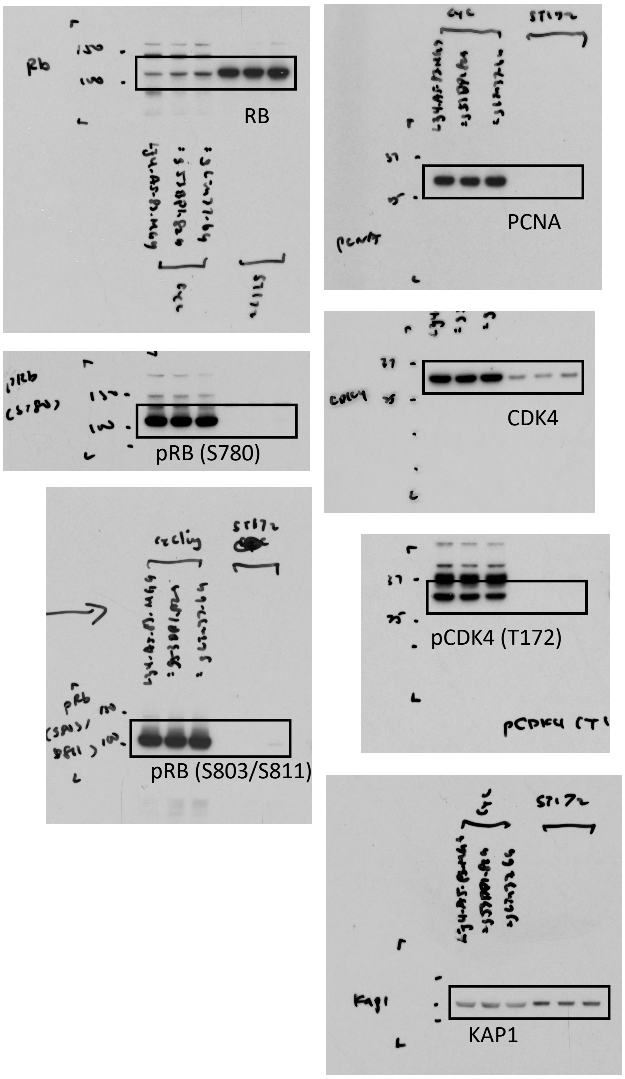

Figure 7B

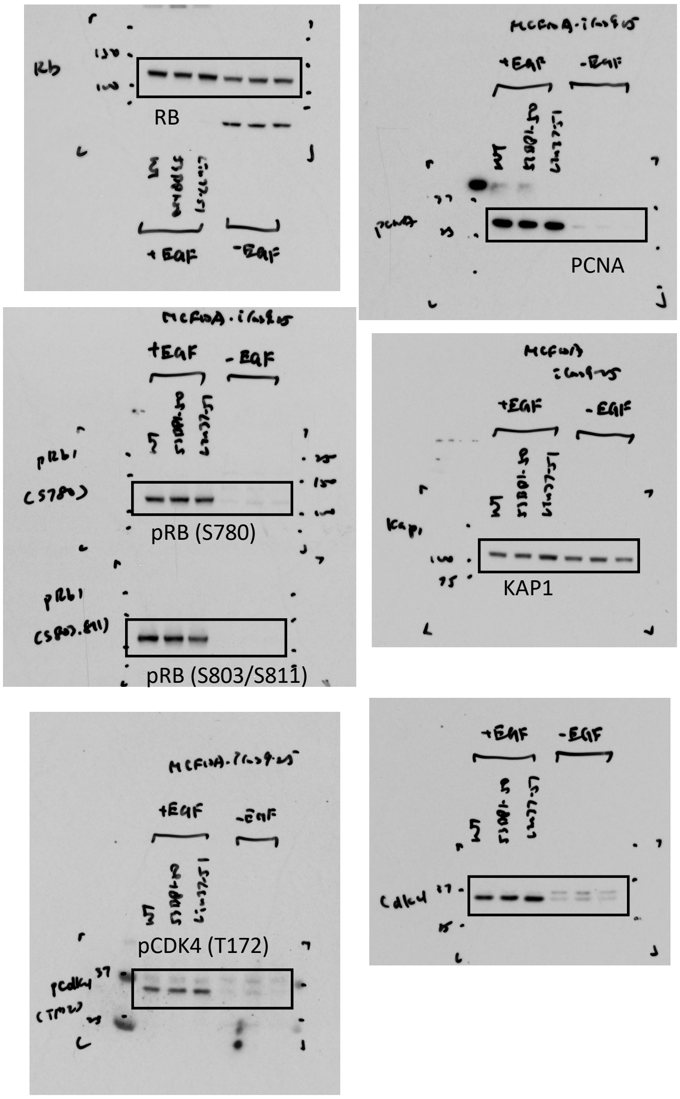

Figure 7C

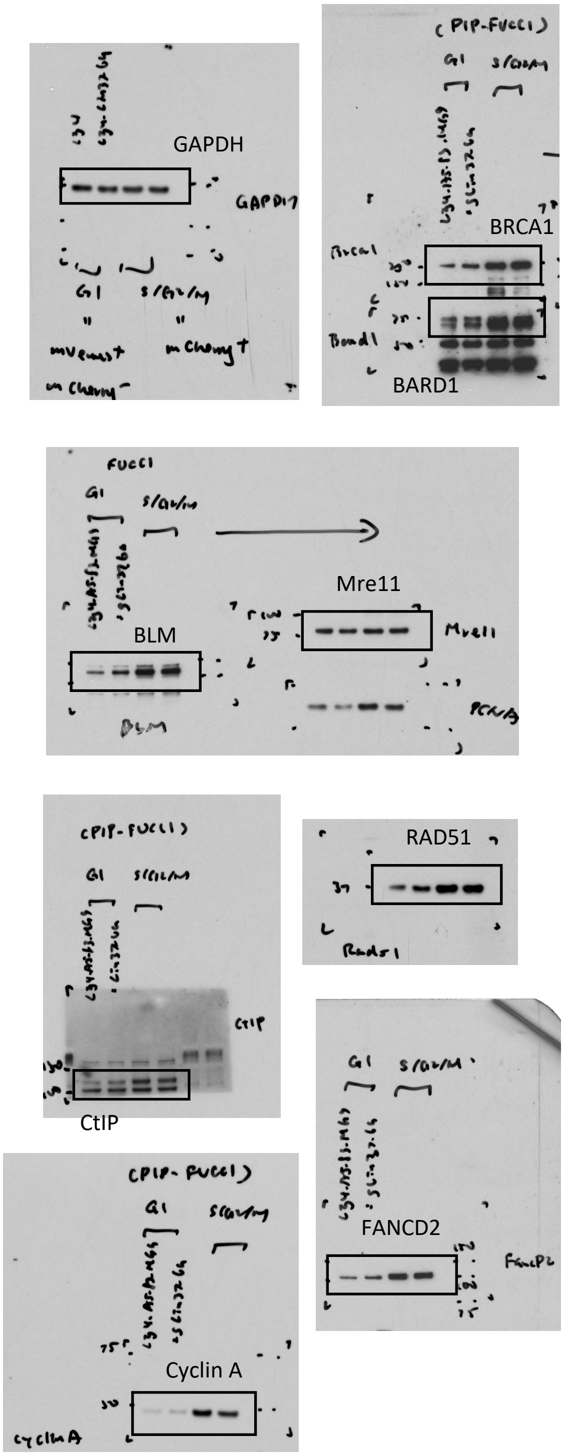

Figure 7D

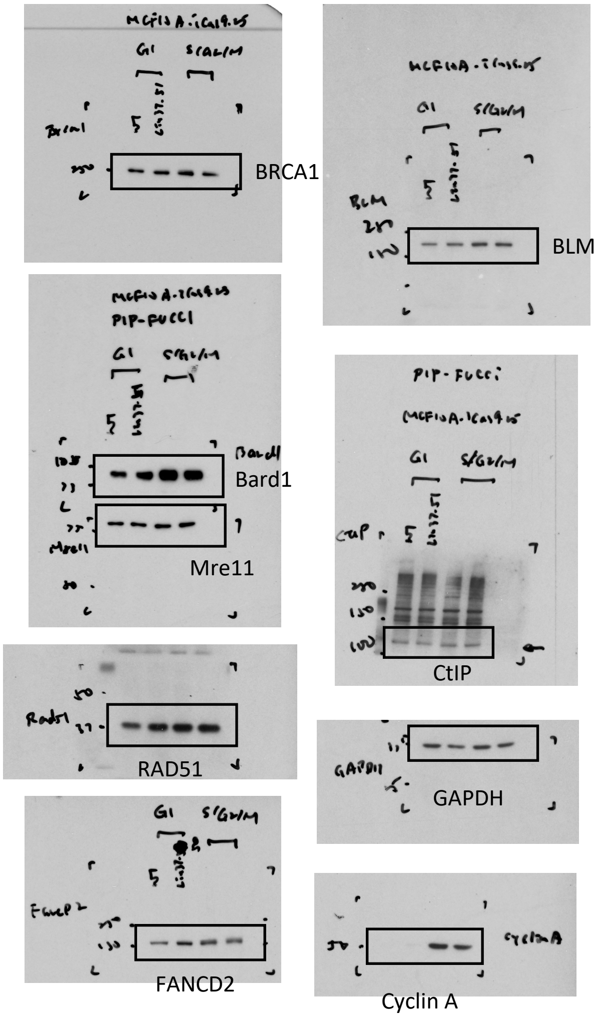

Figure 2 supplement 1A

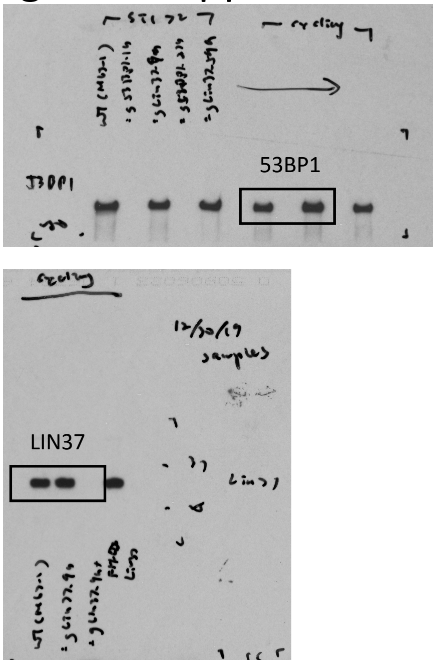

Figure 4 supplement 1A

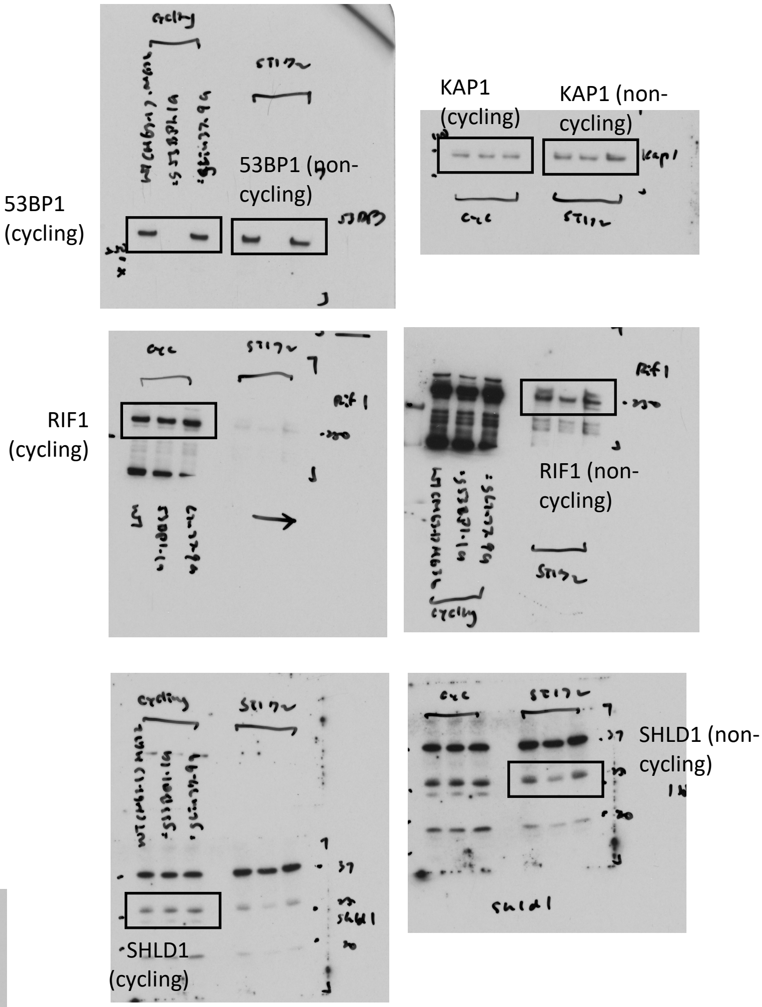

Figure 3 supplement 1B

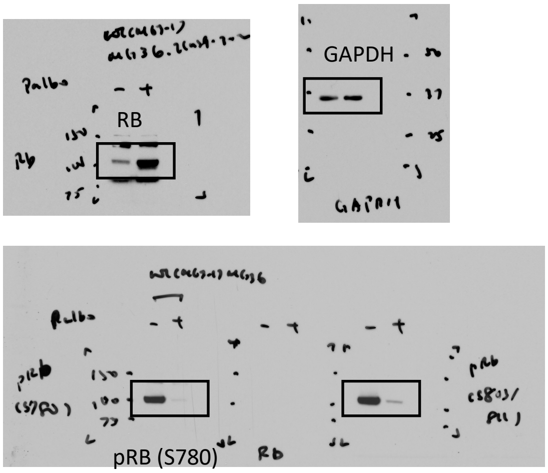

Figure 5-figure supplement 1B

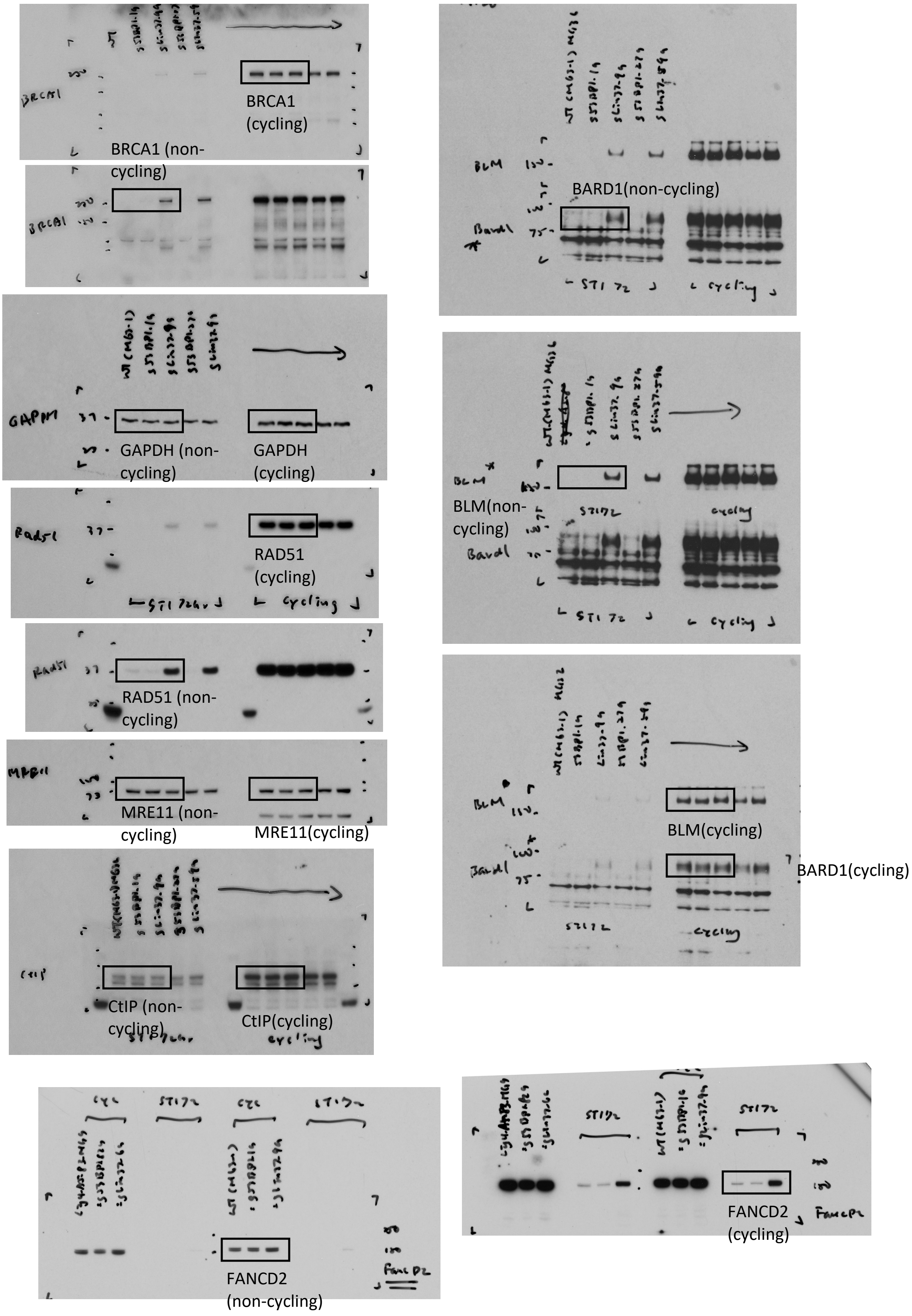

Supplement: Source data 1. [file elife-68466-data1.pdf]
