# Supplementary figures and images for: LIN37-DREAM prevents DNA end resection and homologous recombination at DNA double-strand breaks in quiescent cells (part 1 of 2)
Source: eLife. 2021 Sep 3;10:e68466. doi: 10.7554/eLife.68466 (PMC8416021; doi:10.7554/eLife.68466)

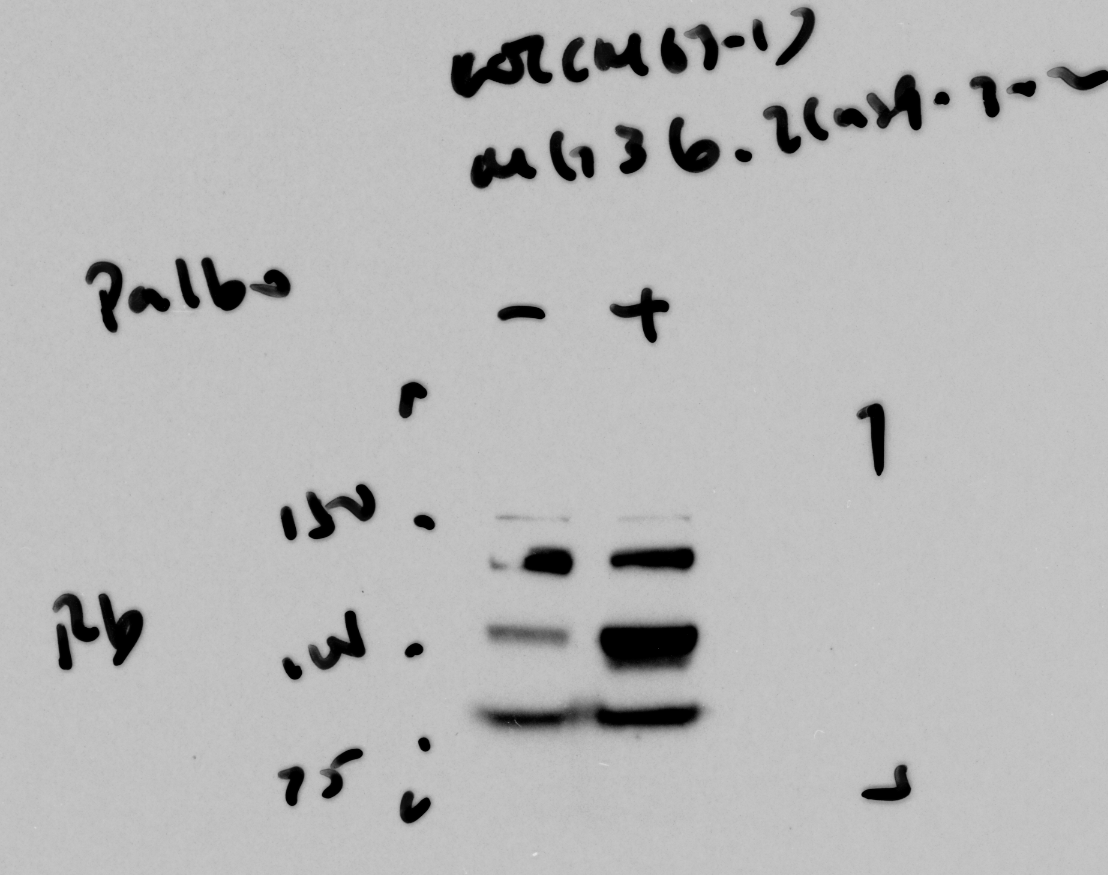

Supplement: Source data 2. [file elife-68466-data2.zip › Source data 2 - figure 2 and 3/Figure 3/0630210003_RB_Fig 3S1B.tif]

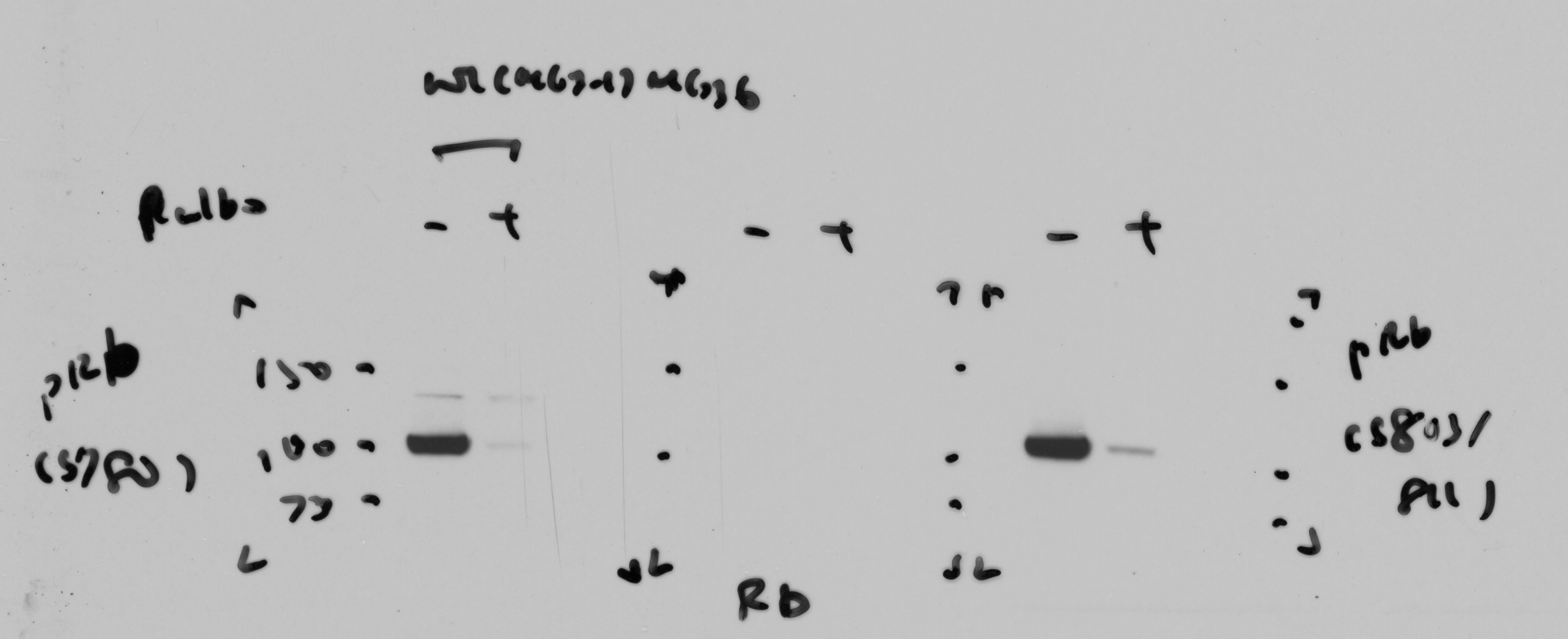

Supplement: Source data 2. [file elife-68466-data2.zip › Source data 2 - figure 2 and 3/Figure 3/063021_pRBP_Fig 3S1B.tif]

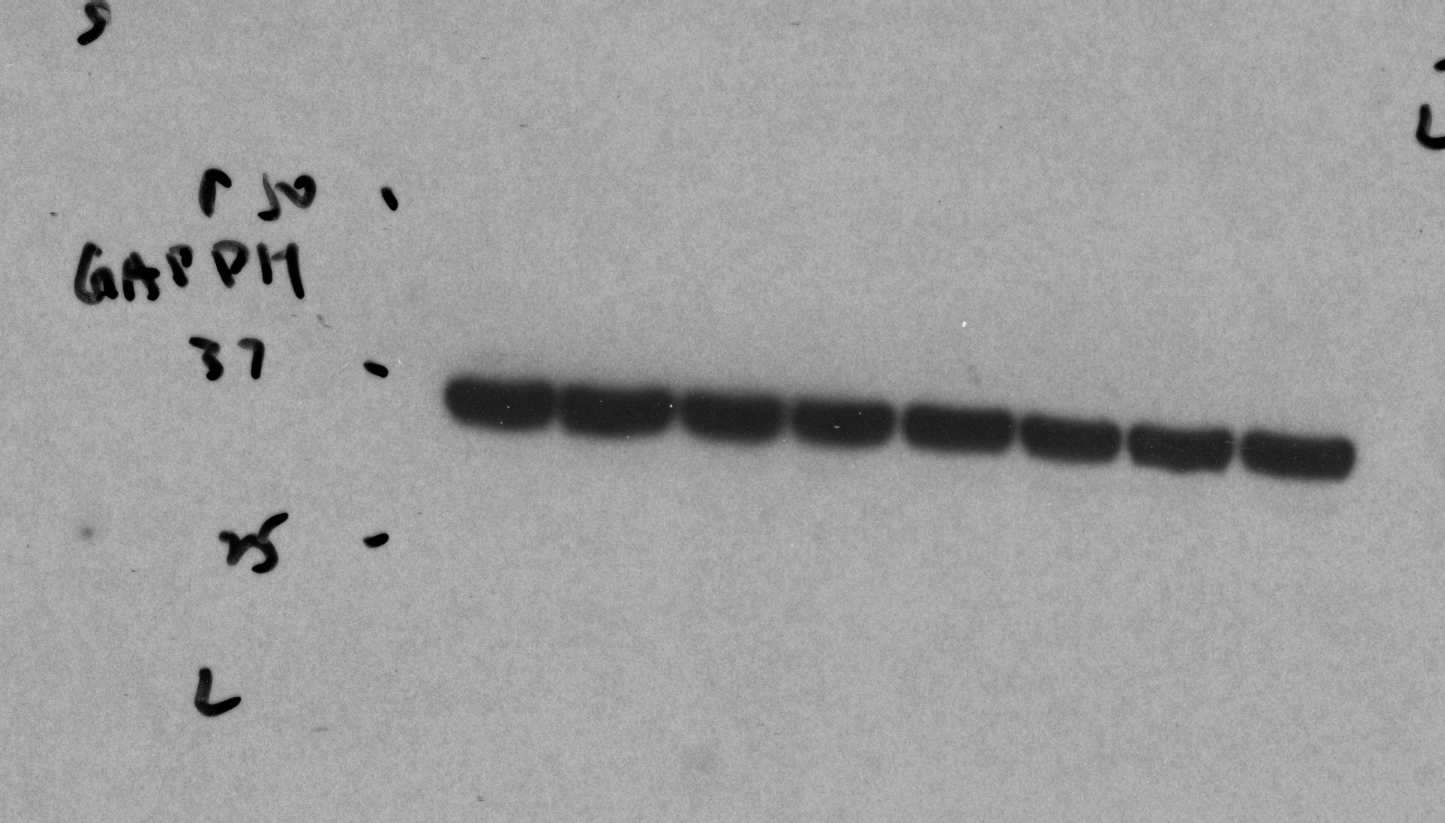

Supplement: Source data 2. [file elife-68466-data2.zip › Source data 2 - figure 2 and 3/Figure 3/050821_GAPDH_Fig 3A.tif]

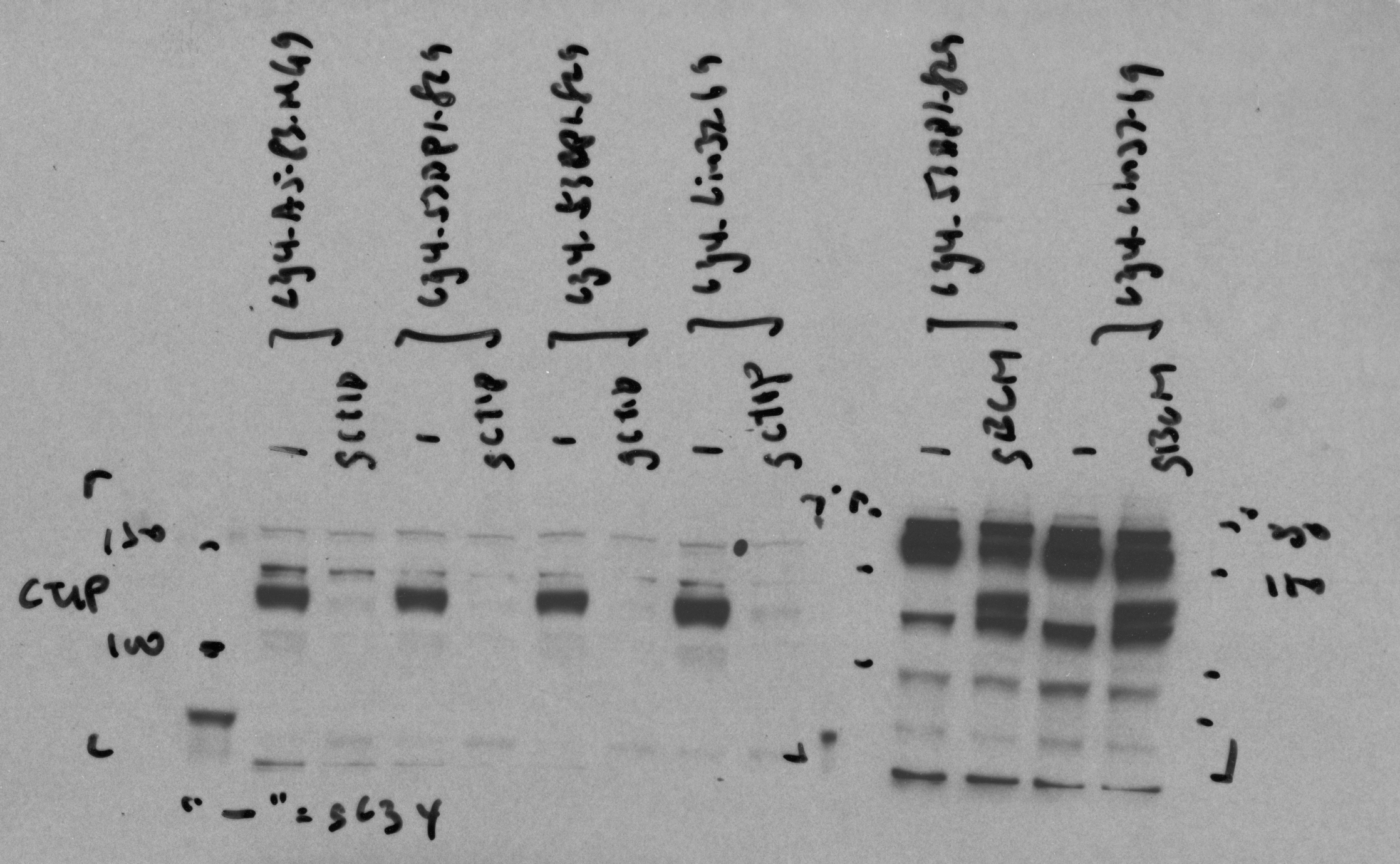

Supplement: Source data 2. [file elife-68466-data2.zip › Source data 2 - figure 2 and 3/Figure 3/050821_CtIP_Fig 3A.tif]

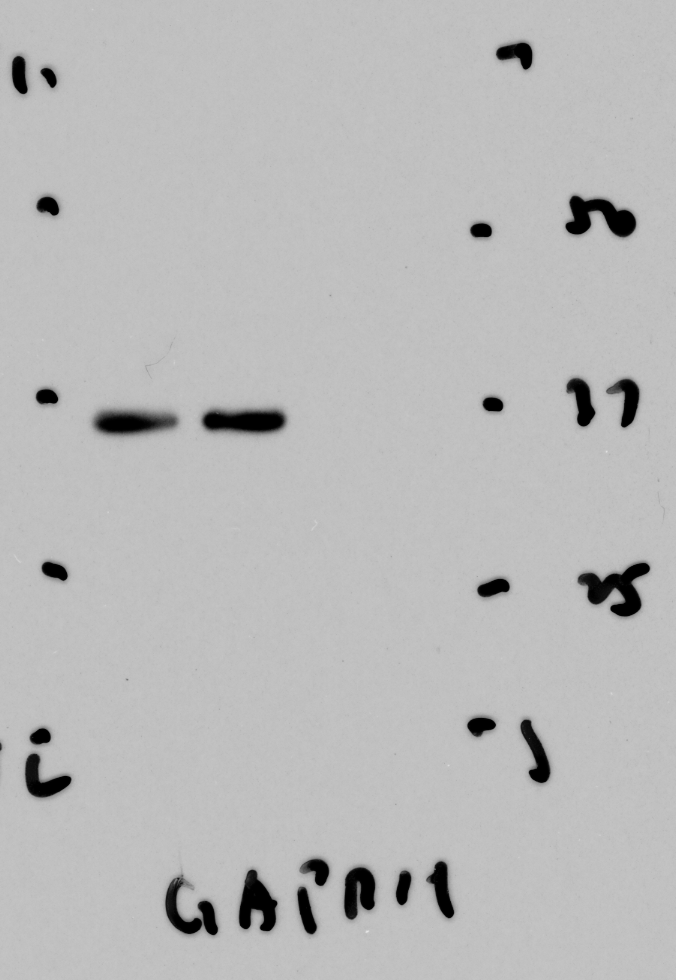

Supplement: Source data 2. [file elife-68466-data2.zip › Source data 2 - figure 2 and 3/Figure 3/0630210001_GAPDH_Fig 3S1B.tif]

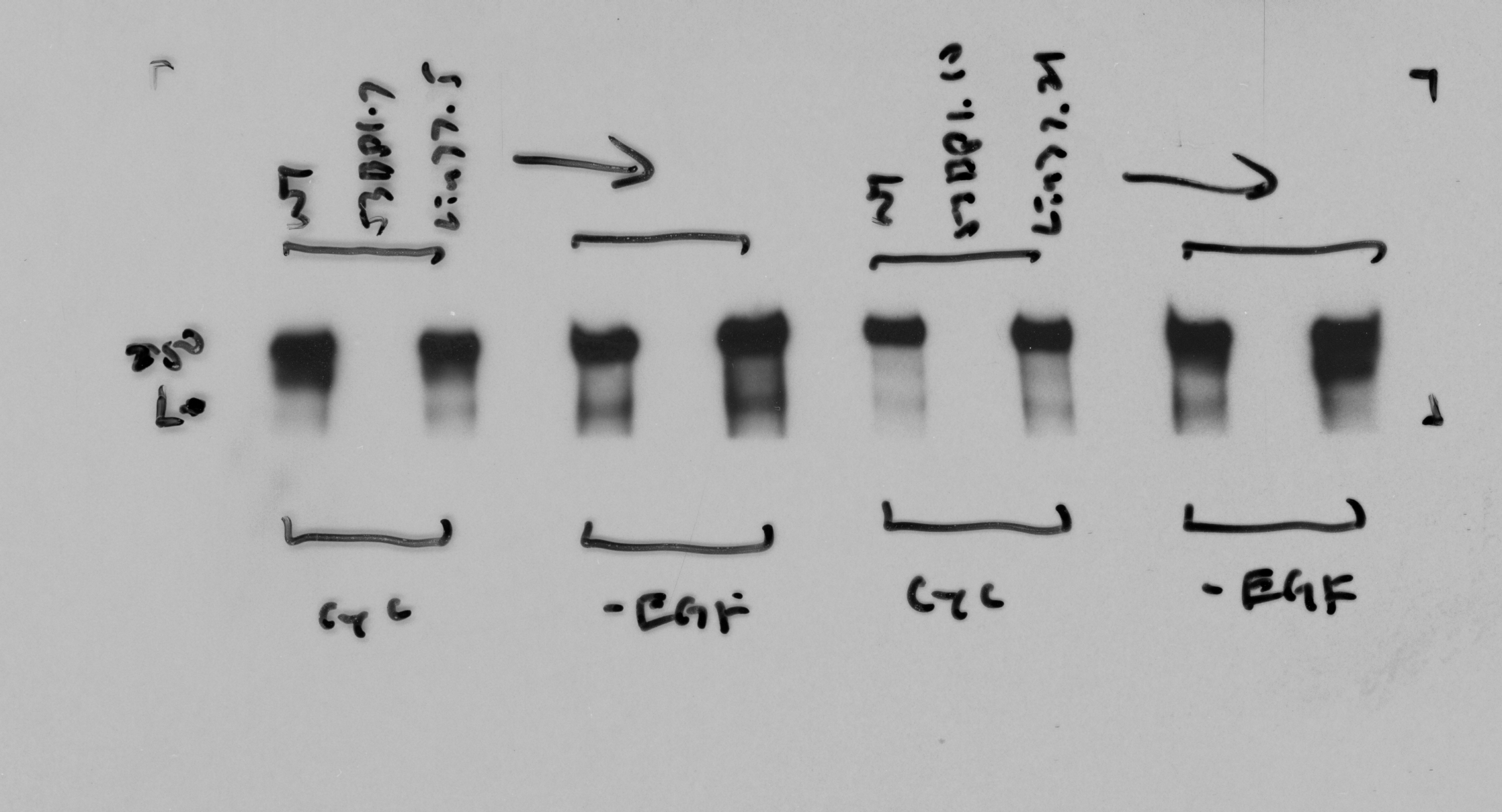

Supplement: Source data 2. [file elife-68466-data2.zip › Source data 2 - figure 2 and 3/Figure 2/051920_Fig 2F_53bp1.tif]

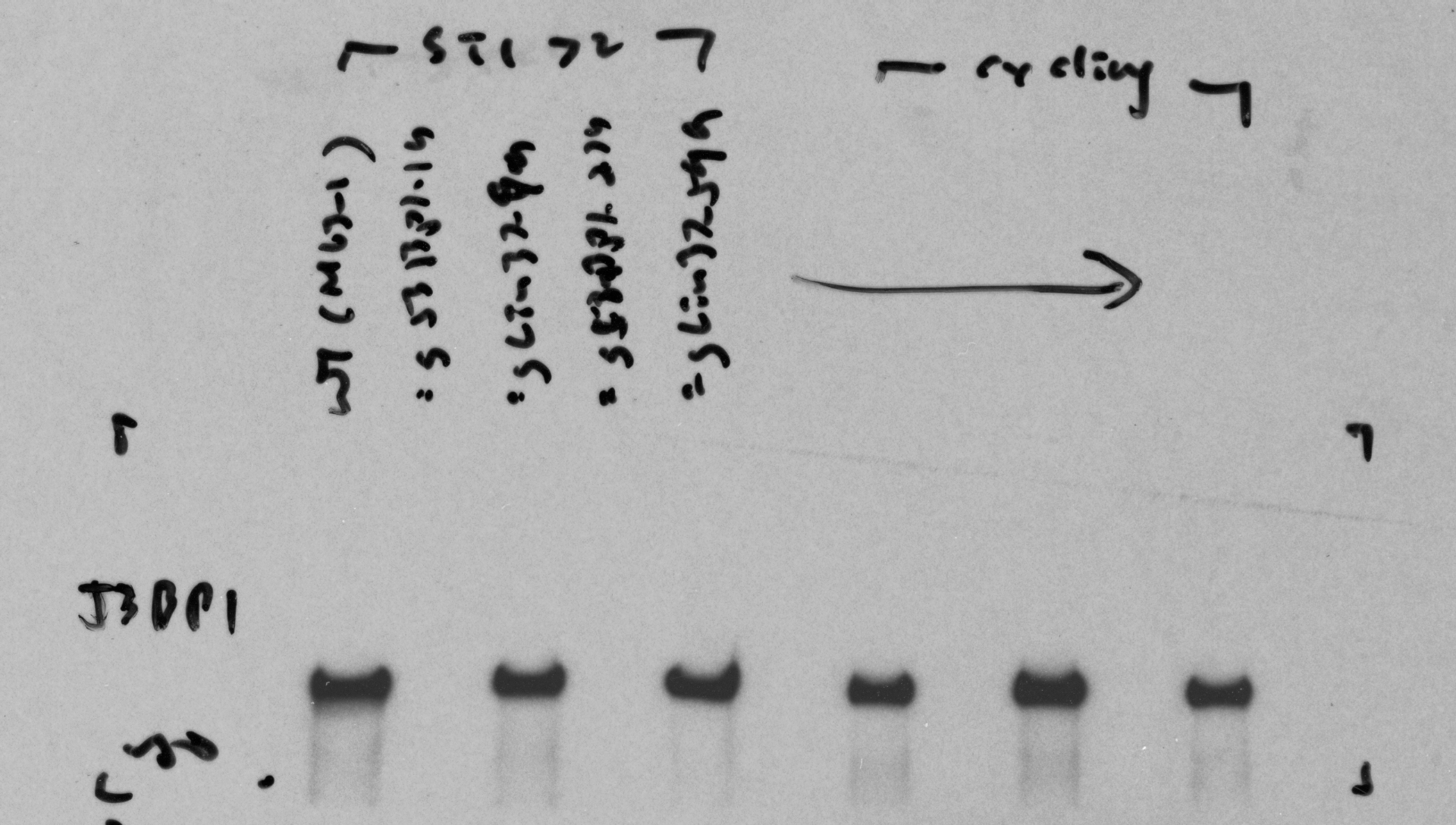

Supplement: Source data 2. [file elife-68466-data2.zip › Source data 2 - figure 2 and 3/Figure 2/032720_53bp1CtIP_Fig 2S1A.tif]

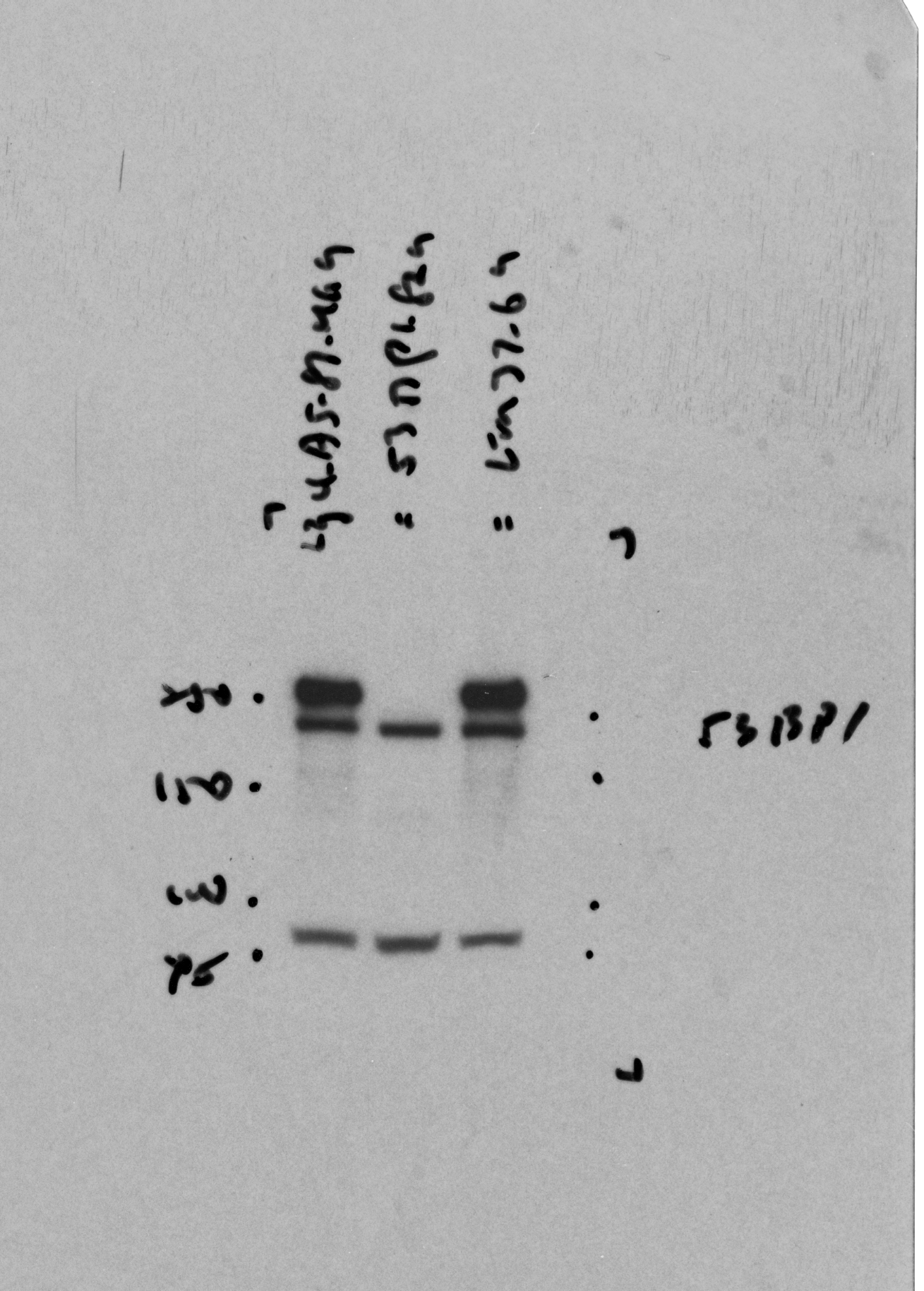

Supplement: Source data 2. [file elife-68466-data2.zip › Source data 2 - figure 2 and 3/Figure 2/031320_Fig 2A_BP1.tif]

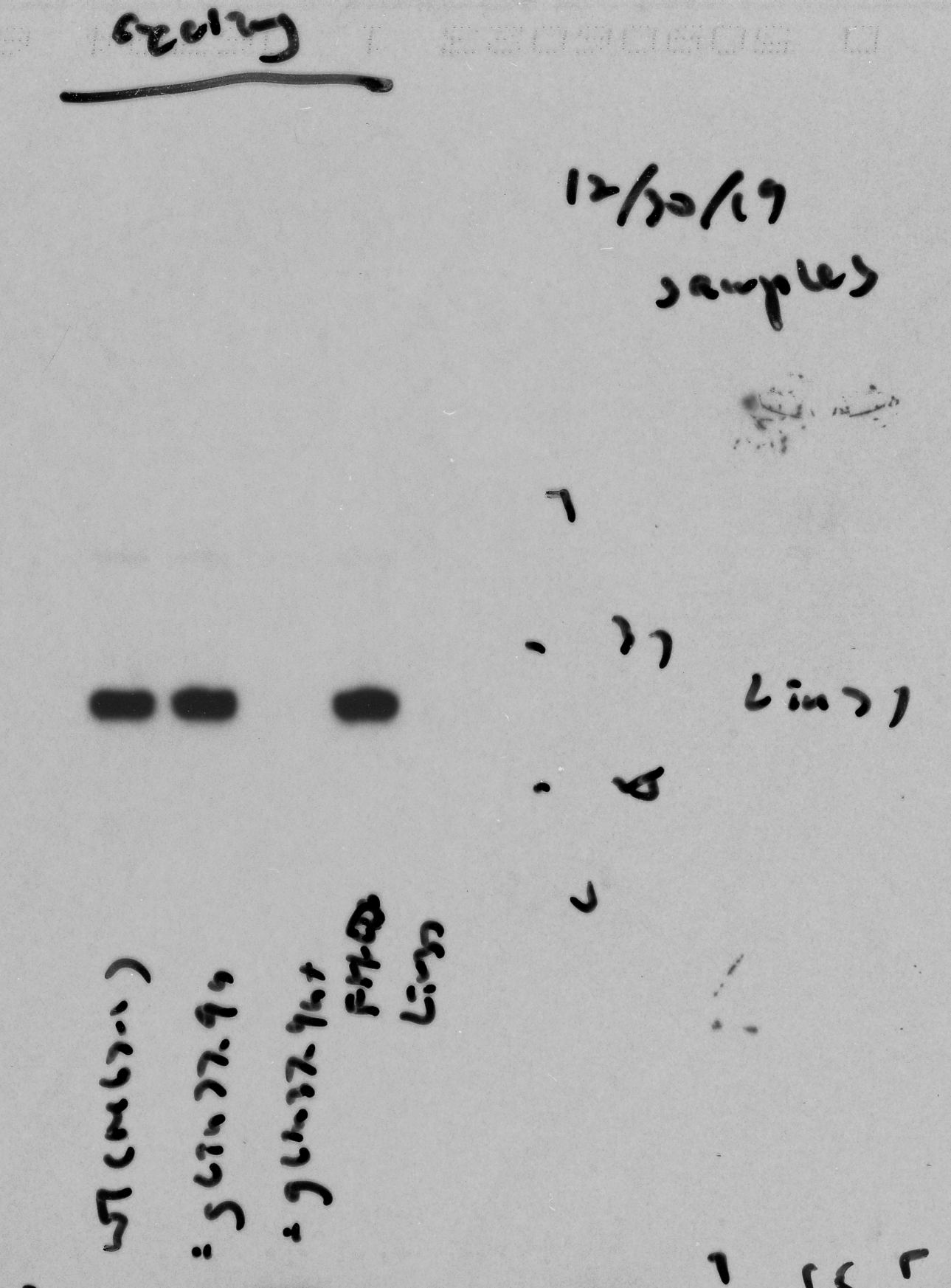

Supplement: Source data 2. [file elife-68466-data2.zip › Source data 2 - figure 2 and 3/Figure 2/040120_LIN37_Fig 2S1B_1.tif]

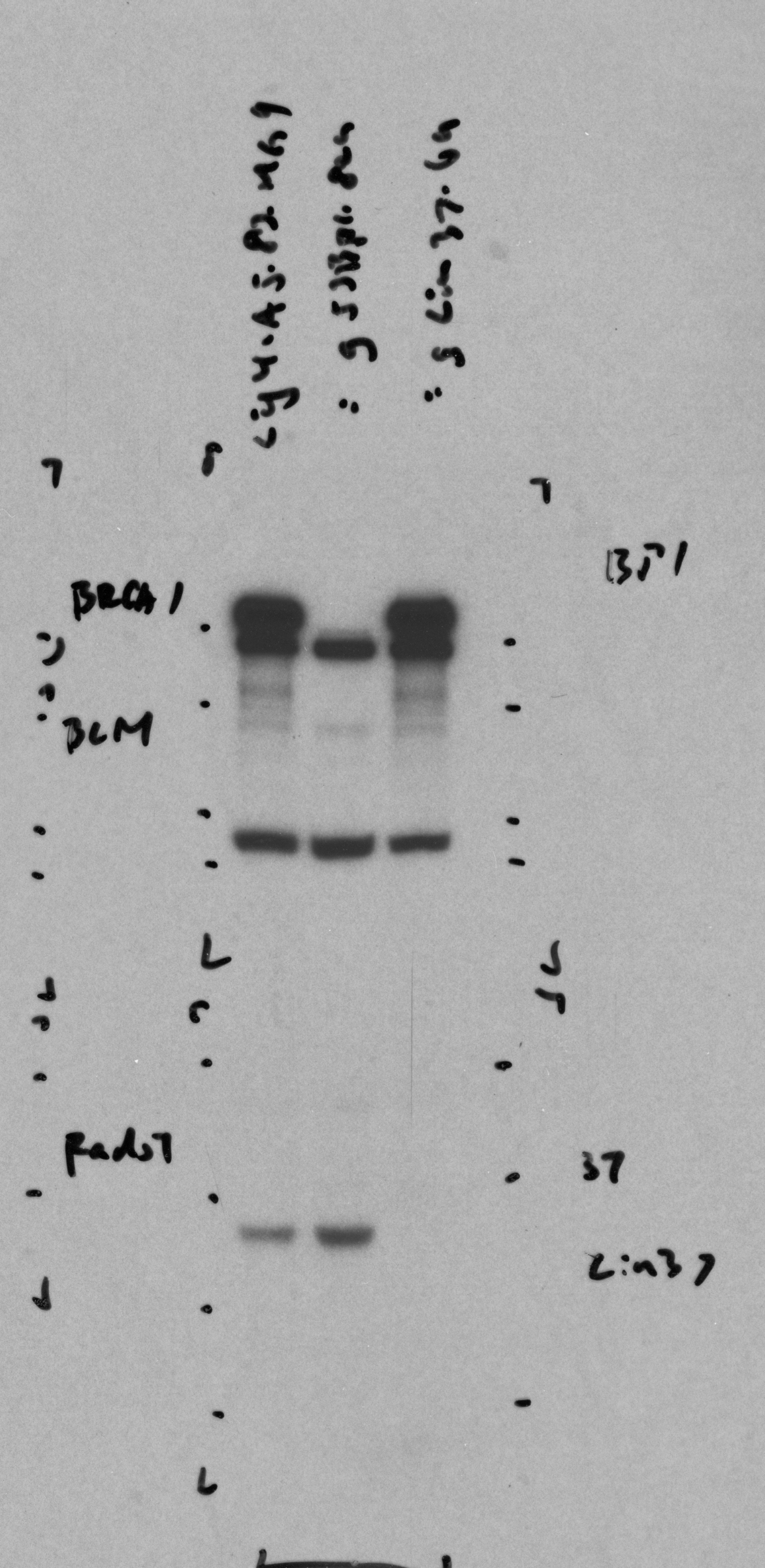

Supplement: Source data 2. [file elife-68466-data2.zip › Source data 2 - figure 2 and 3/Figure 2/031320_Fig 2A_lin37.tif]

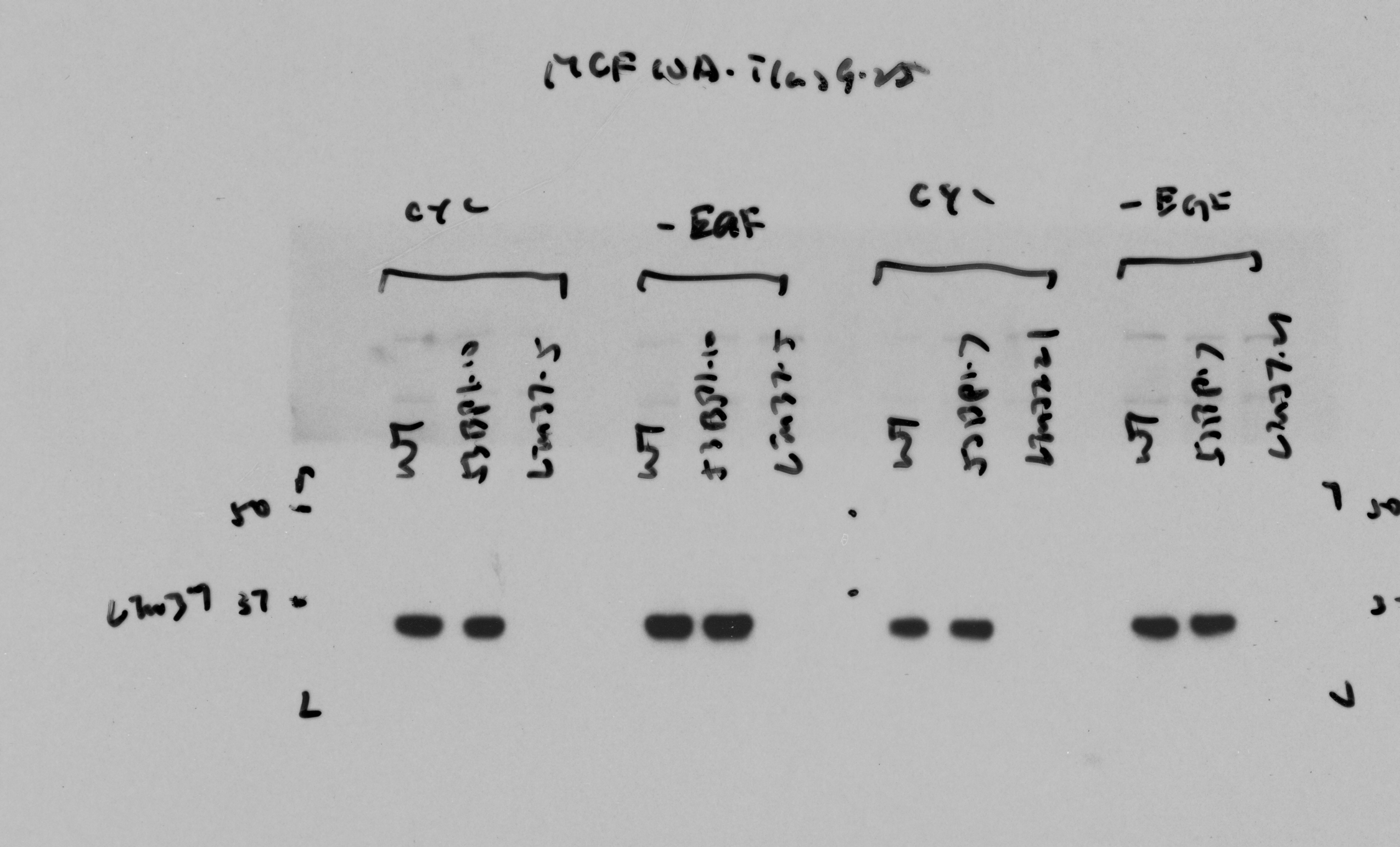

Supplement: Source data 2. [file elife-68466-data2.zip › Source data 2 - figure 2 and 3/Figure 2/051920_Fig 2F_lin37.tif]

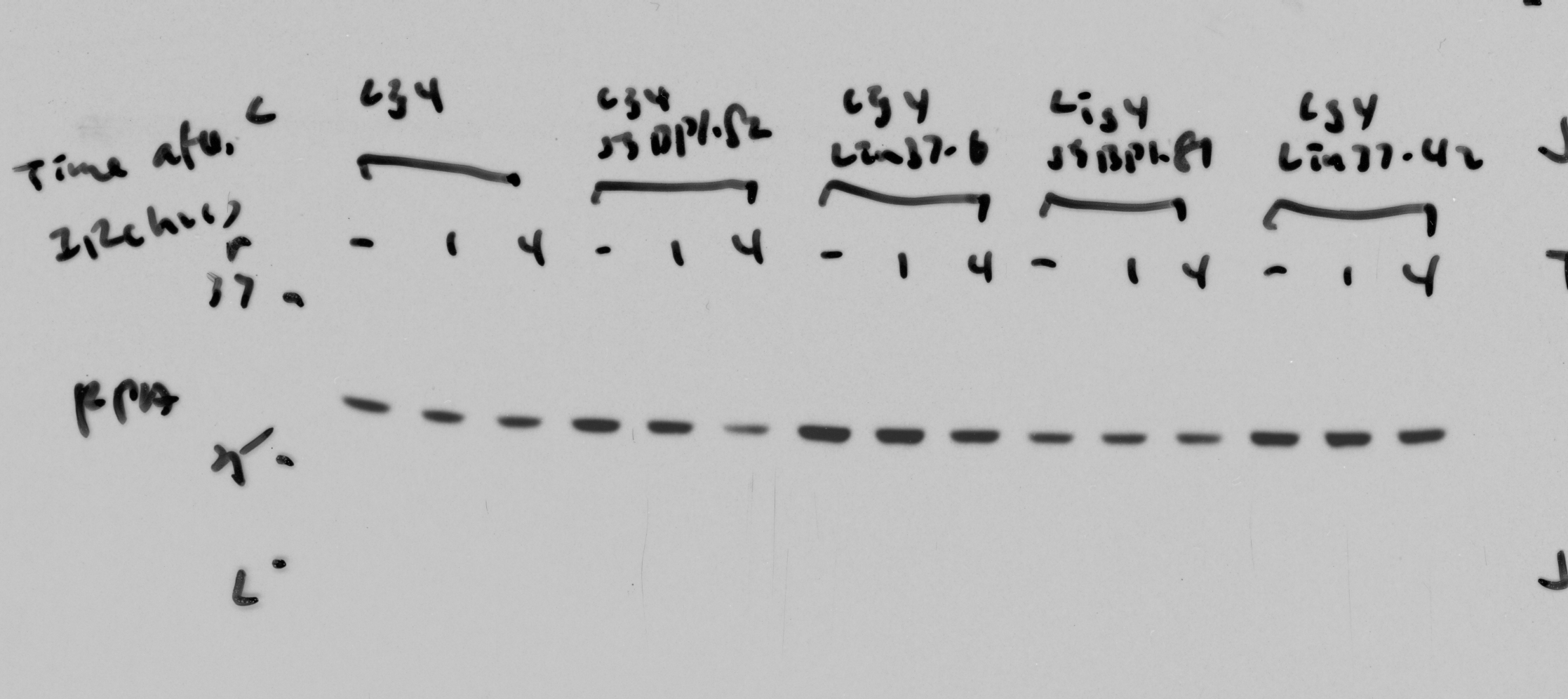

Supplement: Source data 2. [file elife-68466-data2.zip › Source data 2 - figure 2 and 3/Figure 2/060921_Fig 2C_rpa.tif]

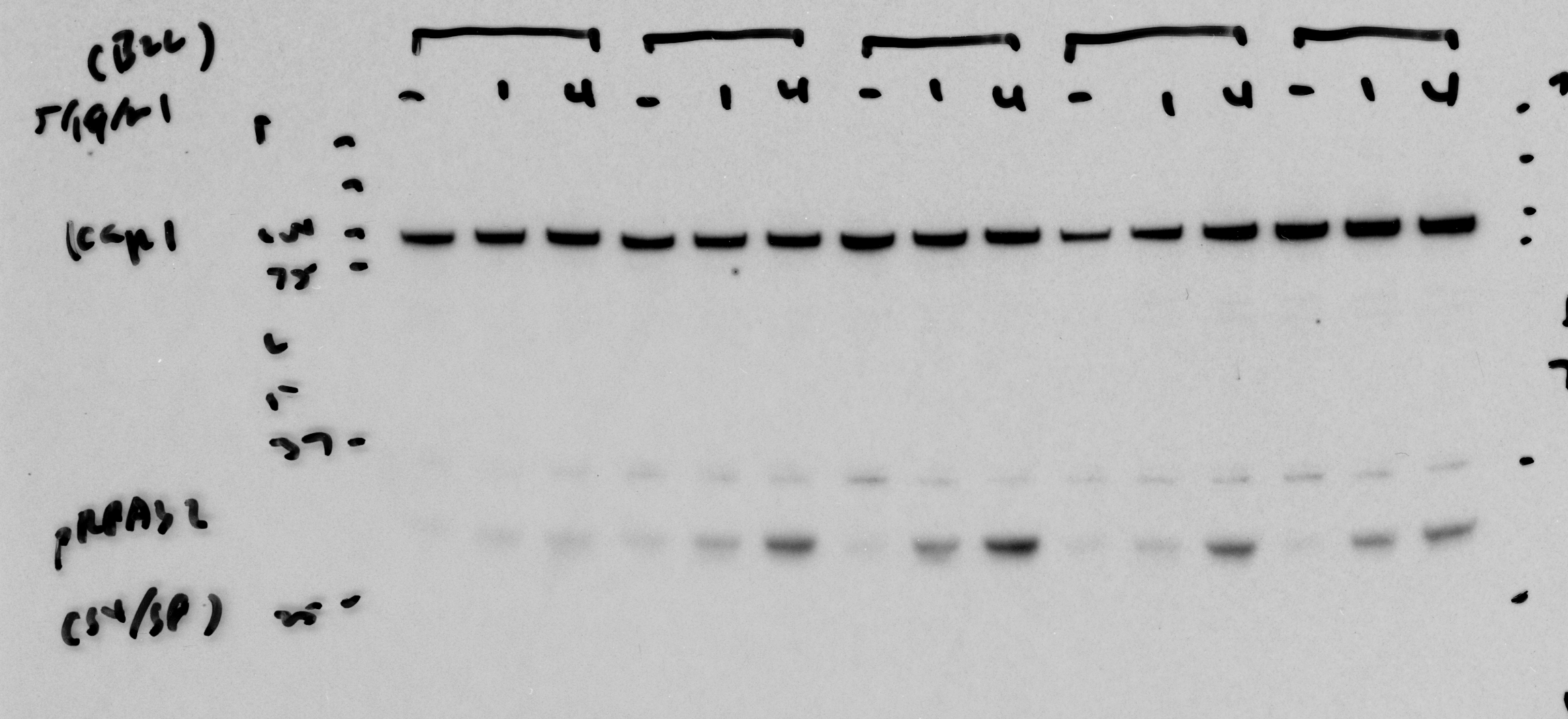

Supplement: Source data 2. [file elife-68466-data2.zip › Source data 2 - figure 2 and 3/Figure 2/061821_pRPA32_Fig 2C.tif]

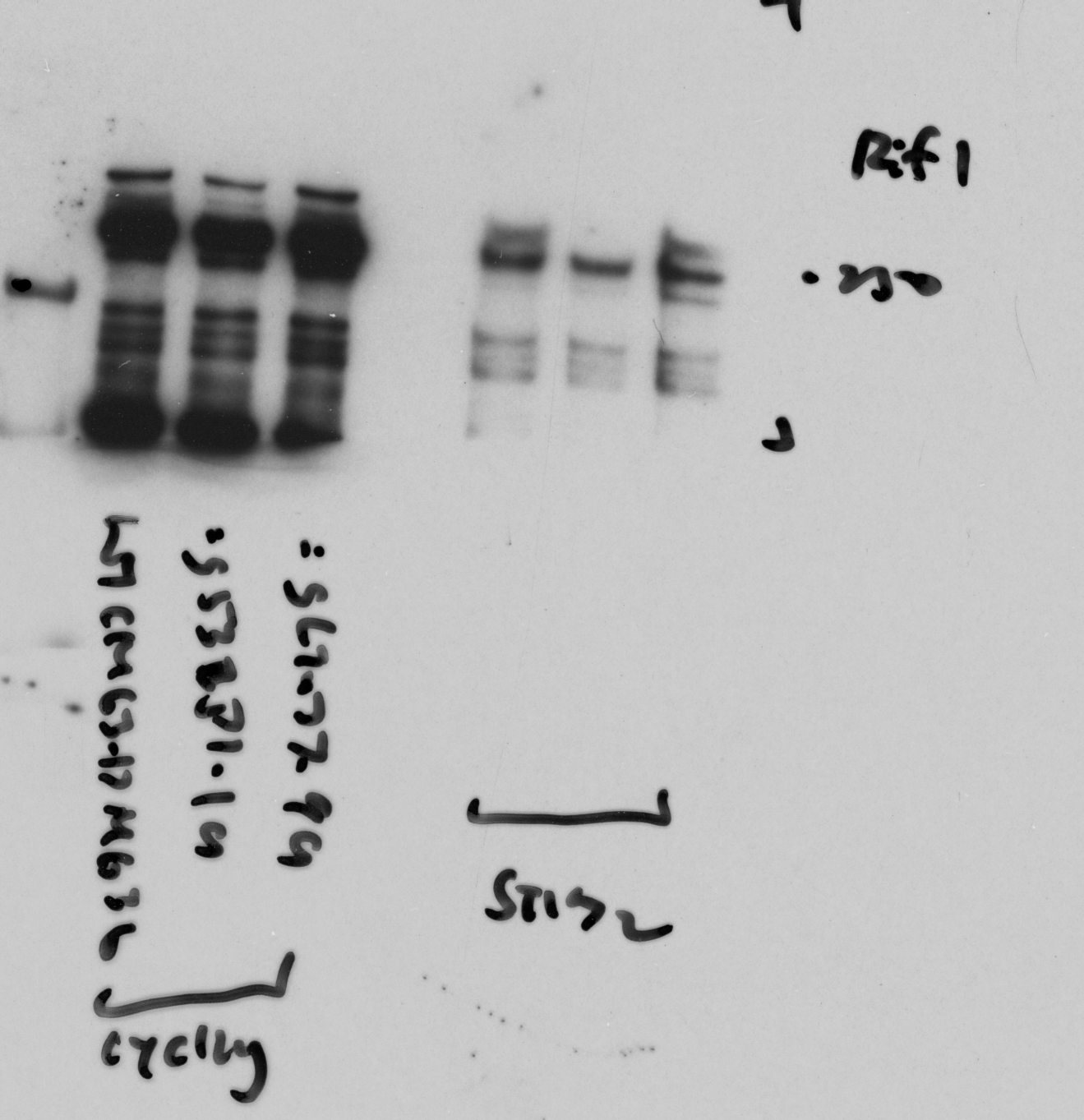

Supplement: Source data 3. [file elife-68466-data3.zip › Source data 3 - figure 4/Figure 4/083120_Rif1_Fig 4 S1A.tif]

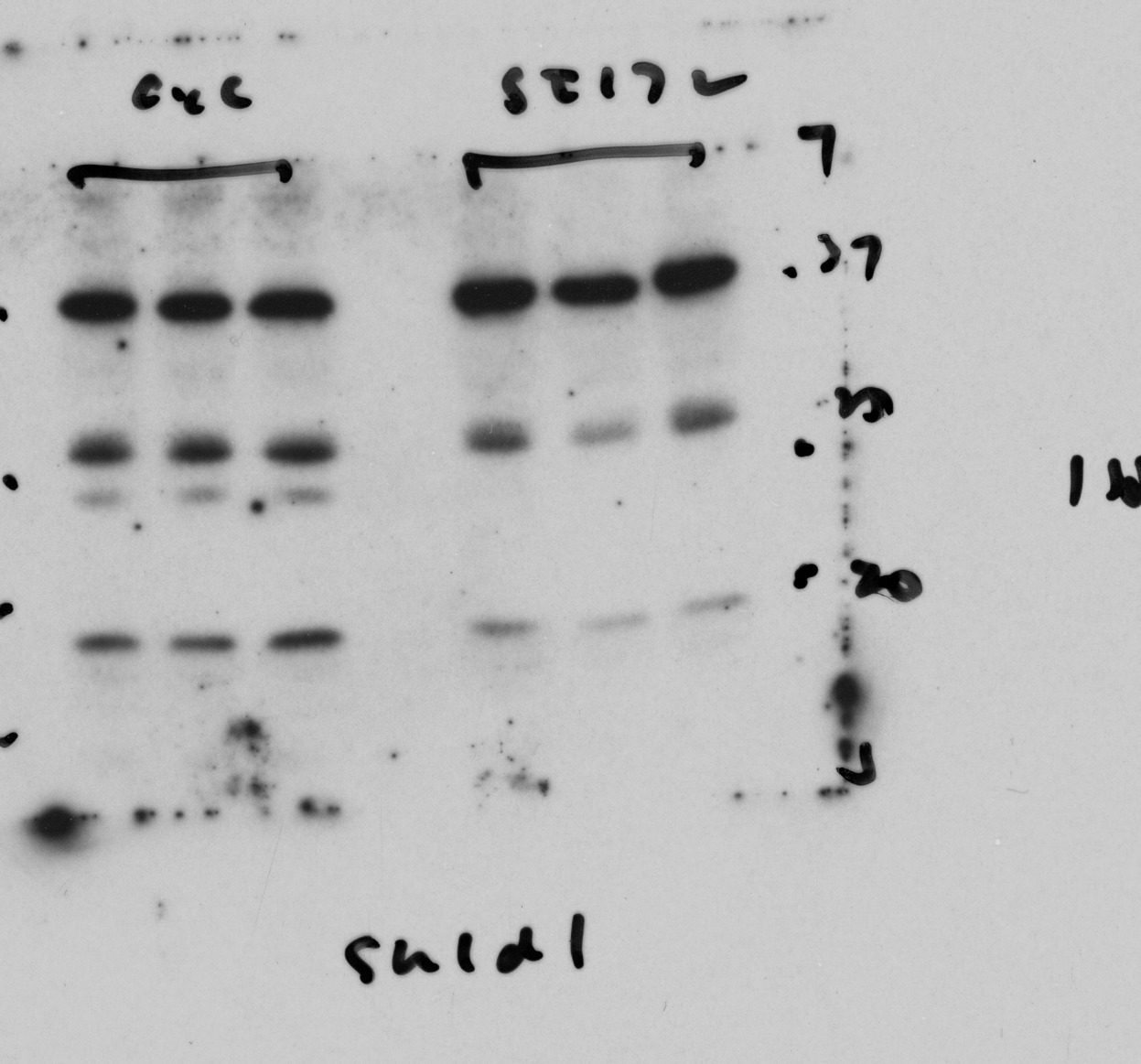

Supplement: Source data 3. [file elife-68466-data3.zip › Source data 3 - figure 4/Figure 4/083120_Shld1_Fig 4 S1A0001.tif]

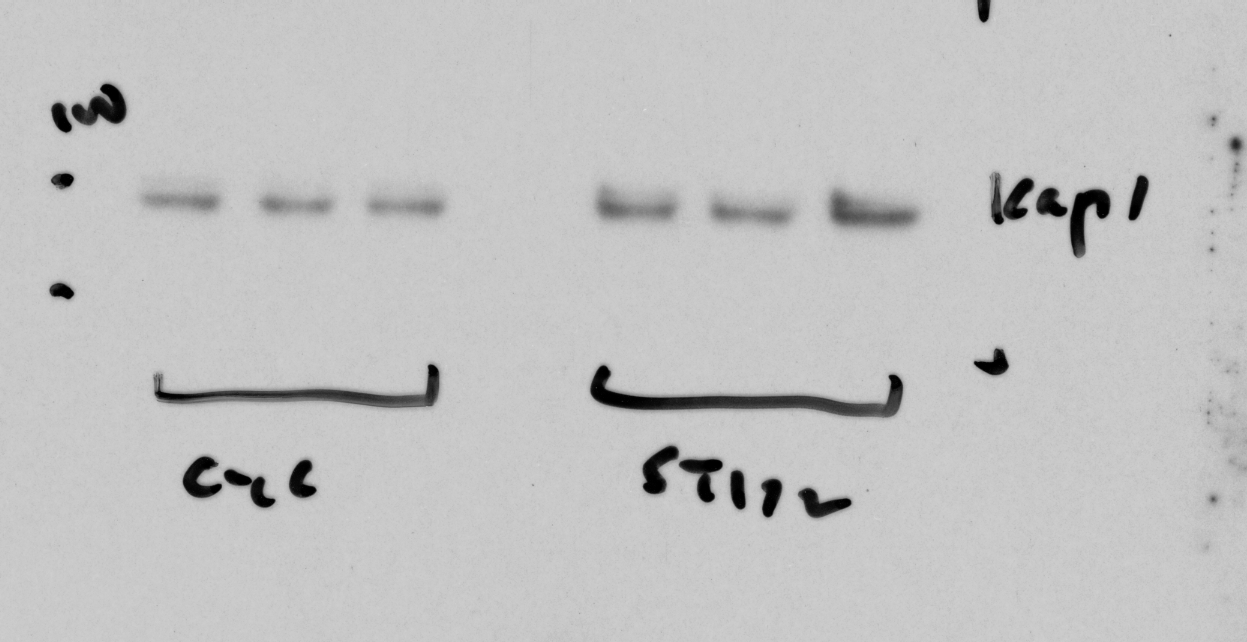

Supplement: Source data 3. [file elife-68466-data3.zip › Source data 3 - figure 4/Figure 4/083120_KAP1_Fig 4 S1A.tif]

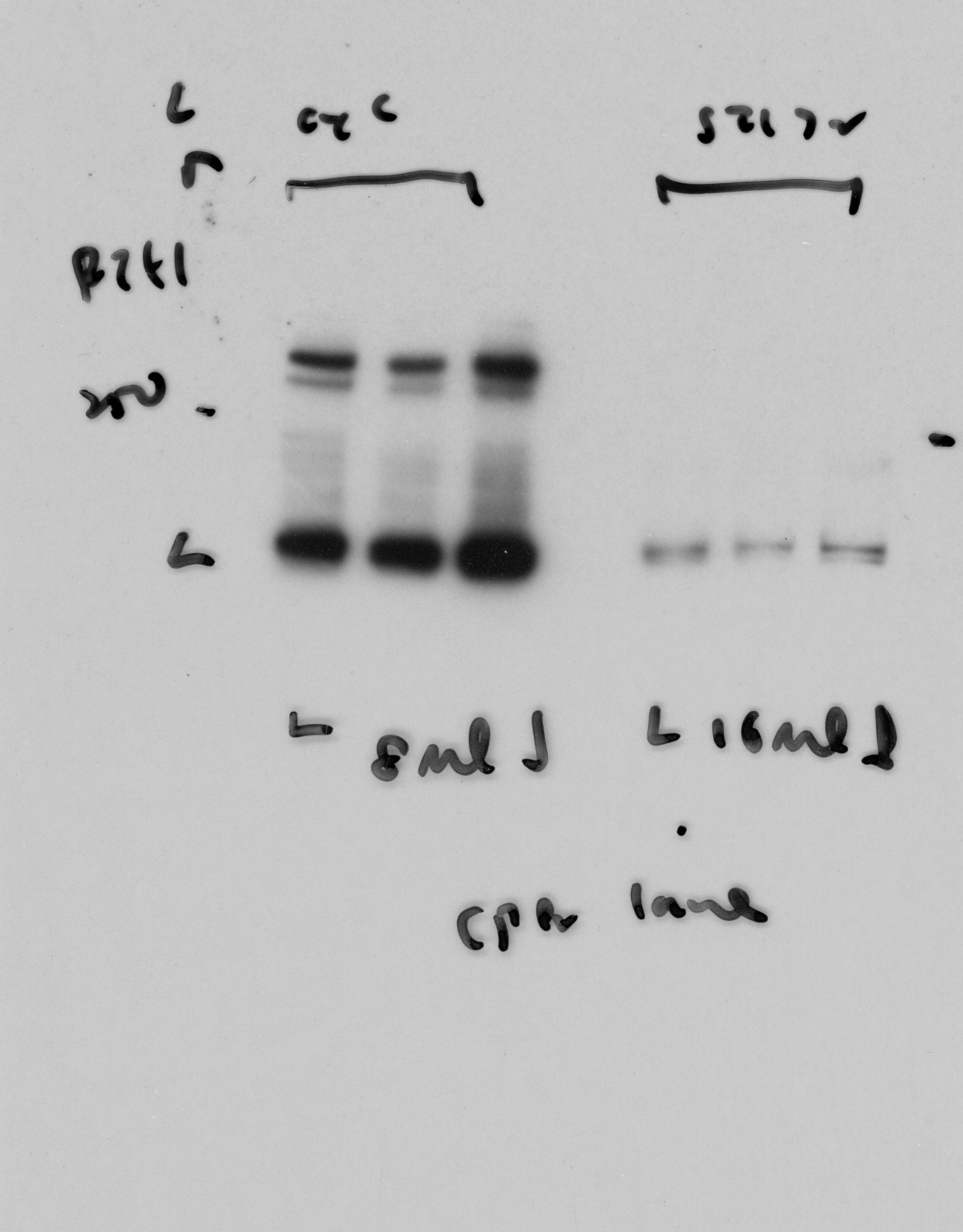

Supplement: Source data 3. [file elife-68466-data3.zip › Source data 3 - figure 4/Figure 4/083120_Rif1_Fig 4A0001.tif]

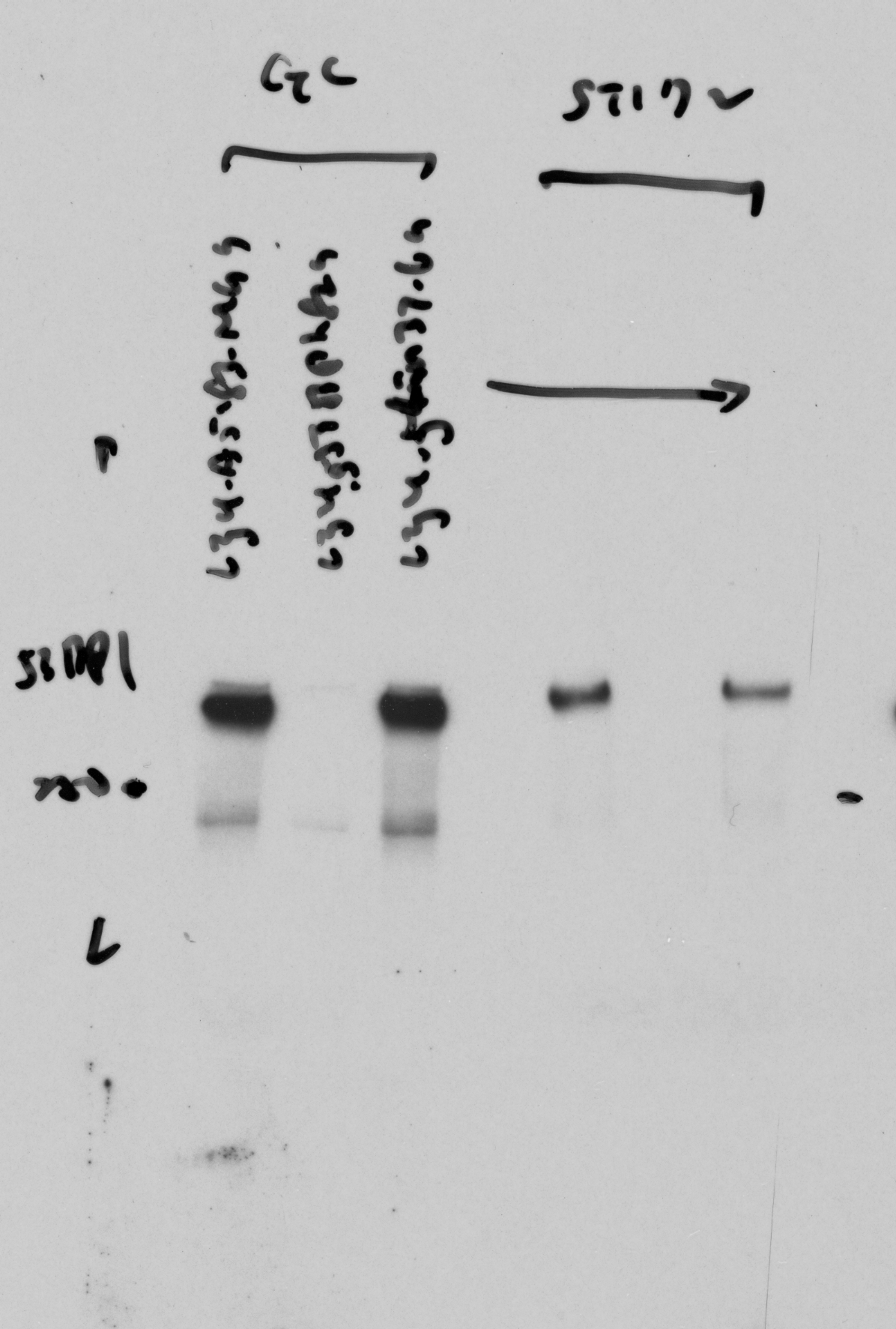

Supplement: Source data 3. [file elife-68466-data3.zip › Source data 3 - figure 4/Figure 4/083120_53bp1_Fig 4A.tif]

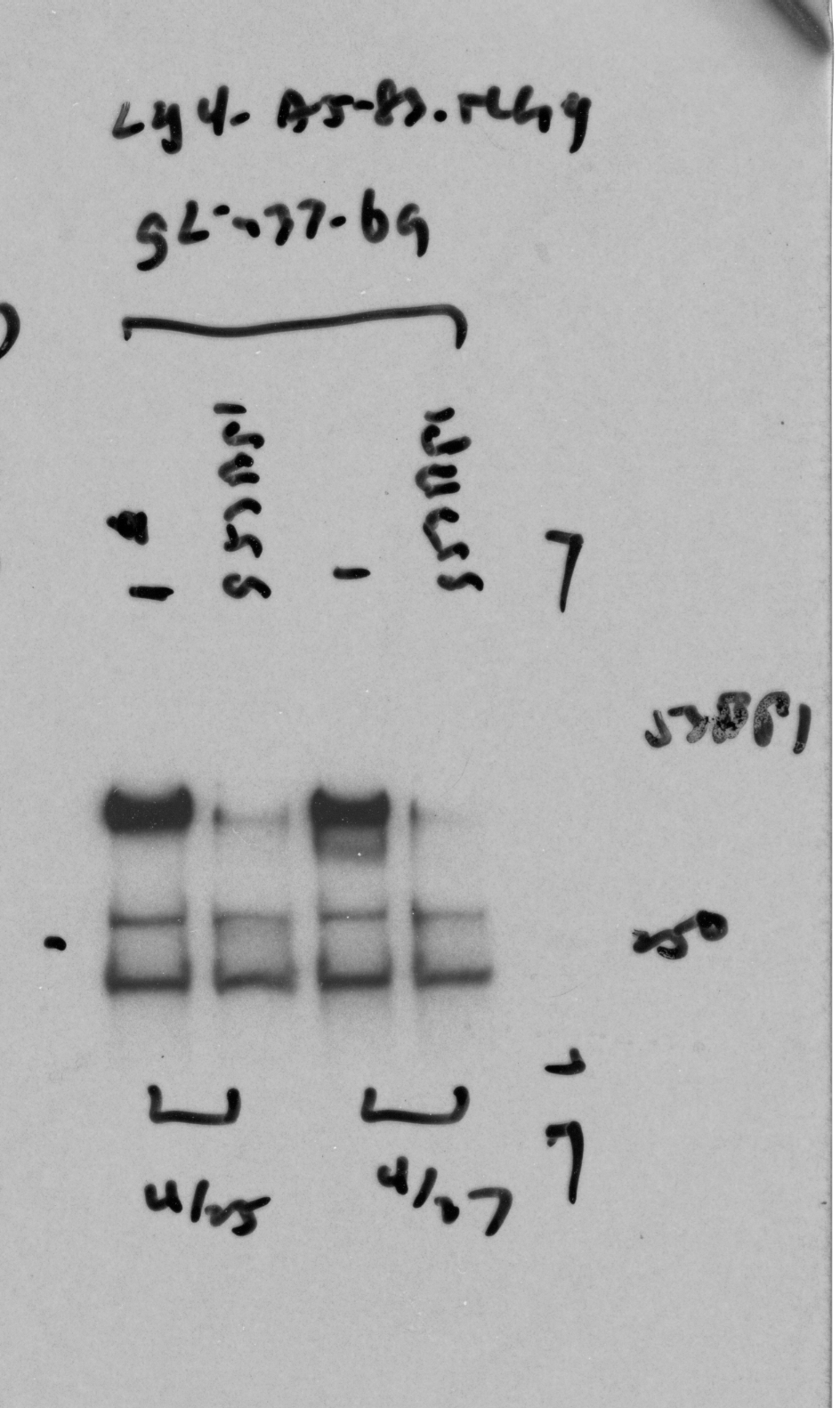

Supplement: Source data 3. [file elife-68466-data3.zip › Source data 3 - figure 4/Figure 4/043020_53bp1_Fig 4E.tif]

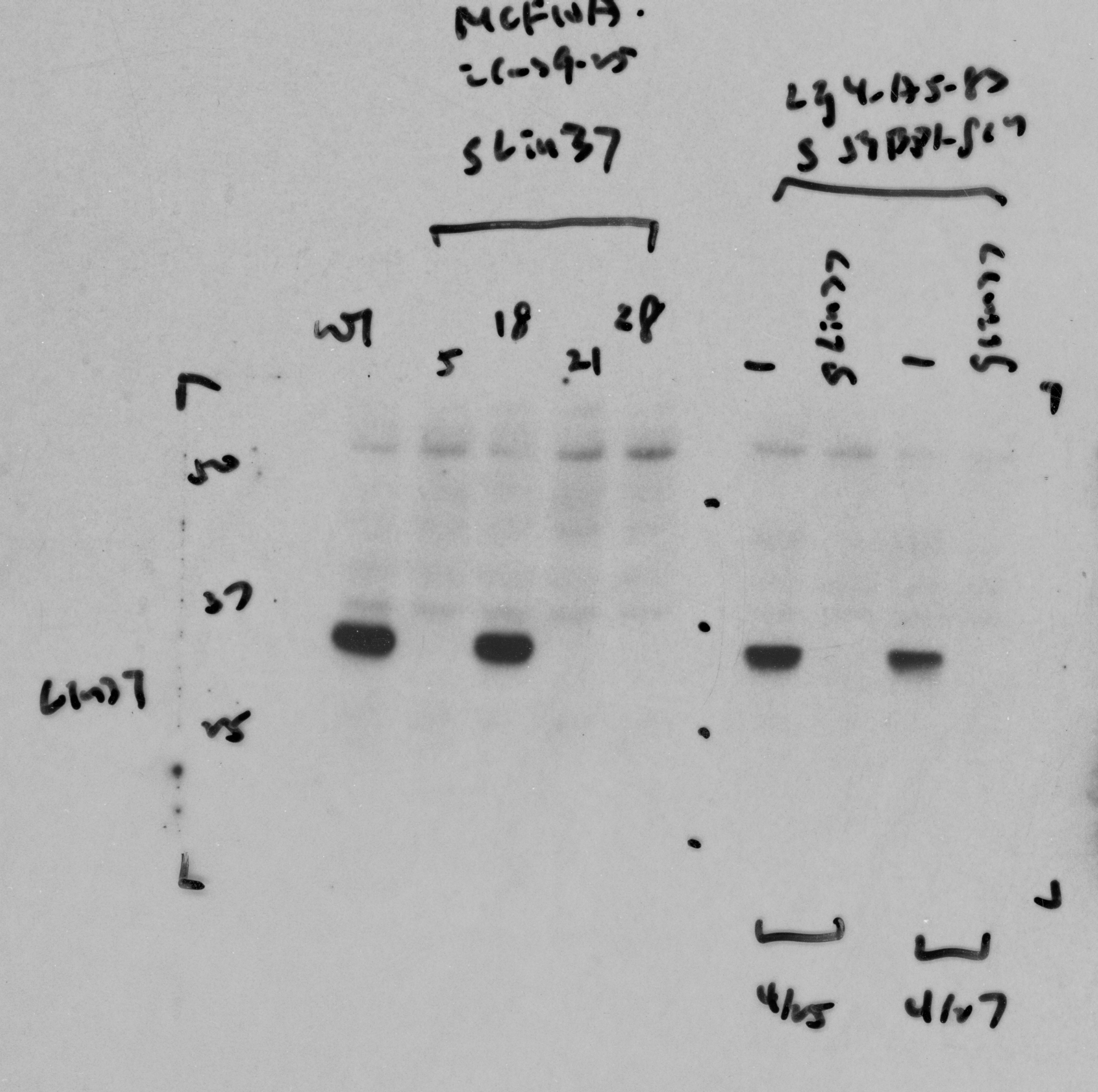

Supplement: Source data 3. [file elife-68466-data3.zip › Source data 3 - figure 4/Figure 4/043020_Lin37_Fig 4D.tif]

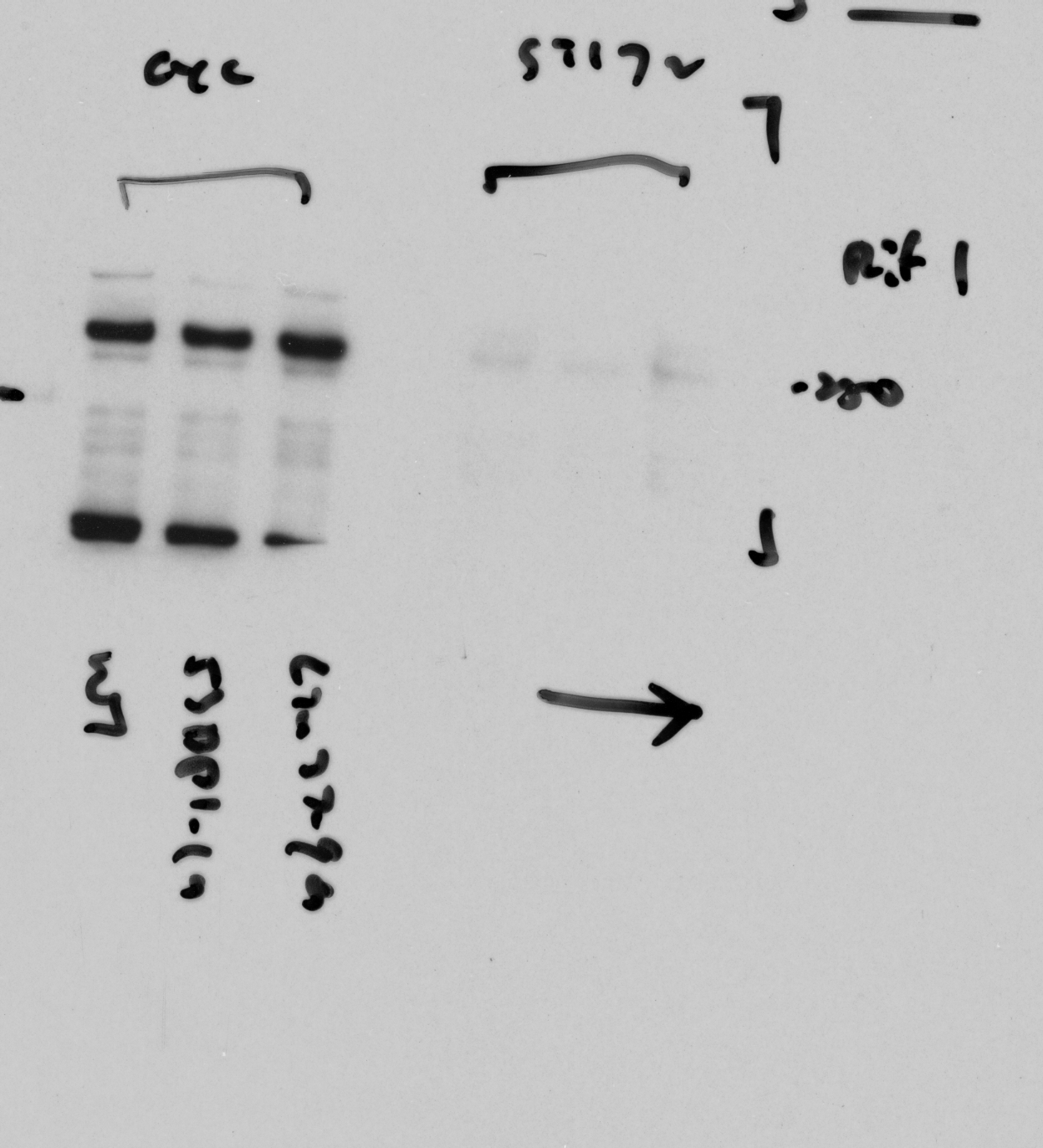

Supplement: Source data 3. [file elife-68466-data3.zip › Source data 3 - figure 4/Figure 4/083120_Rif1_Fig 4 S1A0001.tif]

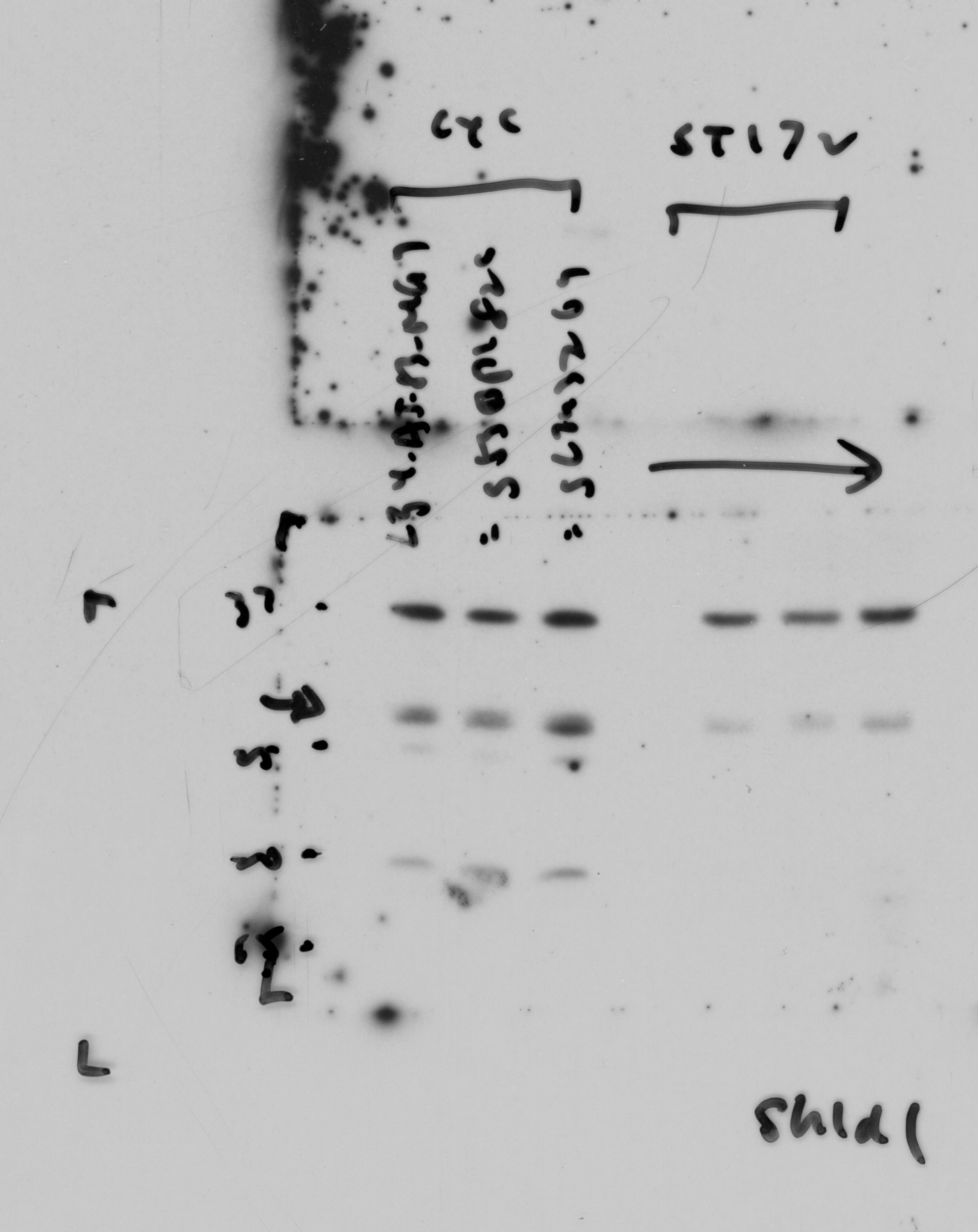

Supplement: Source data 3. [file elife-68466-data3.zip › Source data 3 - figure 4/Figure 4/083120_shld1_Fig 4A.tif]

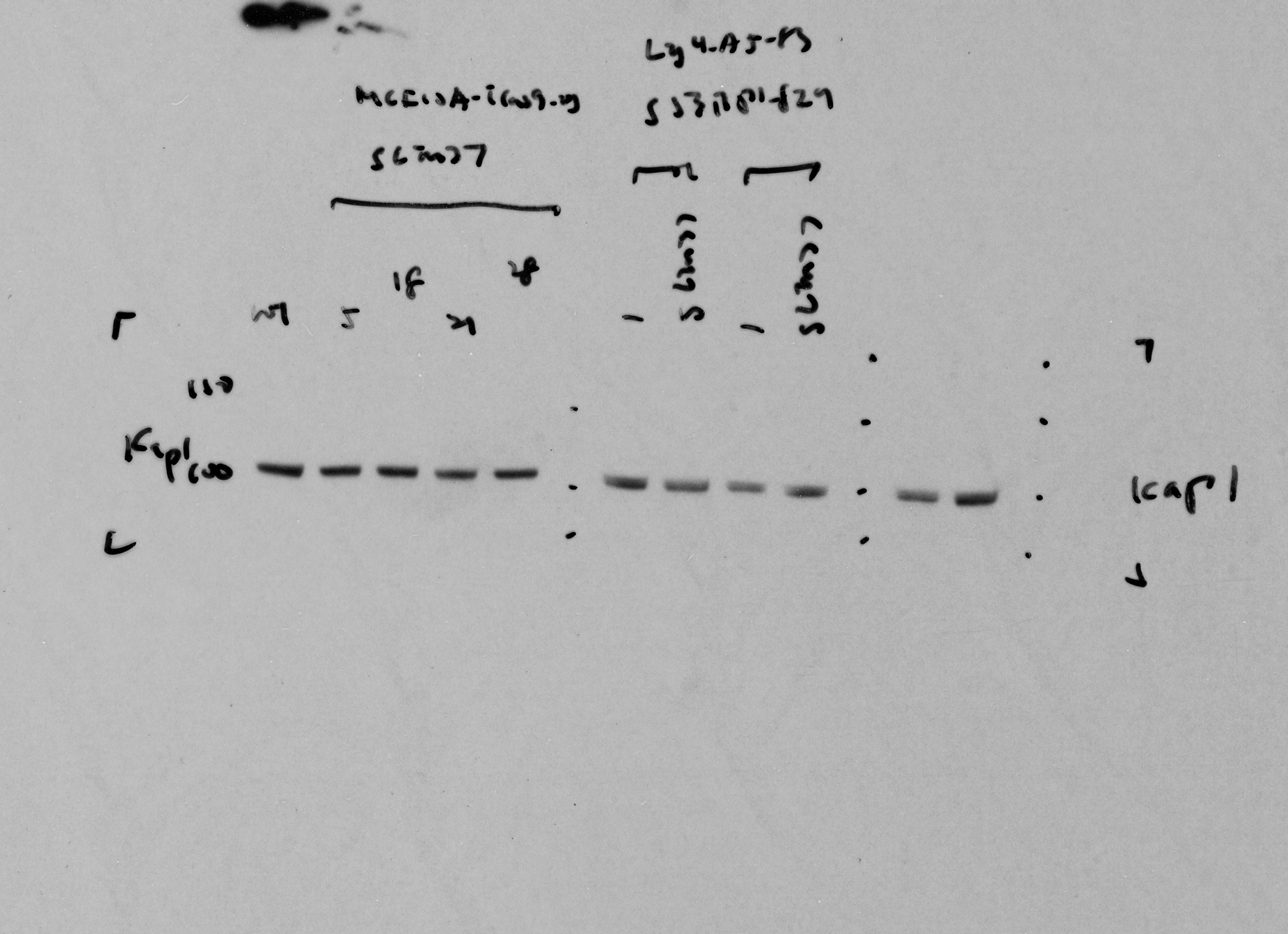

Supplement: Source data 3. [file elife-68466-data3.zip › Source data 3 - figure 4/Figure 4/043020_Kap1_Fig 4D.tif]

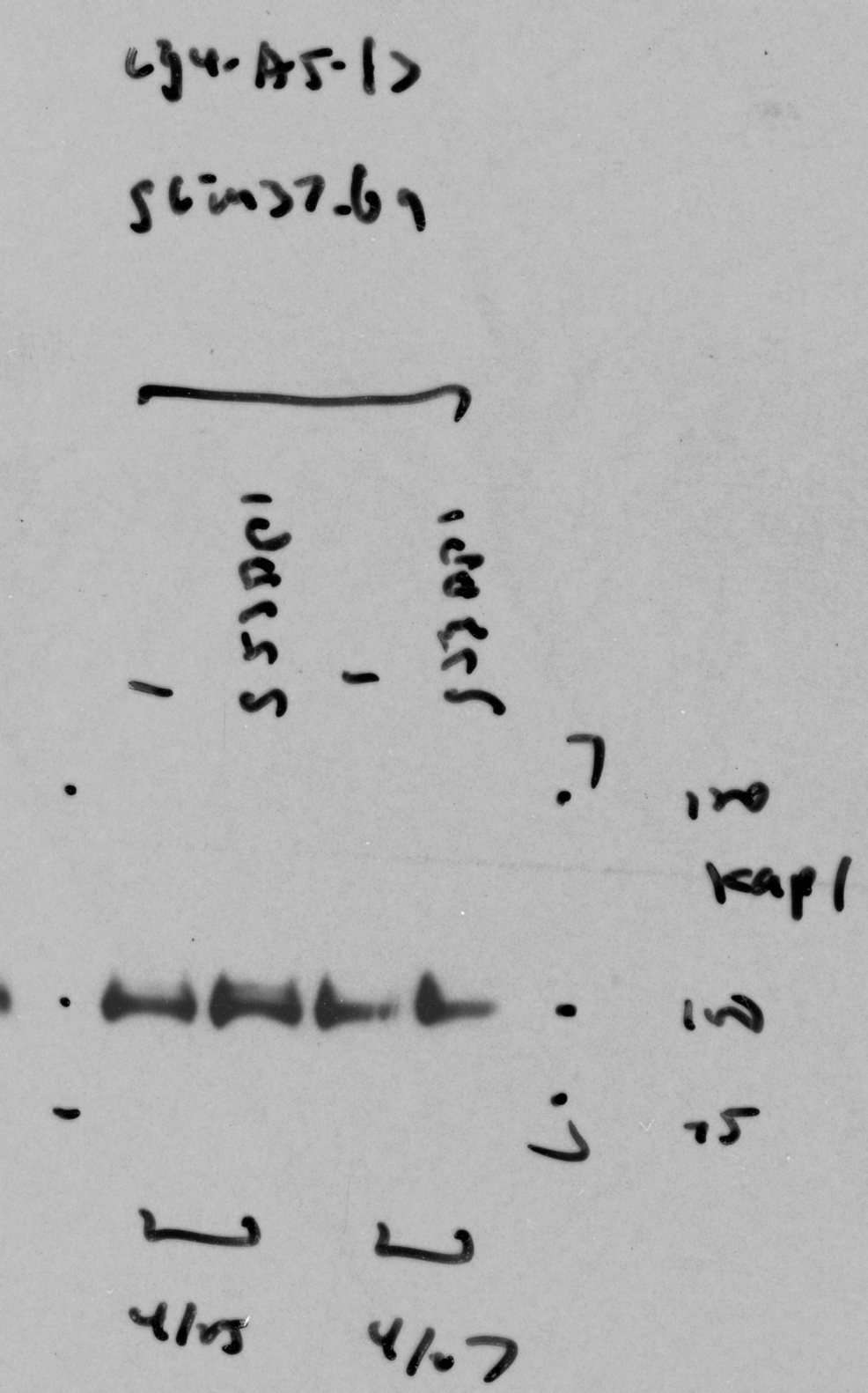

Supplement: Source data 3. [file elife-68466-data3.zip › Source data 3 - figure 4/Figure 4/043020_KAP1_Fig 4E.tif]

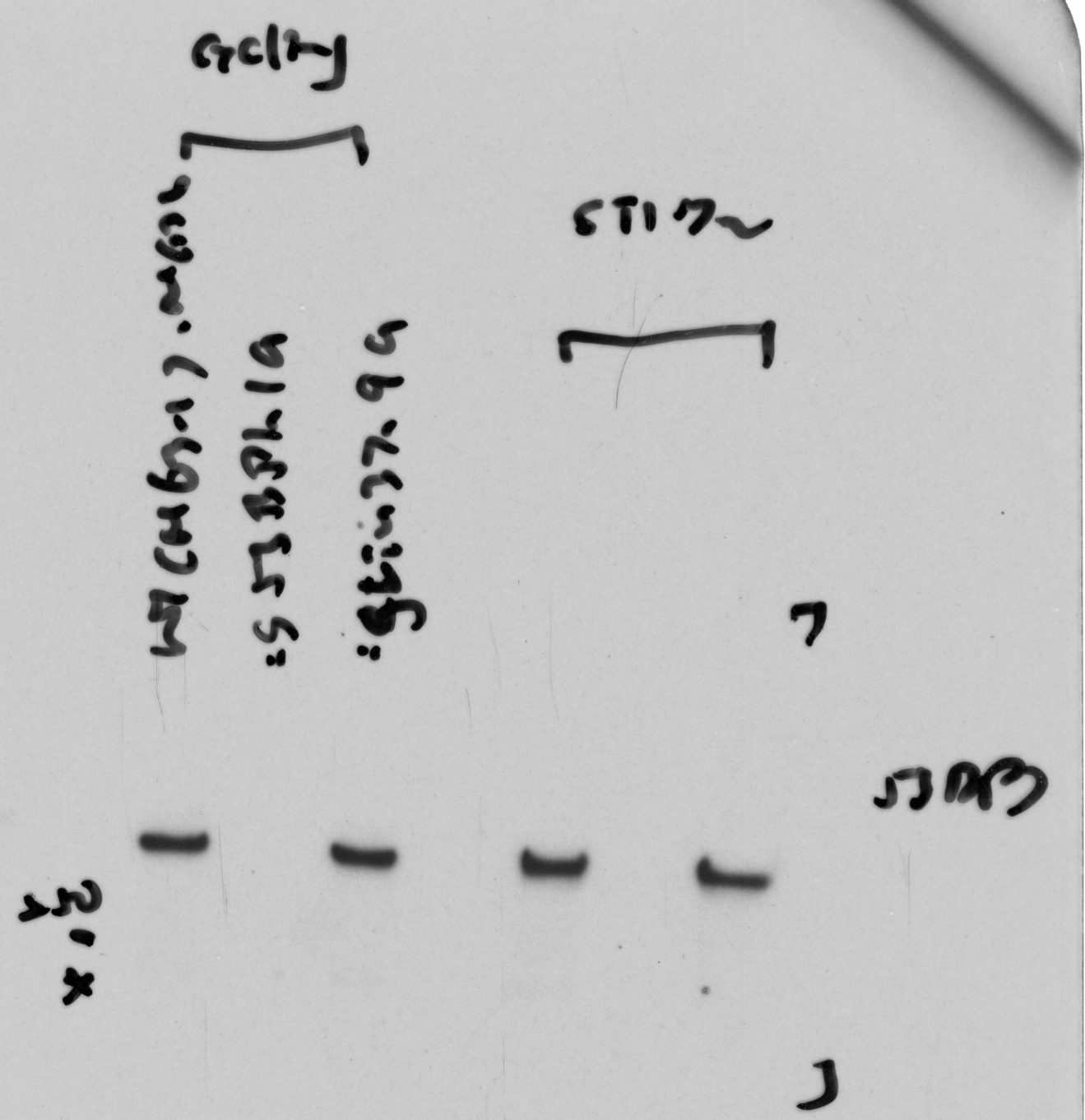

Supplement: Source data 3. [file elife-68466-data3.zip › Source data 3 - figure 4/Figure 4/083120_53bp1_Fig 4 S1A.tif]

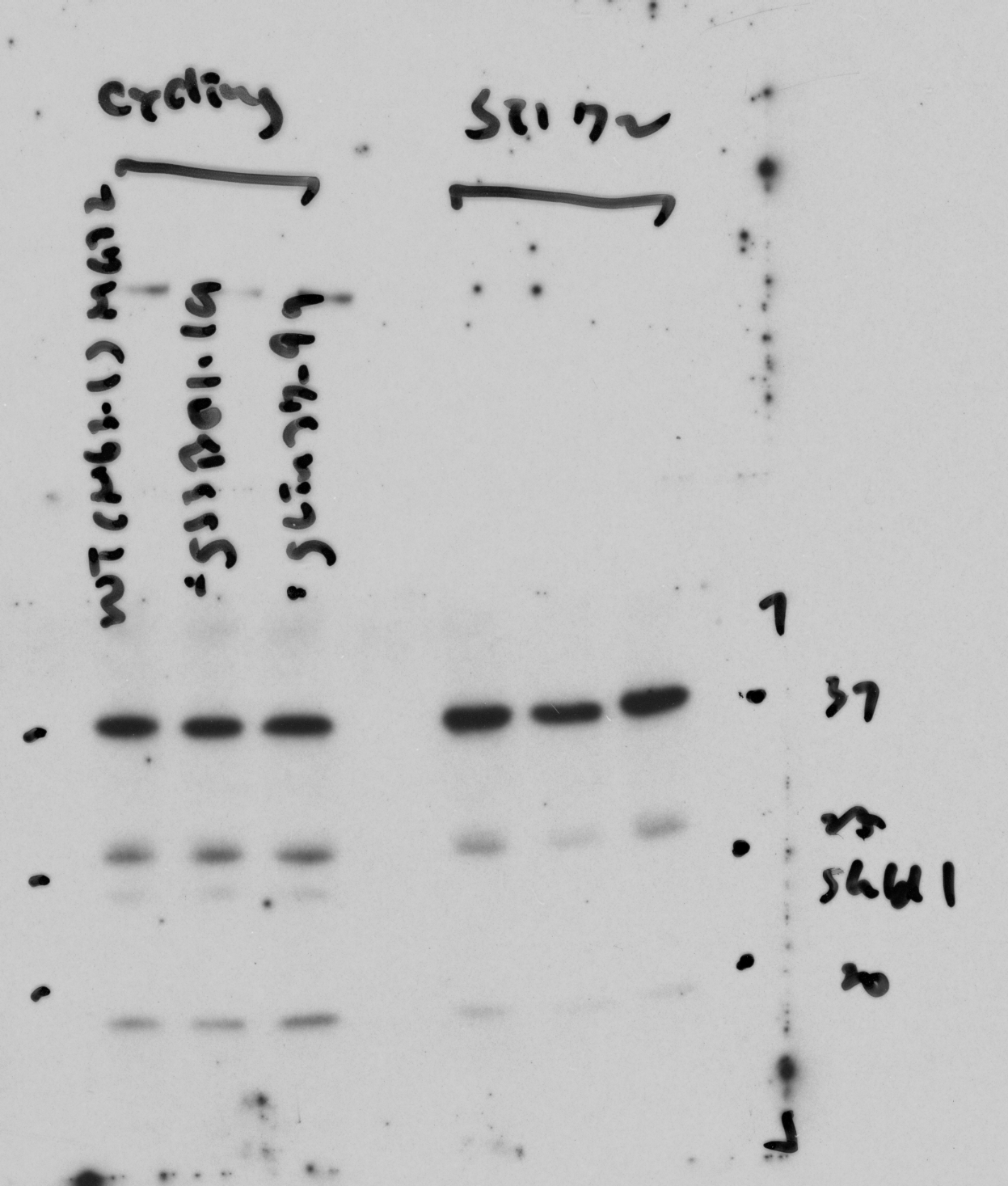

Supplement: Source data 3. [file elife-68466-data3.zip › Source data 3 - figure 4/Figure 4/083120_Shld1_Fig 4 S1A.tif]

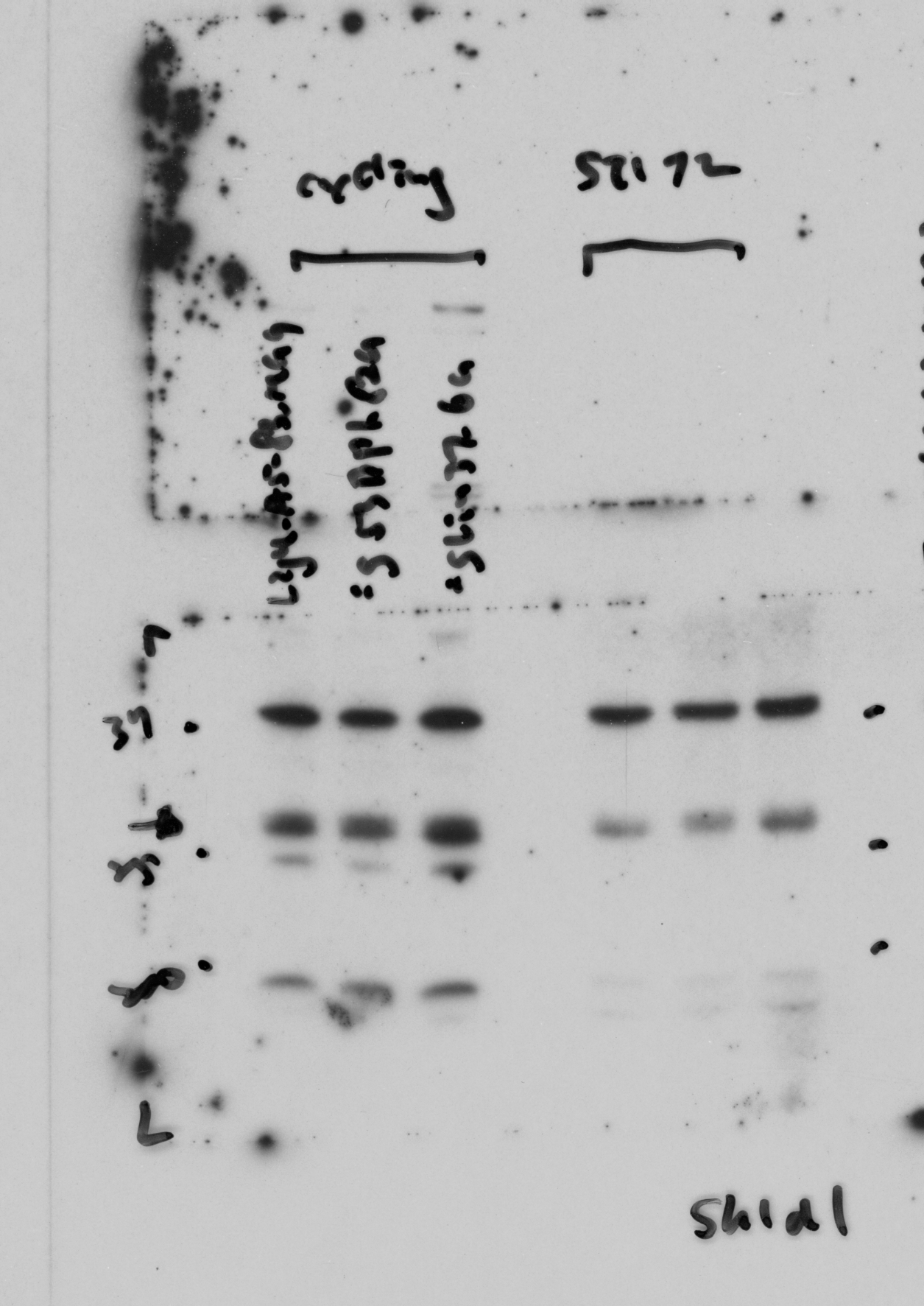

Supplement: Source data 3. [file elife-68466-data3.zip › Source data 3 - figure 4/Figure 4/083120_shld1_Fig 4A0001.tif]

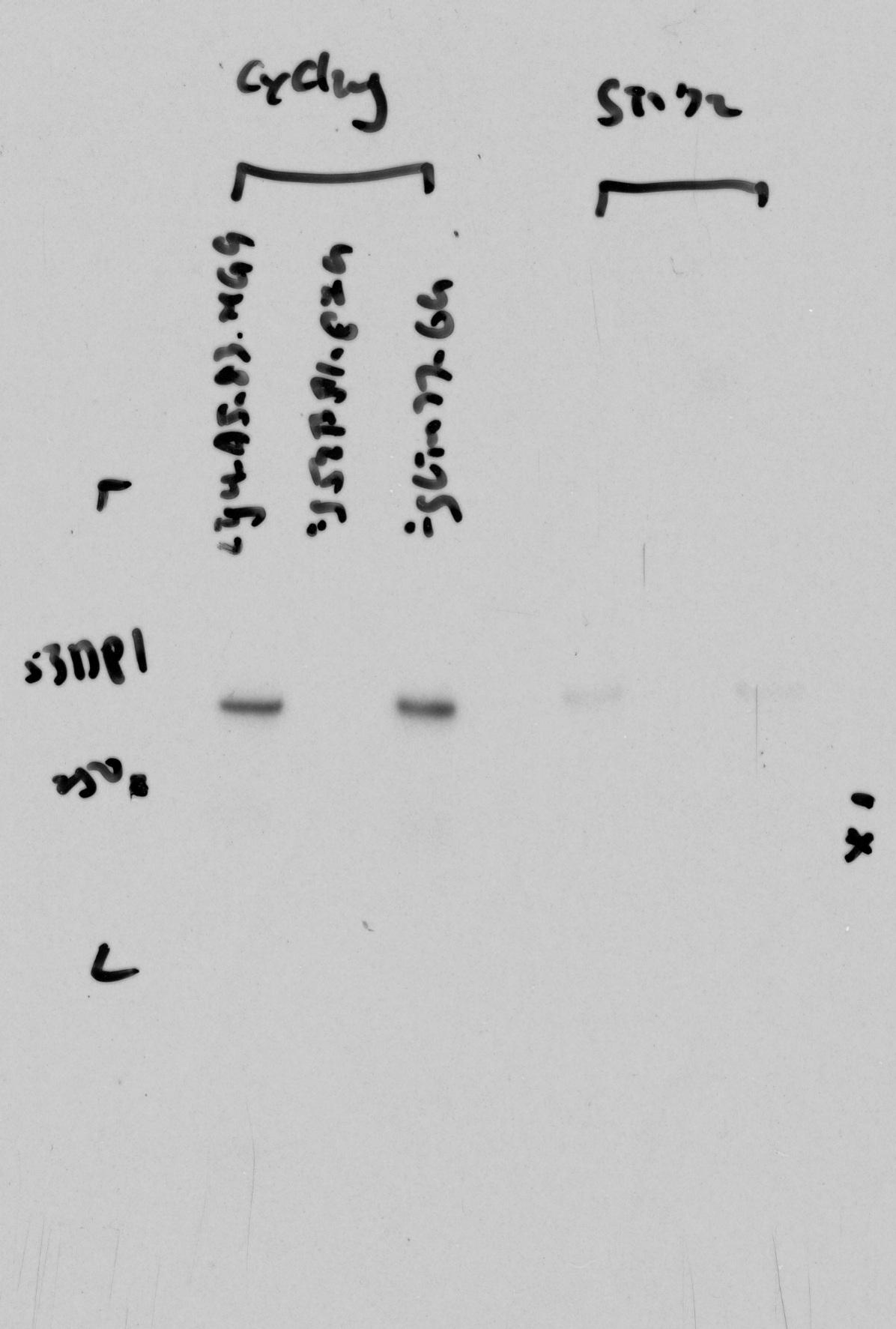

Supplement: Source data 3. [file elife-68466-data3.zip › Source data 3 - figure 4/Figure 4/083120_53bp1_Fig 4A0001.tif]

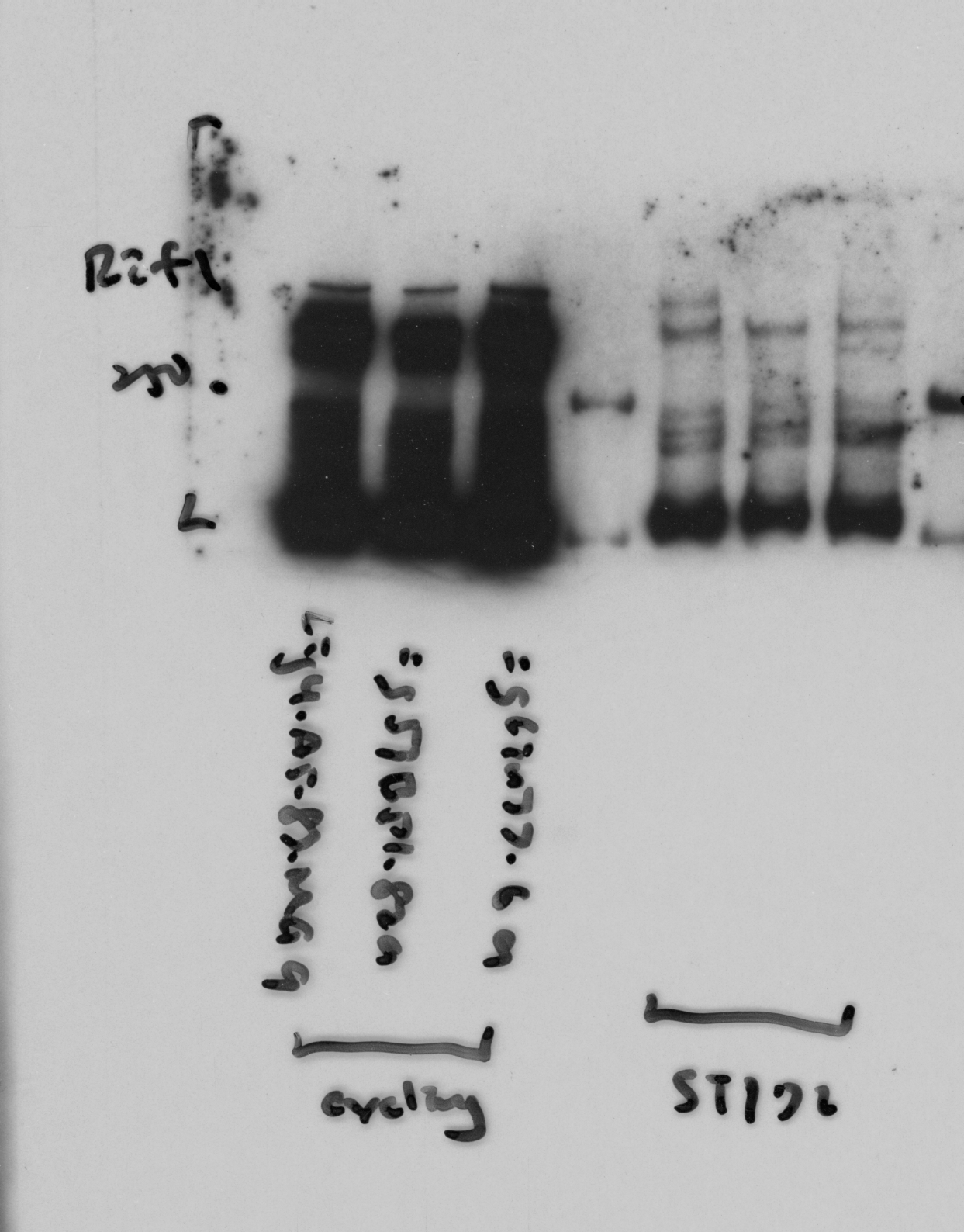

Supplement: Source data 3. [file elife-68466-data3.zip › Source data 3 - figure 4/Figure 4/083120_Rif1_Fig 4A.tif]

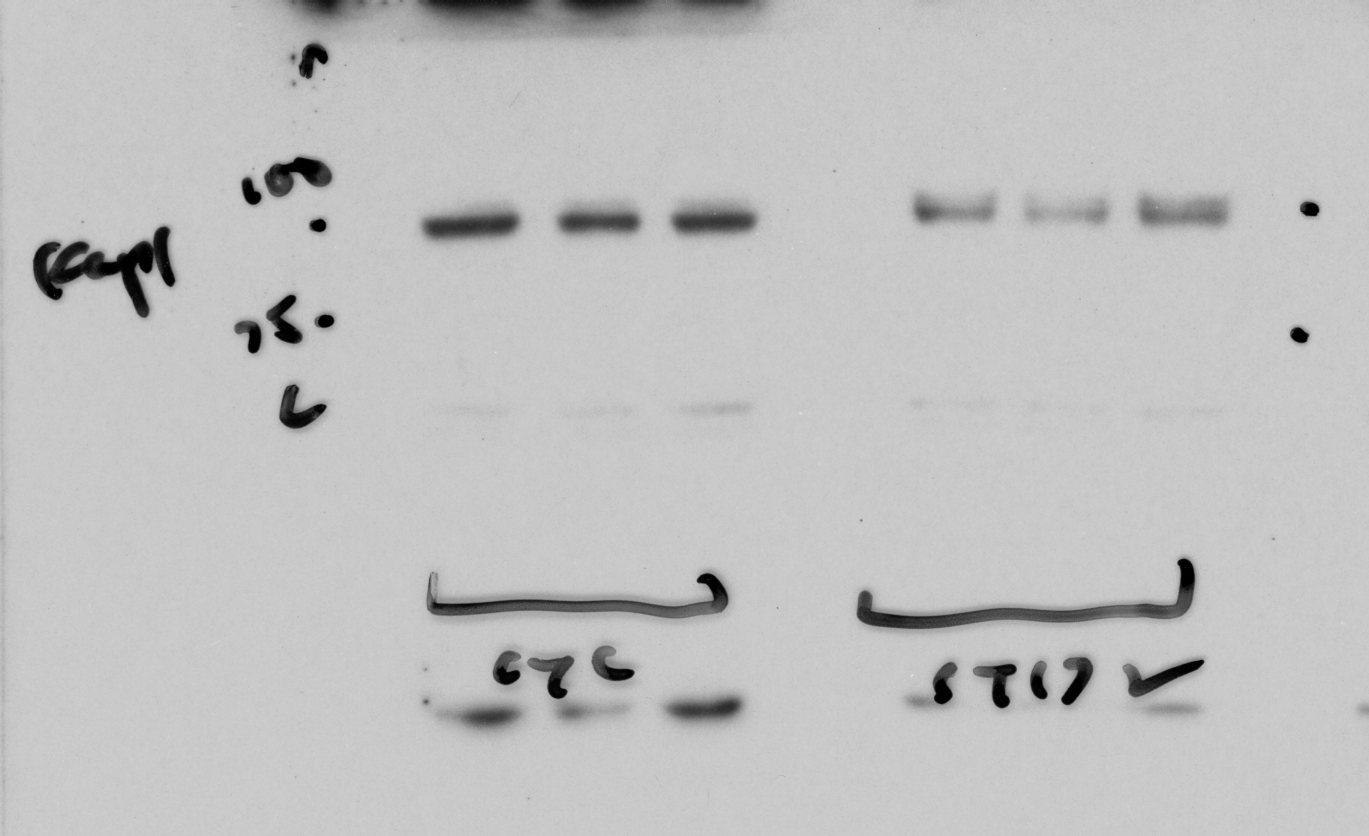

Supplement: Source data 3. [file elife-68466-data3.zip › Source data 3 - figure 4/Figure 4/083120_Kap1_Fig 4A.tif]

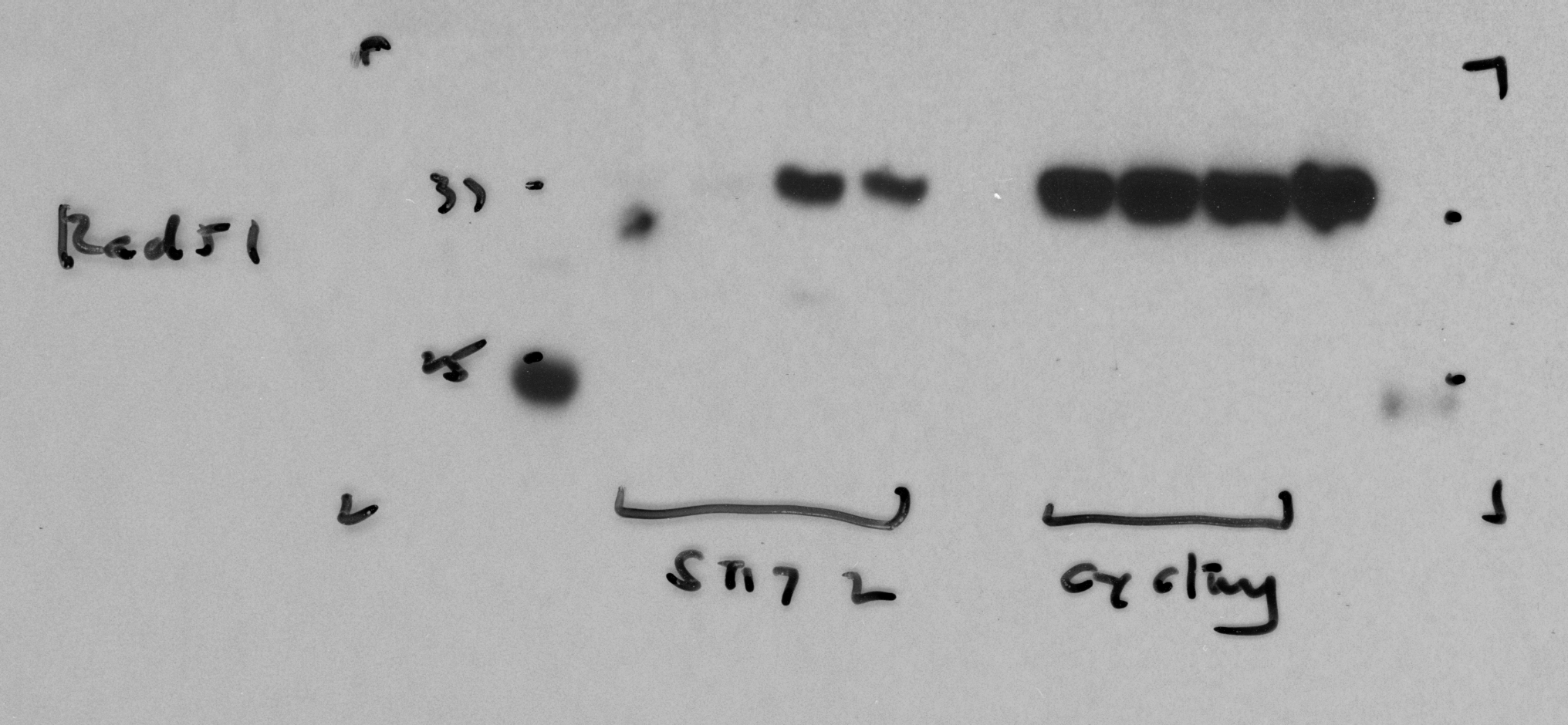

Supplement: Source data 4. [file elife-68466-data4.zip › Source data 4 - figure 5 part 1/Figure 5/032020_RAD51_Fig 5C.tif]

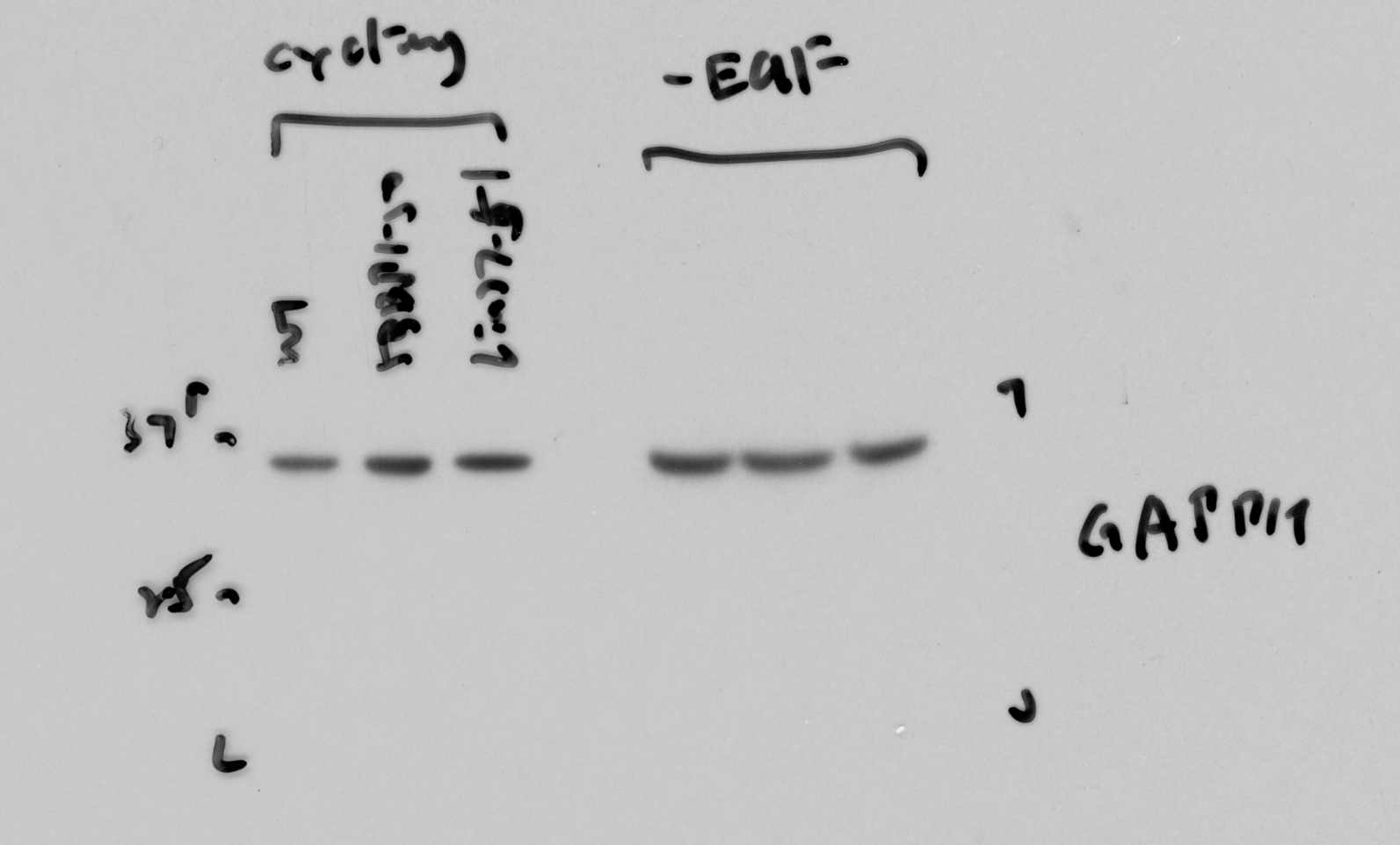

Supplement: Source data 4. [file elife-68466-data4.zip › Source data 4 - figure 5 part 1/Figure 5/061220_GAPDH_Fig 5D.tif]

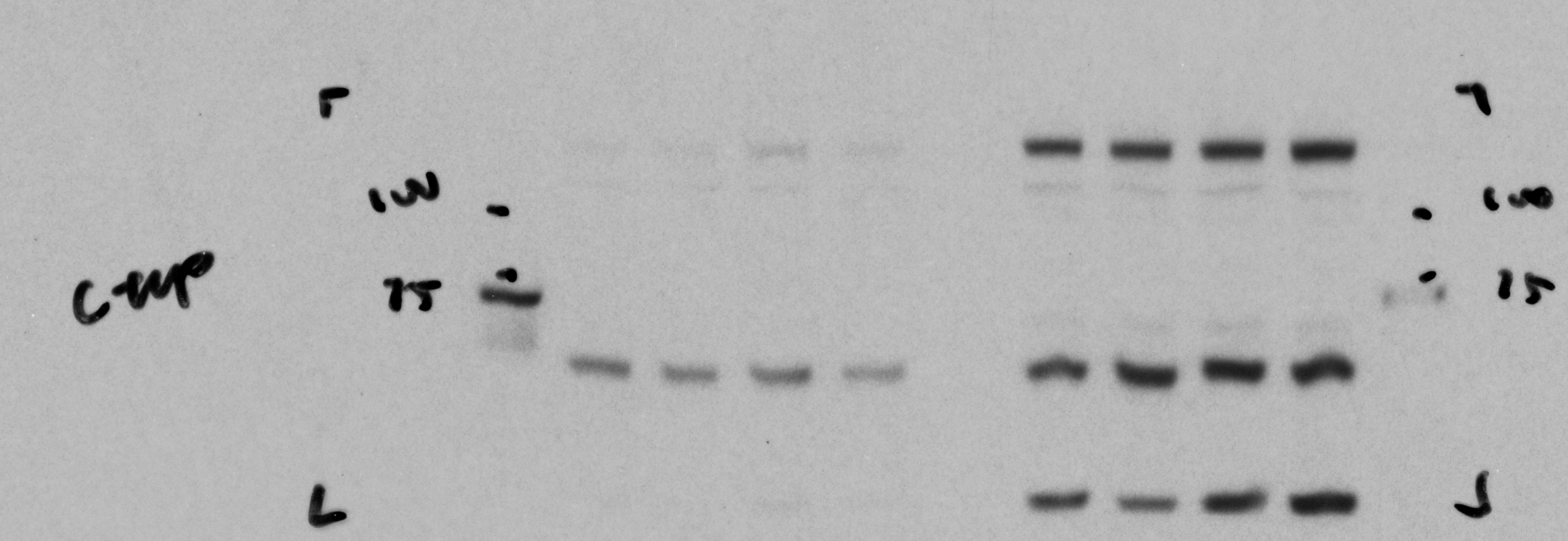

Supplement: Source data 4. [file elife-68466-data4.zip › Source data 4 - figure 5 part 1/Figure 5/032020_5_CtIP_Fig 5C.tif]

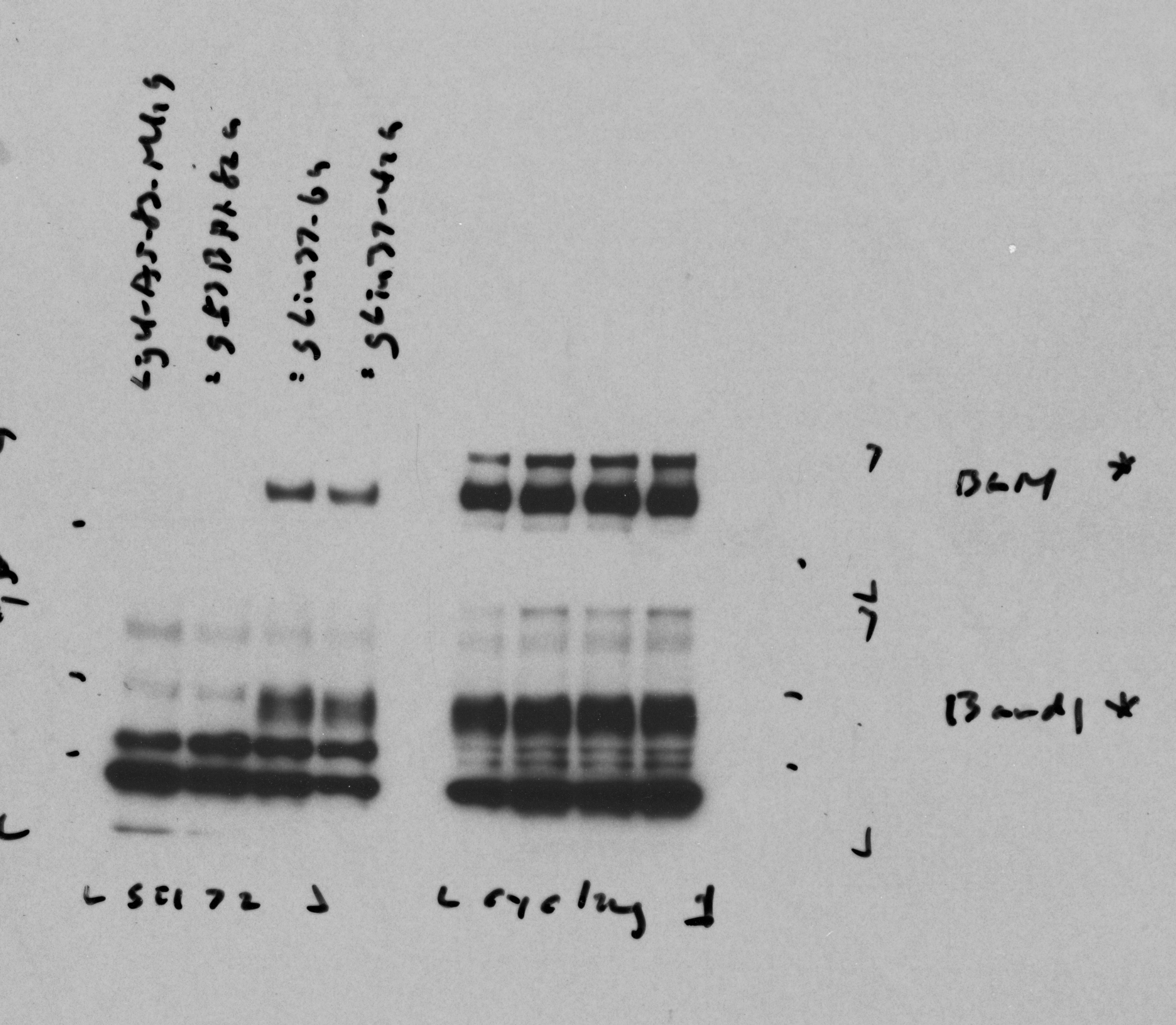

Supplement: Source data 4. [file elife-68466-data4.zip › Source data 4 - figure 5 part 1/Figure 5/031920_BLM-Bard1_Fig 5C0001.tif]

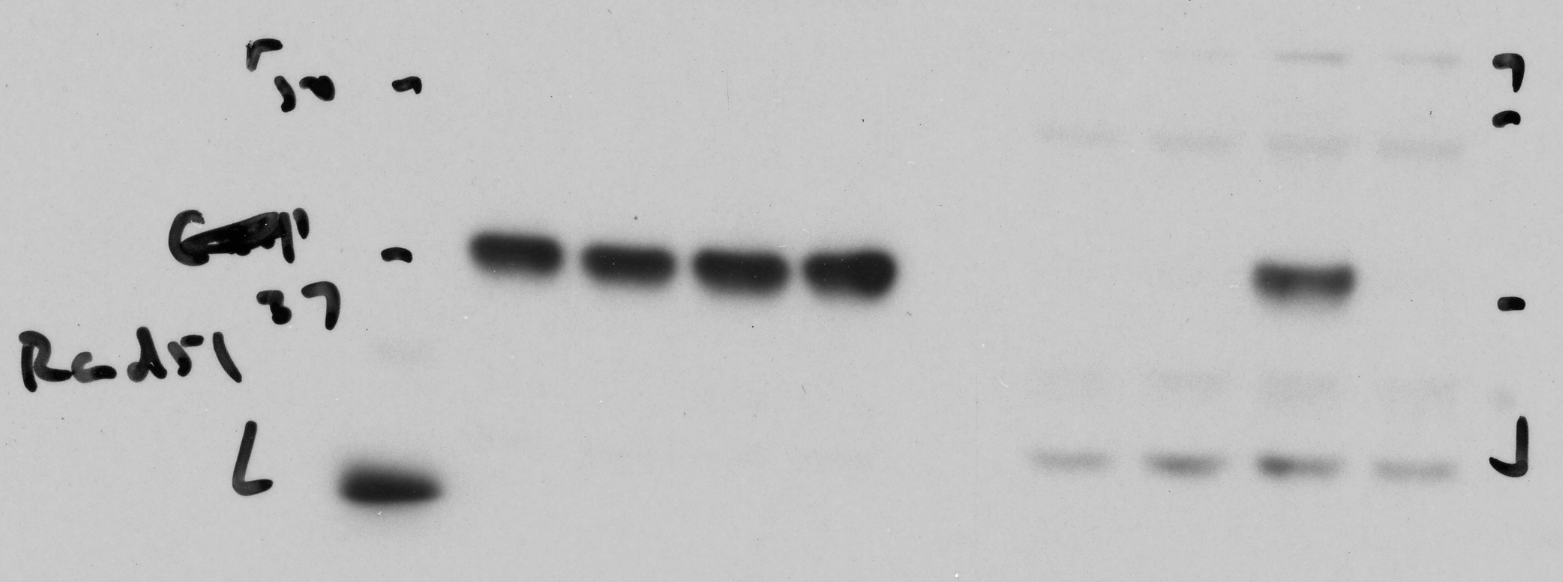

Supplement: Source data 4. [file elife-68466-data4.zip › Source data 4 - figure 5 part 1/Figure 5/0608200003_RAD51_Fig 5D.tif]

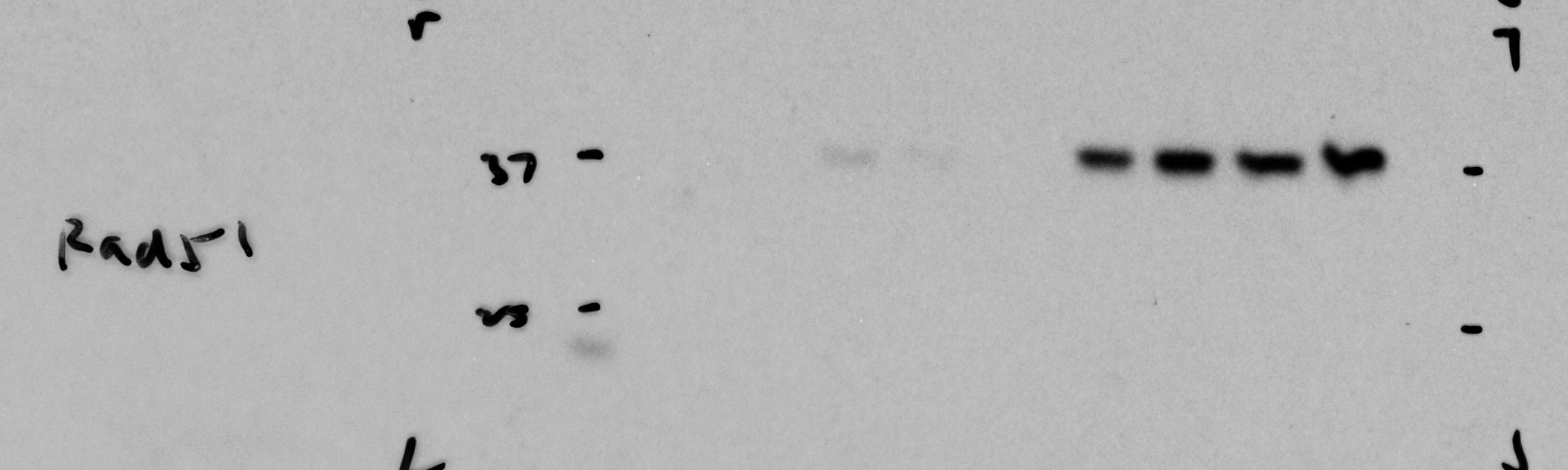

Supplement: Source data 4. [file elife-68466-data4.zip › Source data 4 - figure 5 part 1/Figure 5/032020_7_RAD51_Fig 5C.tif]

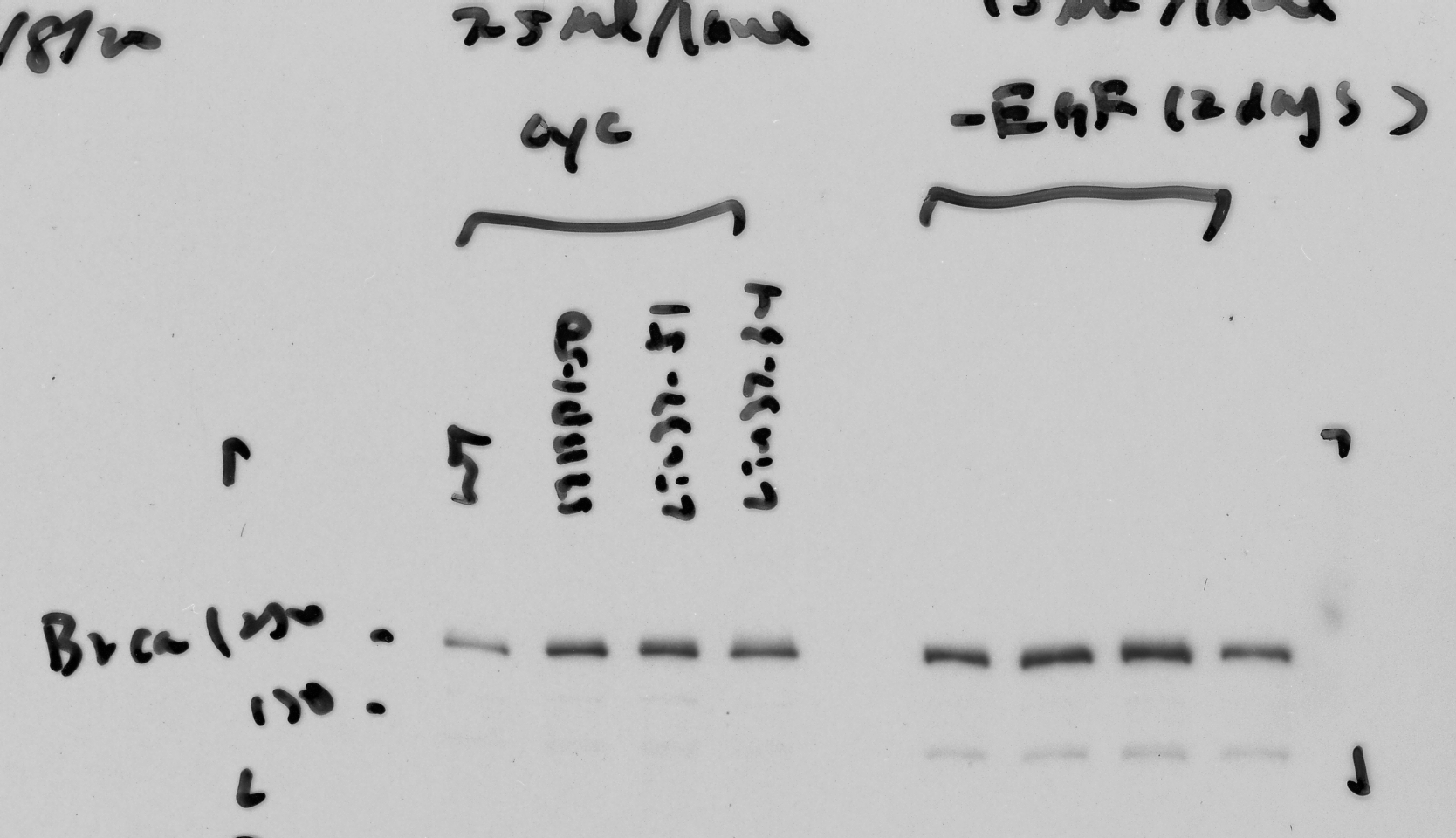

Supplement: Source data 4. [file elife-68466-data4.zip › Source data 4 - figure 5 part 1/Figure 5/060820_BRCA1_FIg 5D.tif]

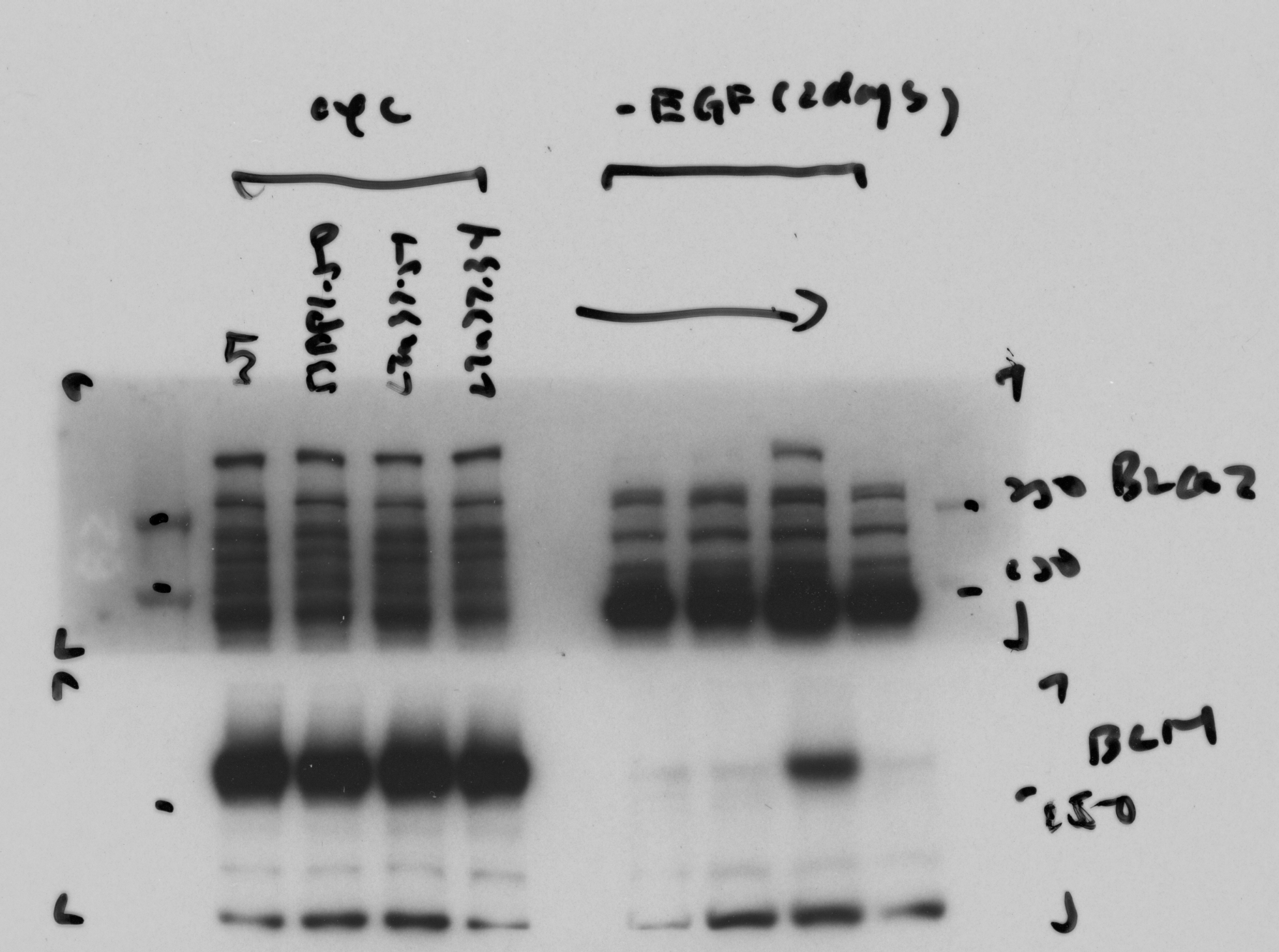

Supplement: Source data 4. [file elife-68466-data4.zip › Source data 4 - figure 5 part 1/Figure 5/060820_BRCA2, BLM_Fig 5D.tif]

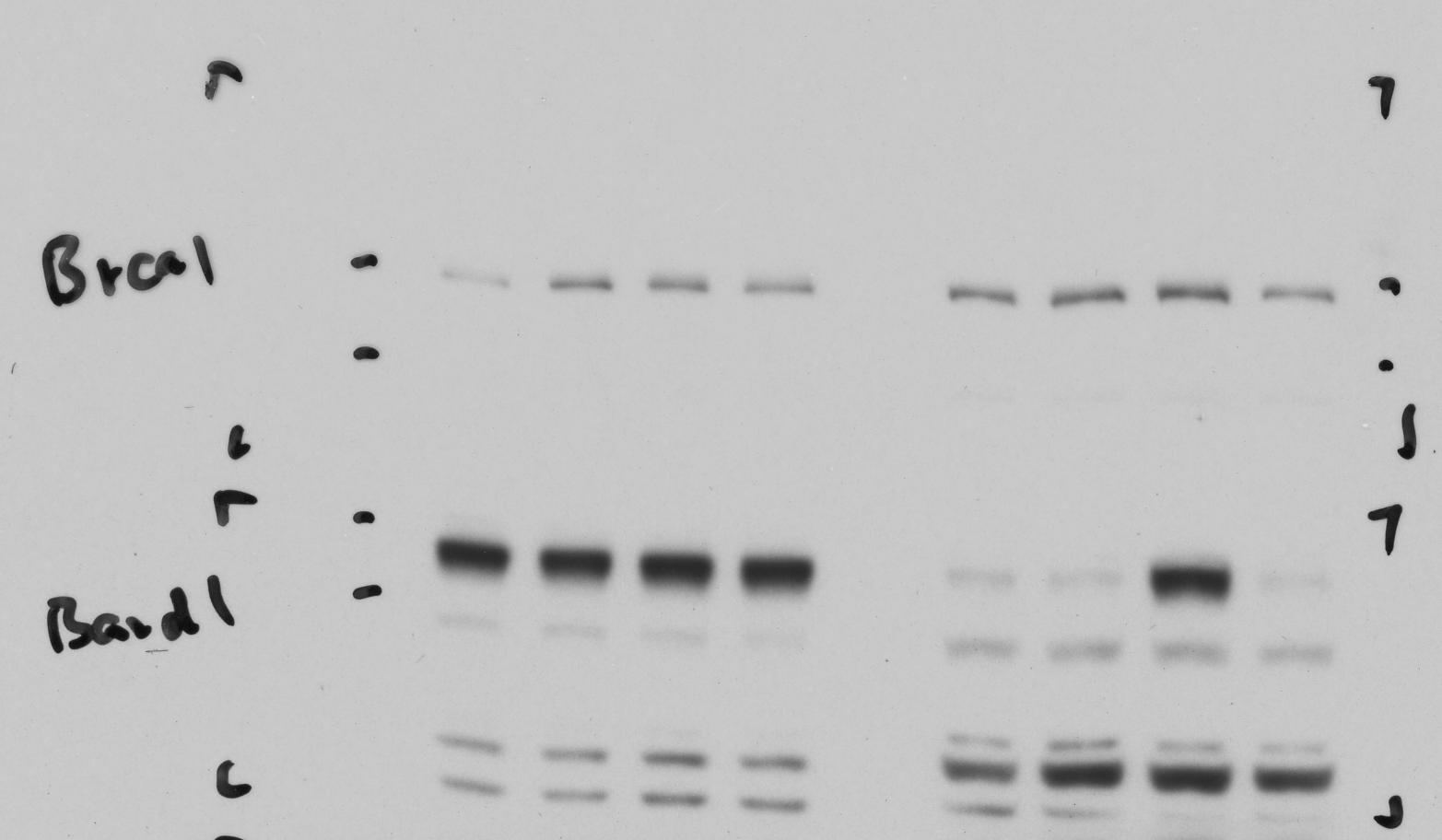

Supplement: Source data 4. [file elife-68466-data4.zip › Source data 4 - figure 5 part 1/Figure 5/0608200001_BRCA1, Bard1_Fig 5D.tif]

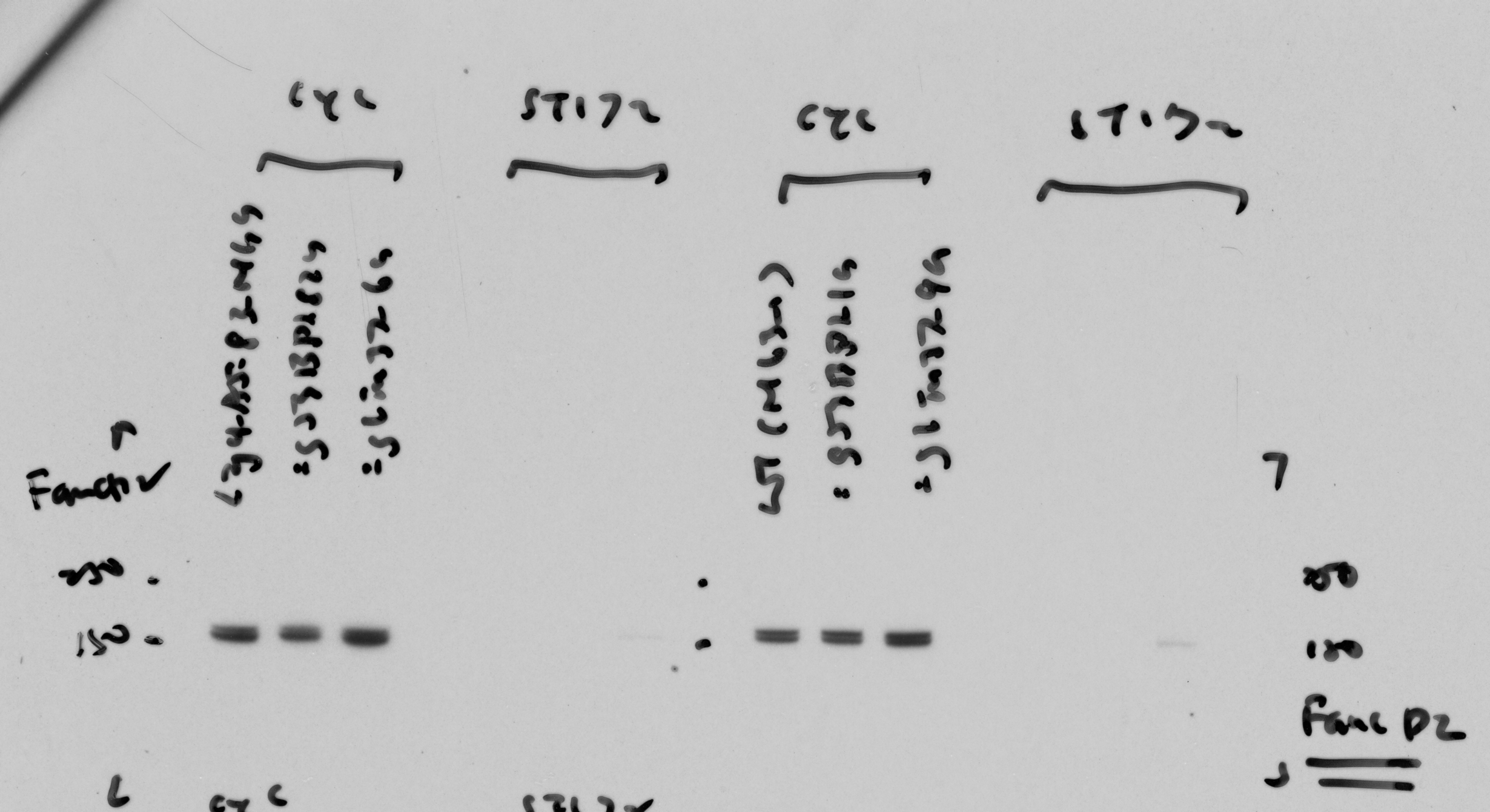

Supplement: Source data 4. [file elife-68466-data4.zip › Source data 4 - figure 5 part 1/Figure 5/083120_FANCD2_Fig 5C.tif]

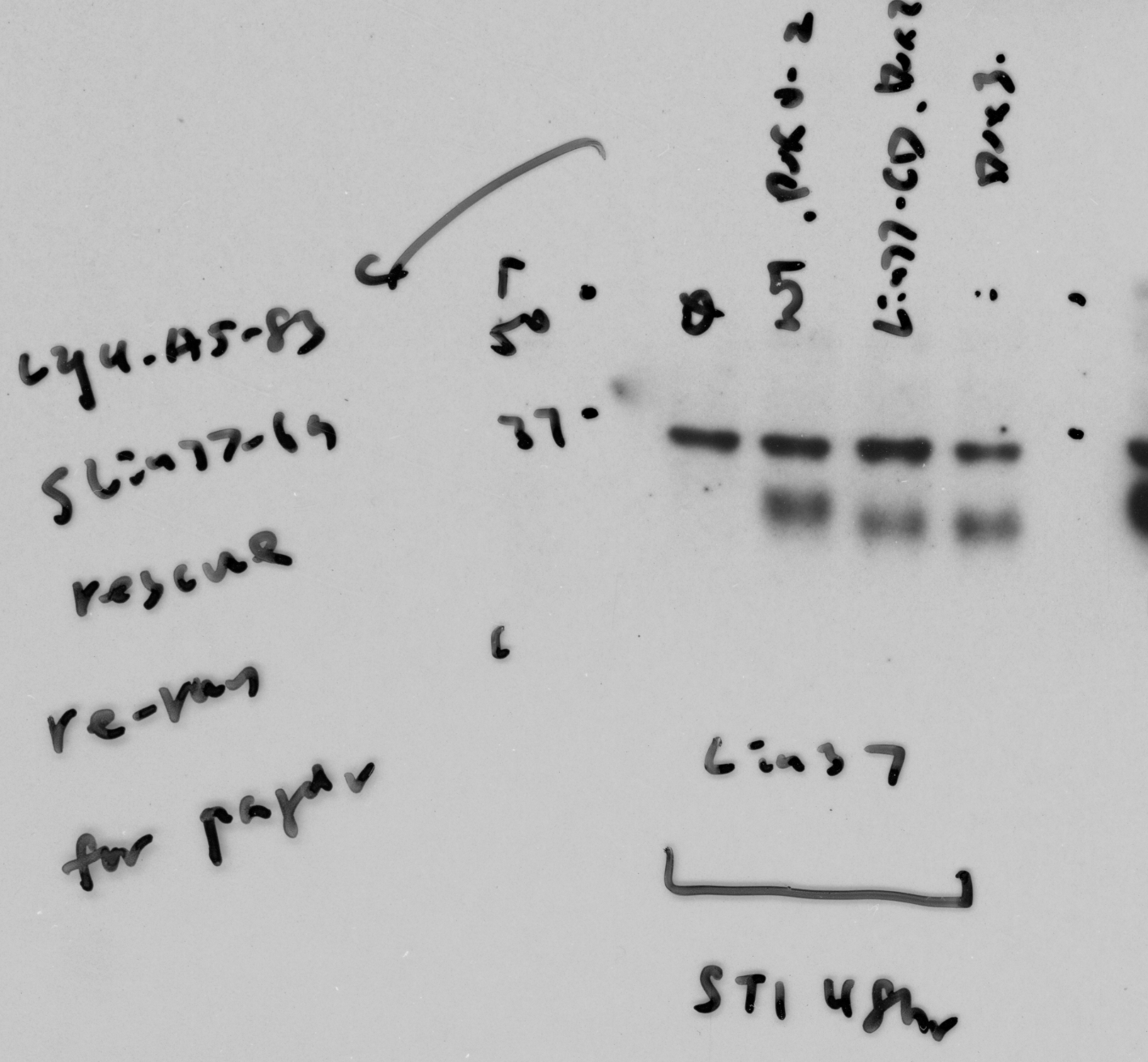

Supplement: Source data 4. [file elife-68466-data4.zip › Source data 4 - figure 5 part 1/Figure 5/0721120_Lin37_Fig 5A.tif]

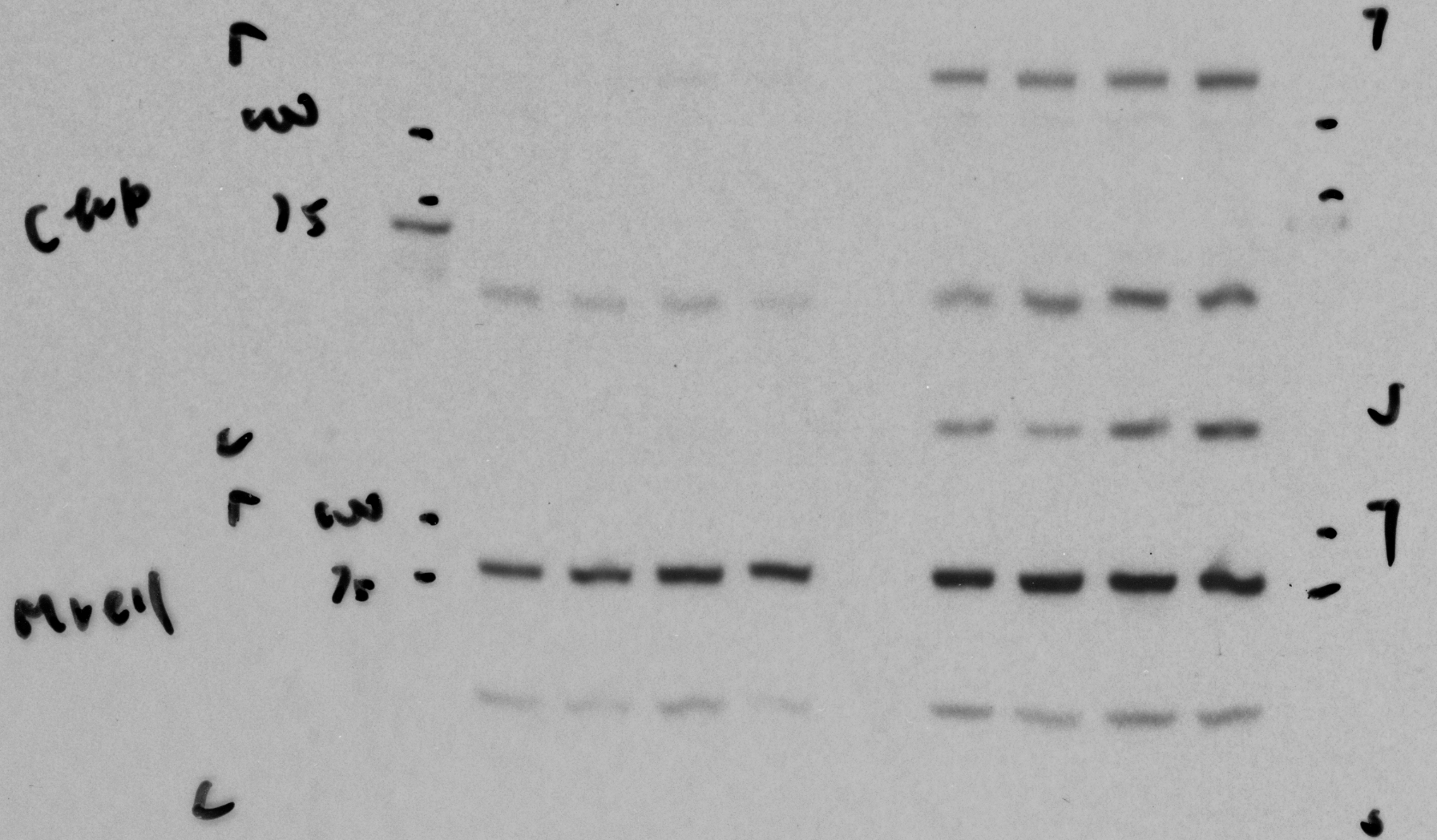

Supplement: Source data 4. [file elife-68466-data4.zip › Source data 4 - figure 5 part 1/Figure 5/032020_6_Mre11+CtIP_Fig 5C.tif]

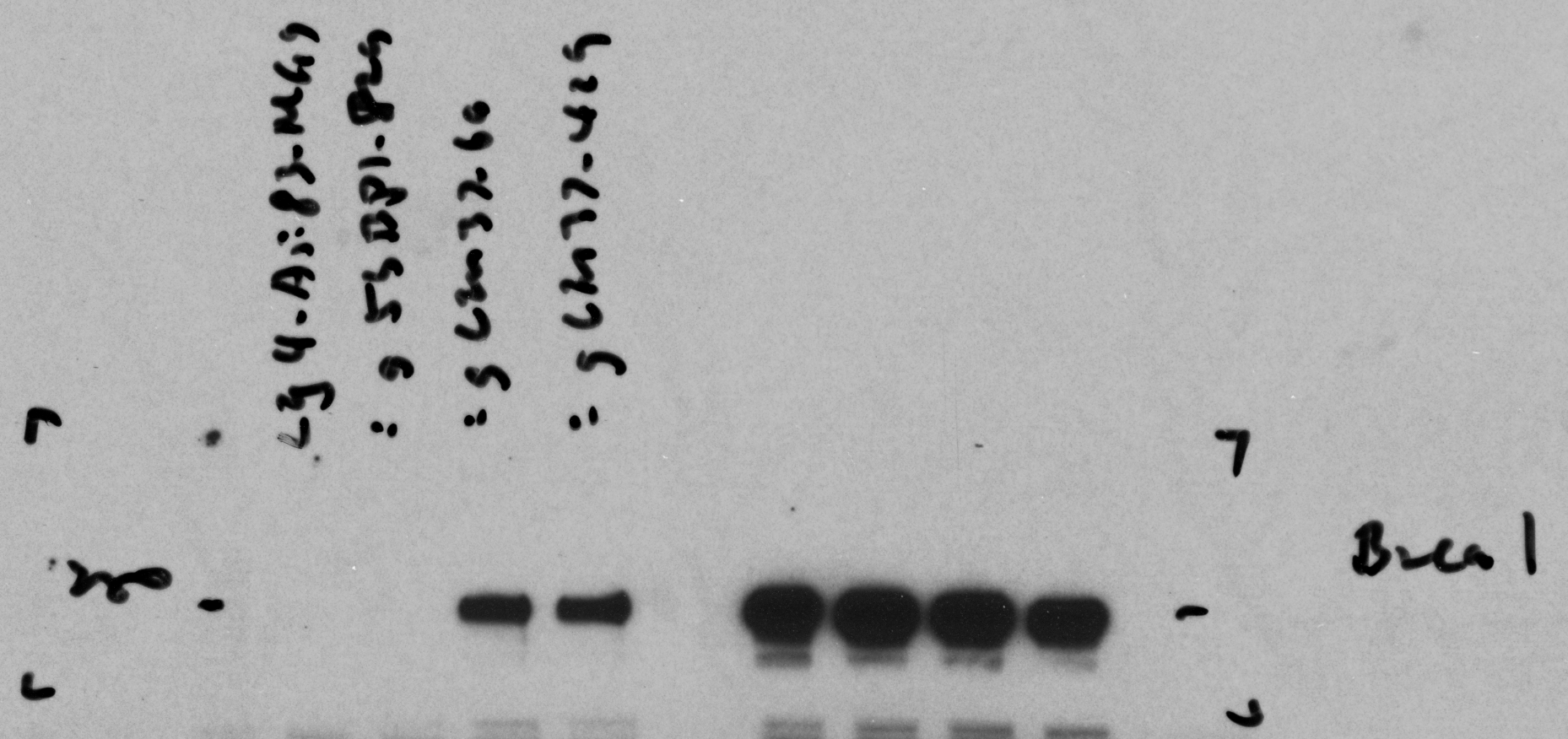

Supplement: Source data 4. [file elife-68466-data4.zip › Source data 4 - figure 5 part 1/Figure 5/032020_Brca1_Fig 5C.tif]

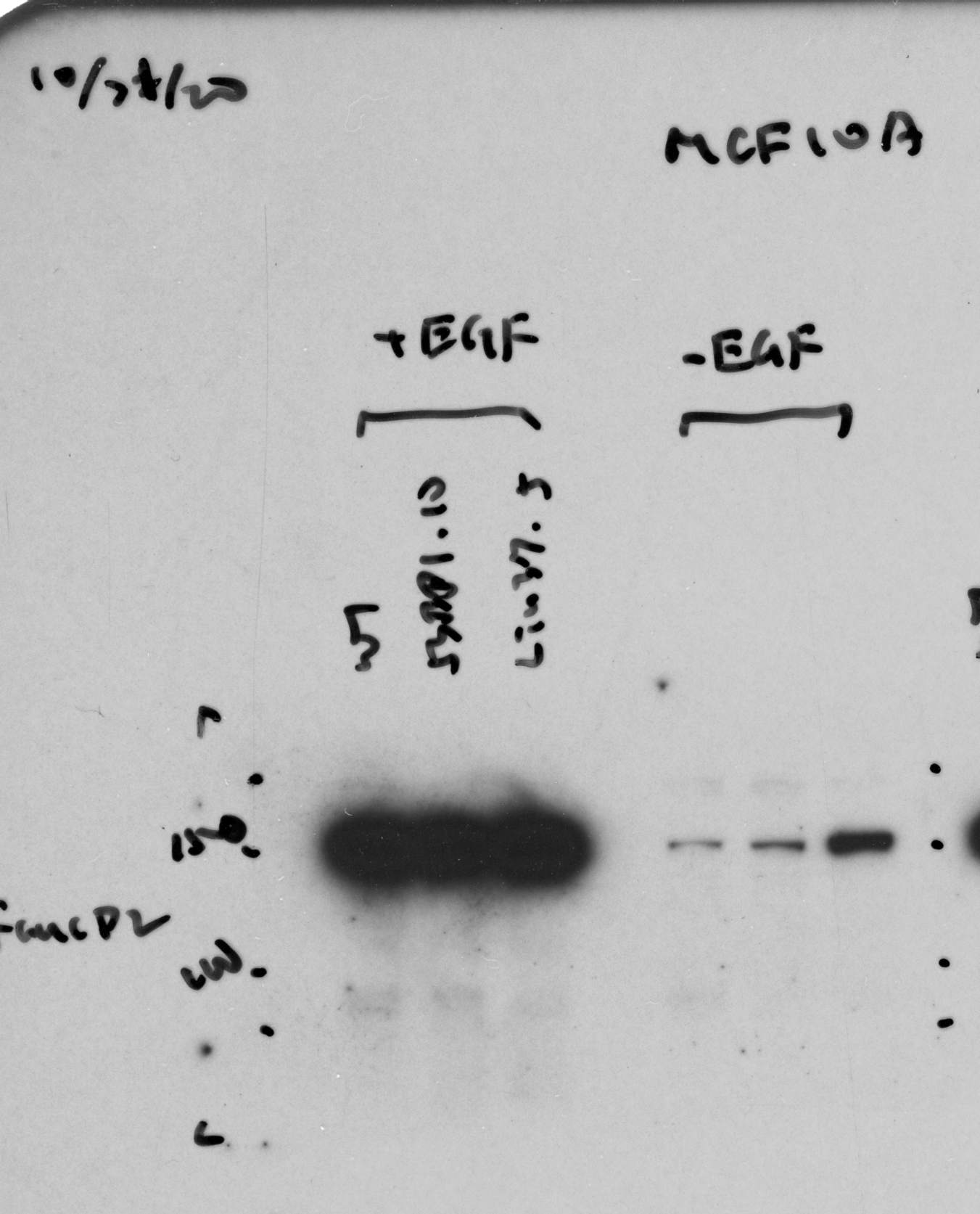

Supplement: Source data 4. [file elife-68466-data4.zip › Source data 4 - figure 5 part 1/Figure 5/102120_FANCD2_Fig 5D.tif]

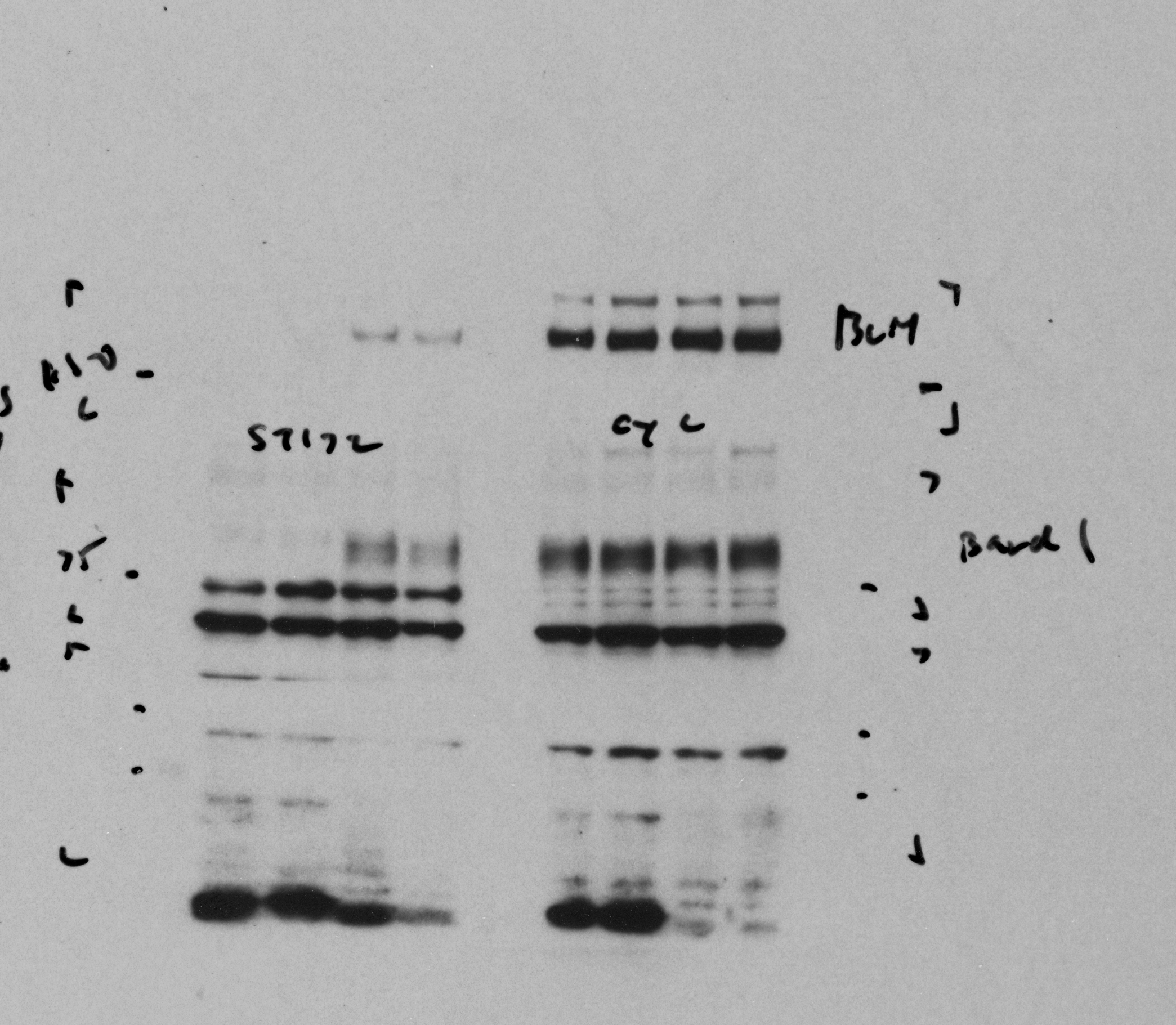

Supplement: Source data 4. [file elife-68466-data4.zip › Source data 4 - figure 5 part 1/Figure 5/031920_BLM-Bard1_Fig 5C.tif]

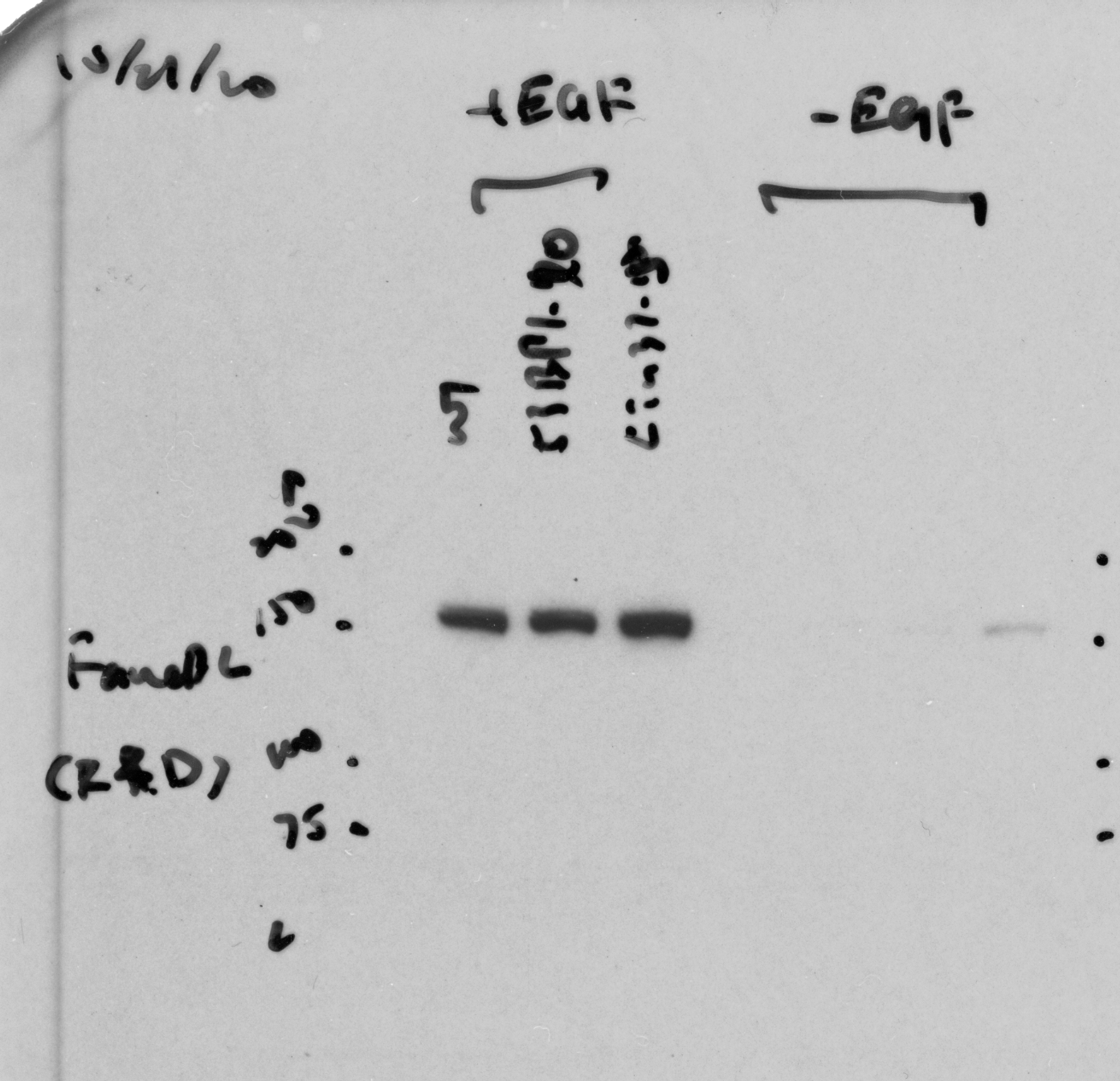

Supplement: Source data 4. [file elife-68466-data4.zip › Source data 4 - figure 5 part 1/Figure 5/102120_FANCD2_Fig 5D0001.tif]

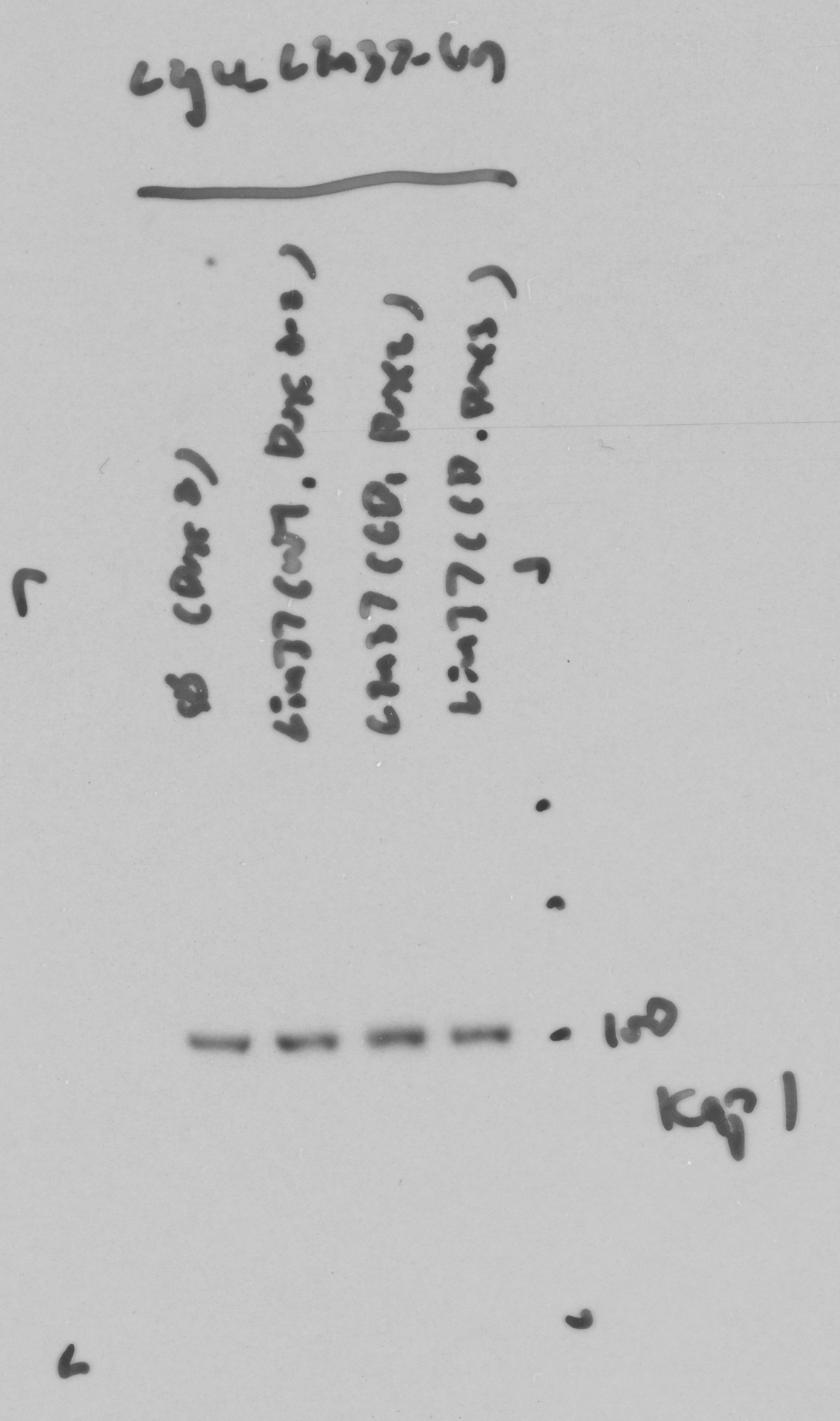

Supplement: Source data 4. [file elife-68466-data4.zip › Source data 4 - figure 5 part 1/Figure 5/0721120_KAP1_Fig 5A.tif]

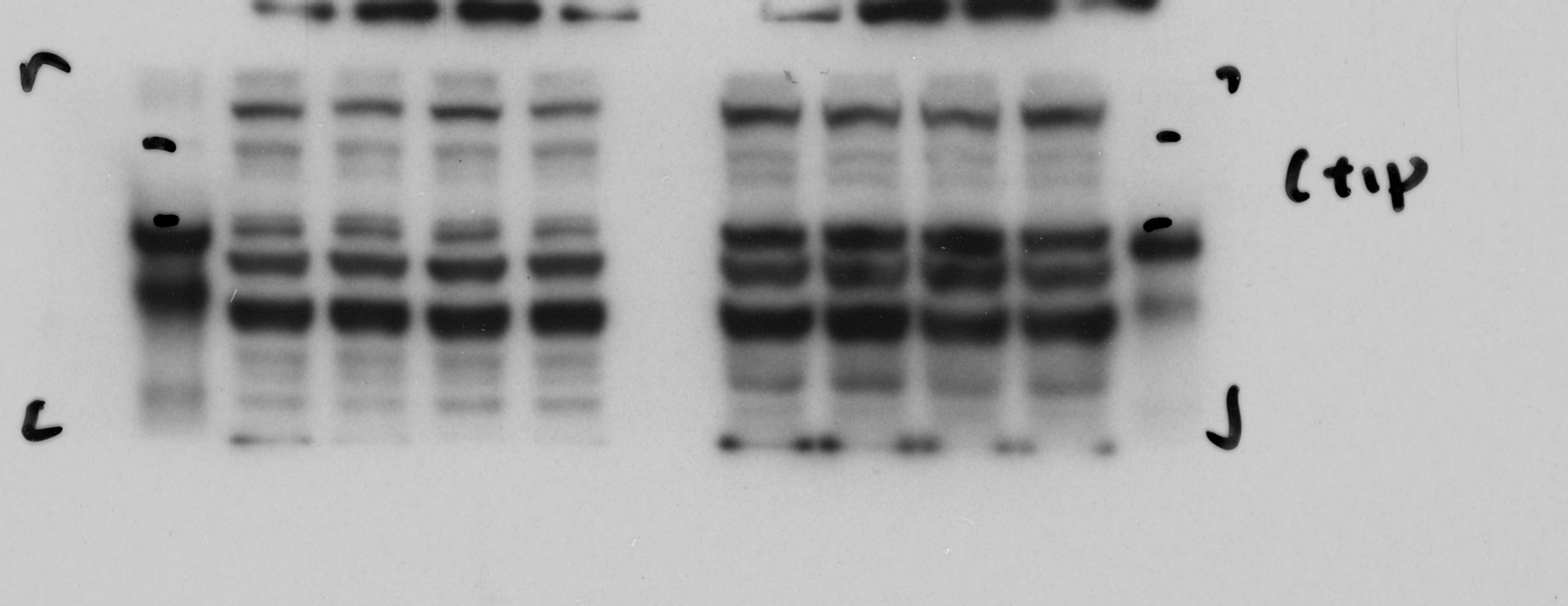

Supplement: Source data 4. [file elife-68466-data4.zip › Source data 4 - figure 5 part 1/Figure 5/060820_CtIP_Fig 5D.tif]

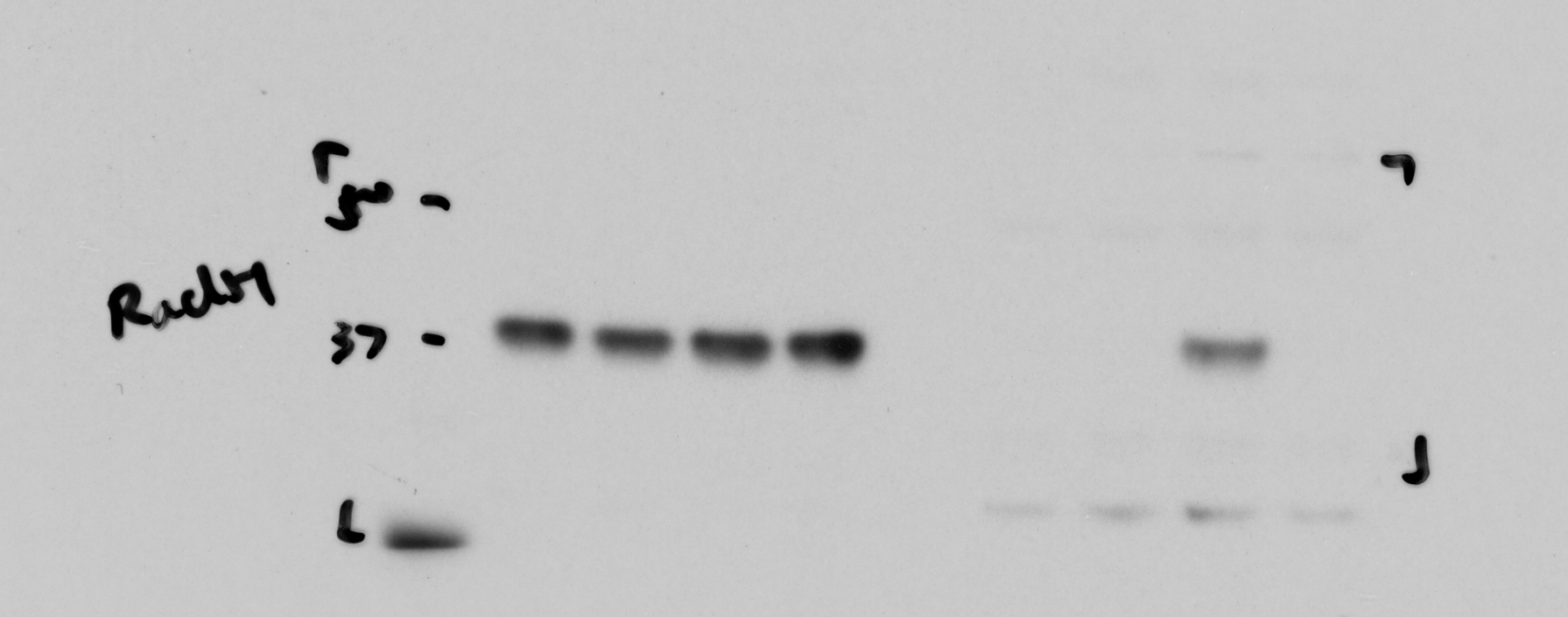

Supplement: Source data 4. [file elife-68466-data4.zip › Source data 4 - figure 5 part 1/Figure 5/060820_RAD51-1_Fig 5D0001.tif]

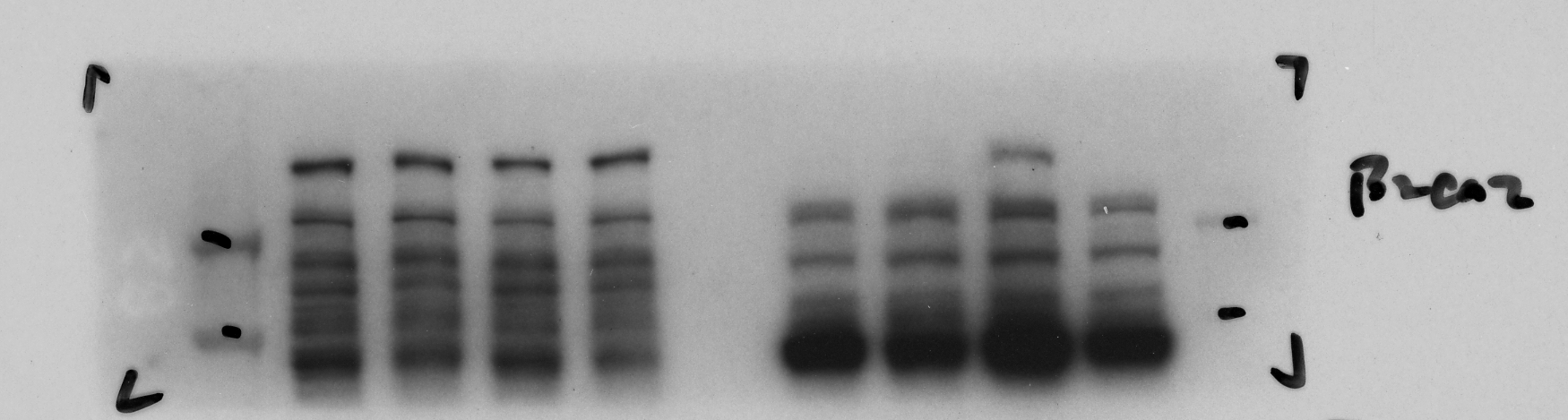

Supplement: Source data 4. [file elife-68466-data4.zip › Source data 4 - figure 5 part 1/Figure 5/0608200003_BRCA2_Fig 5D.tif]

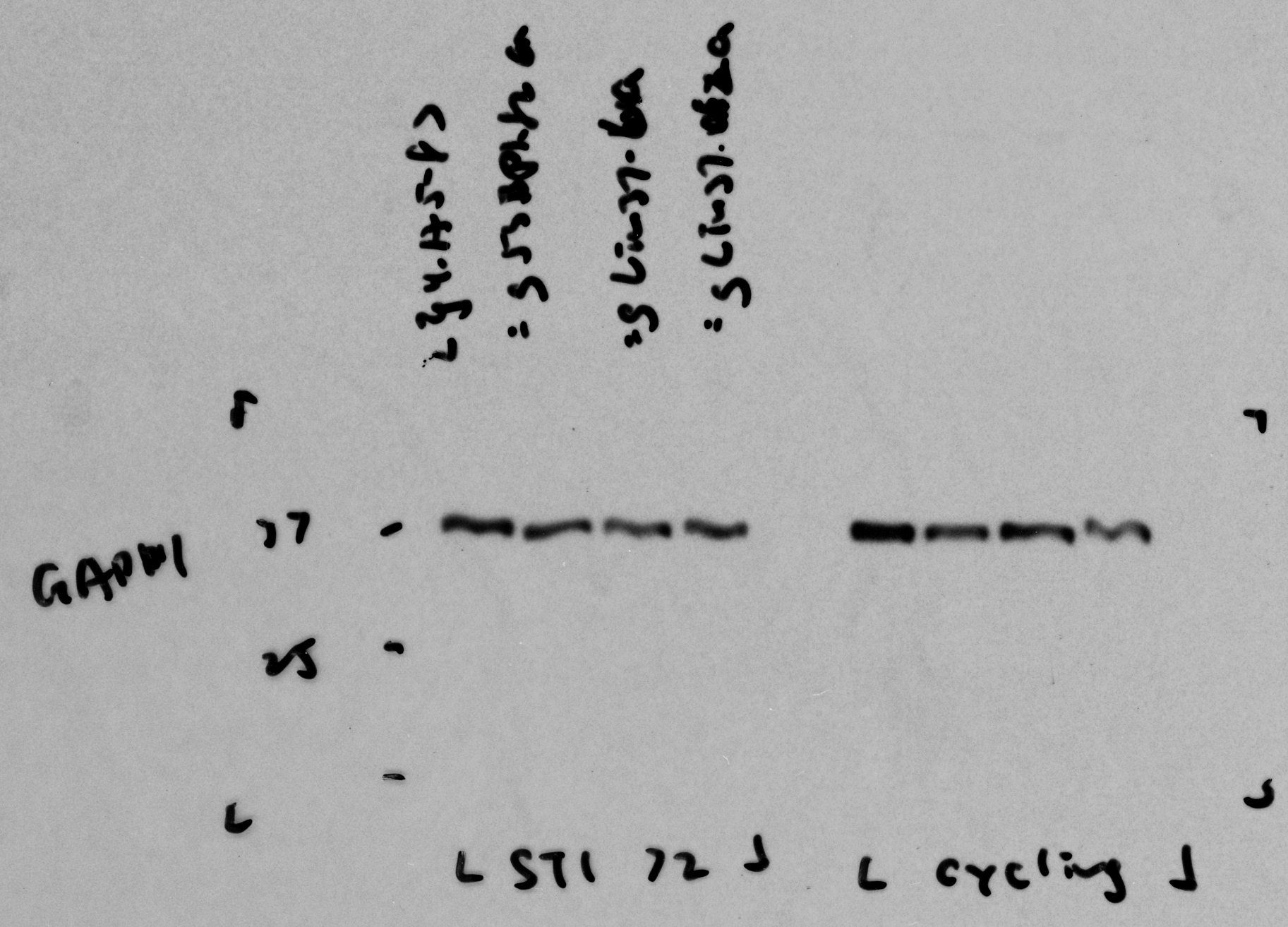

Supplement: Source data 4. [file elife-68466-data4.zip › Source data 4 - figure 5 part 1/Figure 5/032020_6_GAPDH_Fig 5C.tif]

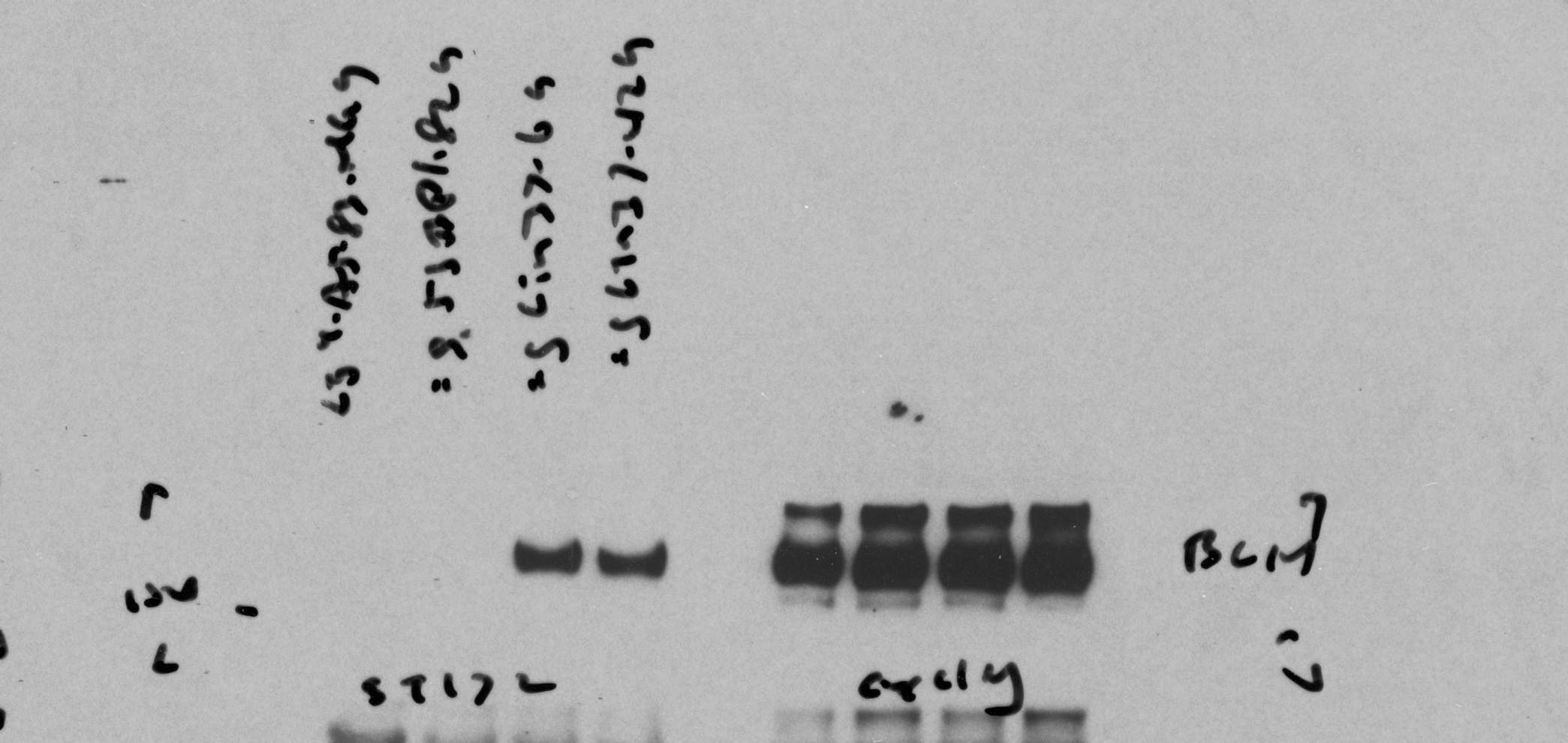

Supplement: Source data 4. [file elife-68466-data4.zip › Source data 4 - figure 5 part 1/Figure 5/031920_BLM_Fig 5C.tif]

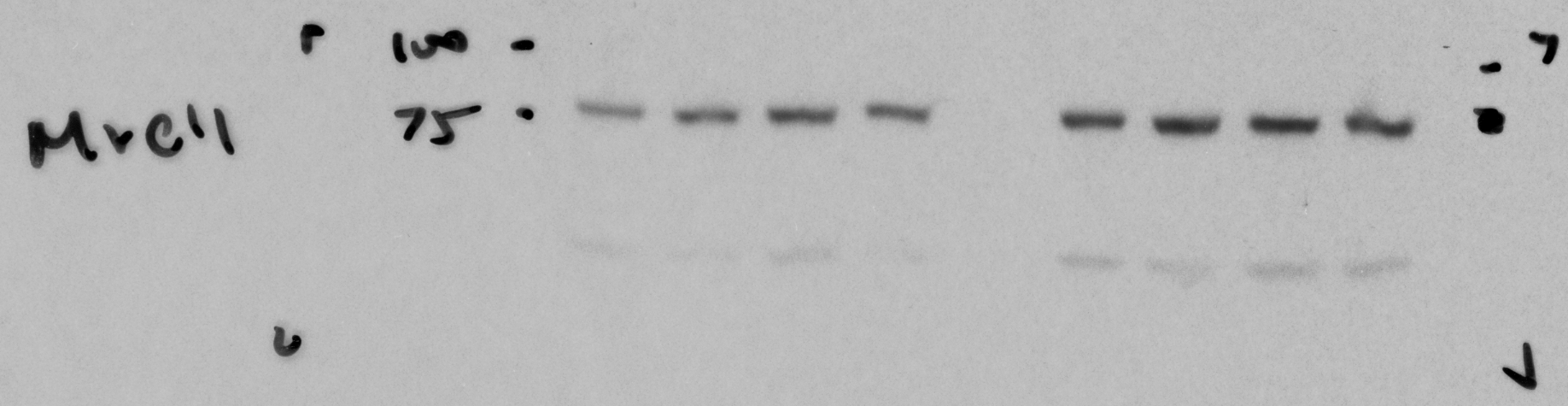

Supplement: Source data 4. [file elife-68466-data4.zip › Source data 4 - figure 5 part 1/Figure 5/032020_6_Mre11_Fig 5C.tif]

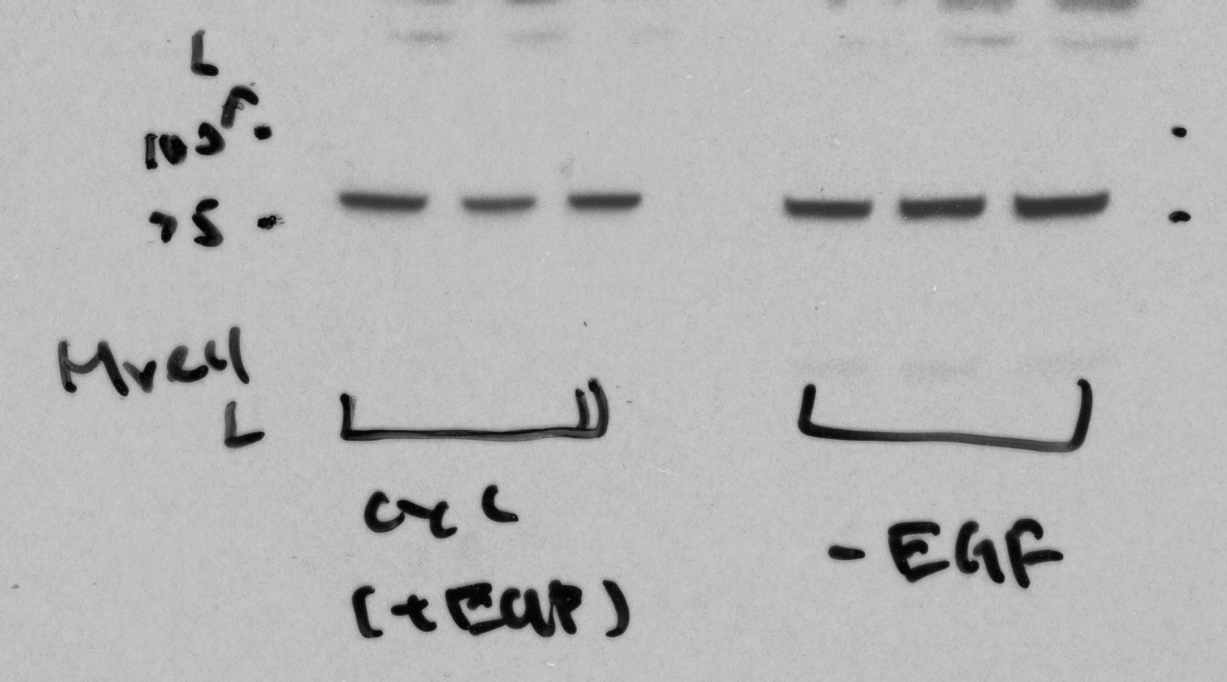

Supplement: Source data 4. [file elife-68466-data4.zip › Source data 4 - figure 5 part 1/Figure 5/051920_Mre11_Fig 5D.tif]

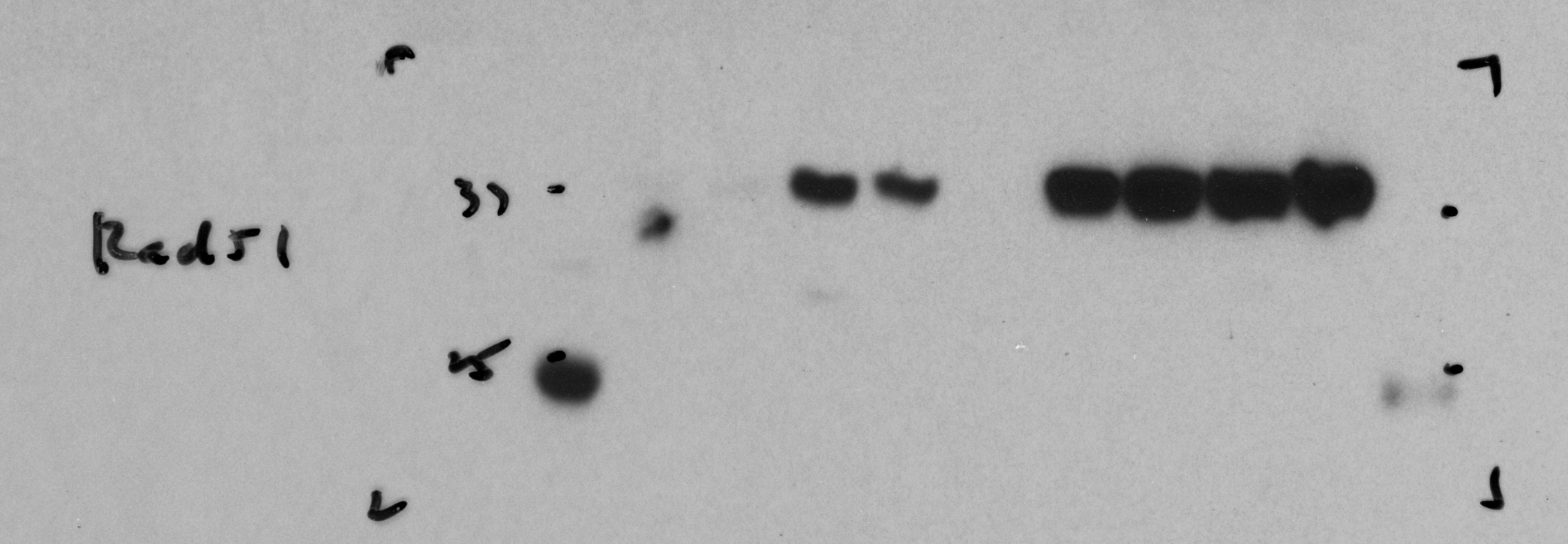

Supplement: Source data 4. [file elife-68466-data4.zip › Source data 4 - figure 5 part 1/Figure 5/032020_5_RAD51_Fig 5C.tif]

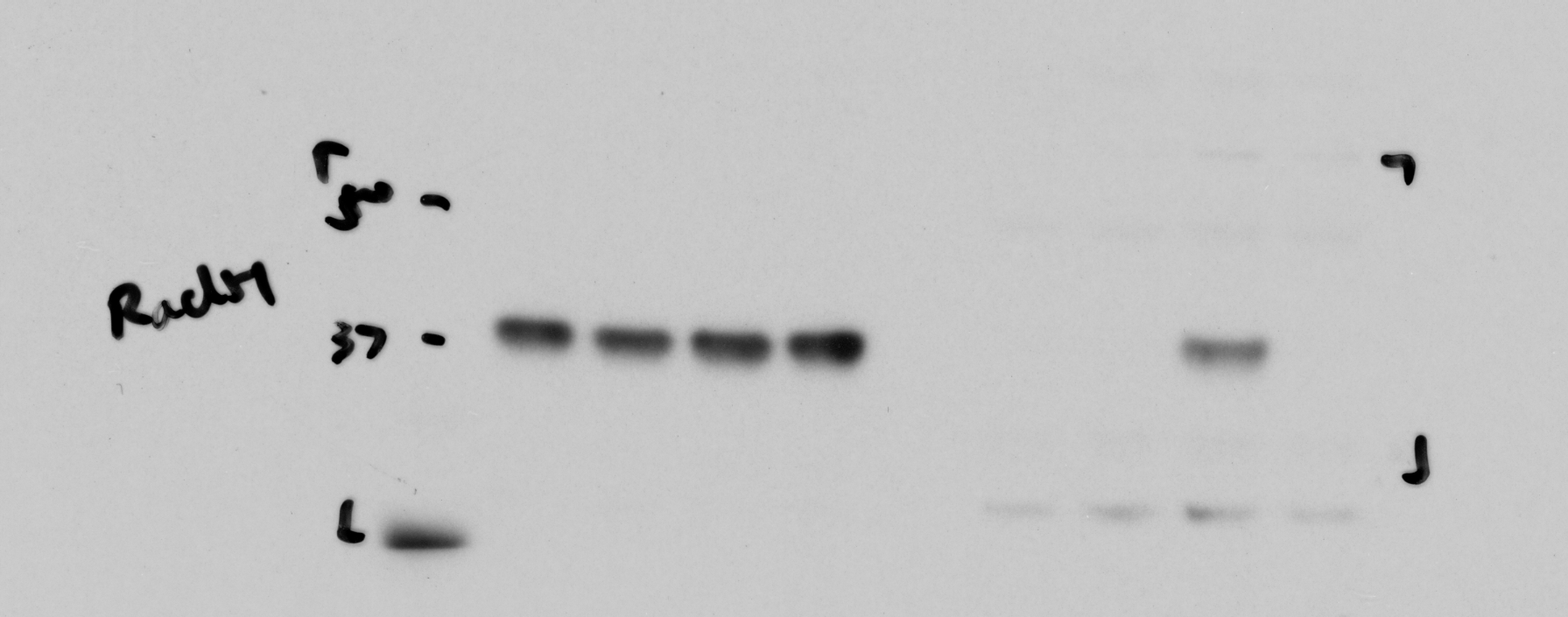

Supplement: Source data 4. [file elife-68466-data4.zip › Source data 4 - figure 5 part 1/Figure 5/060820_RAD51-1_Fig 5D.tif]

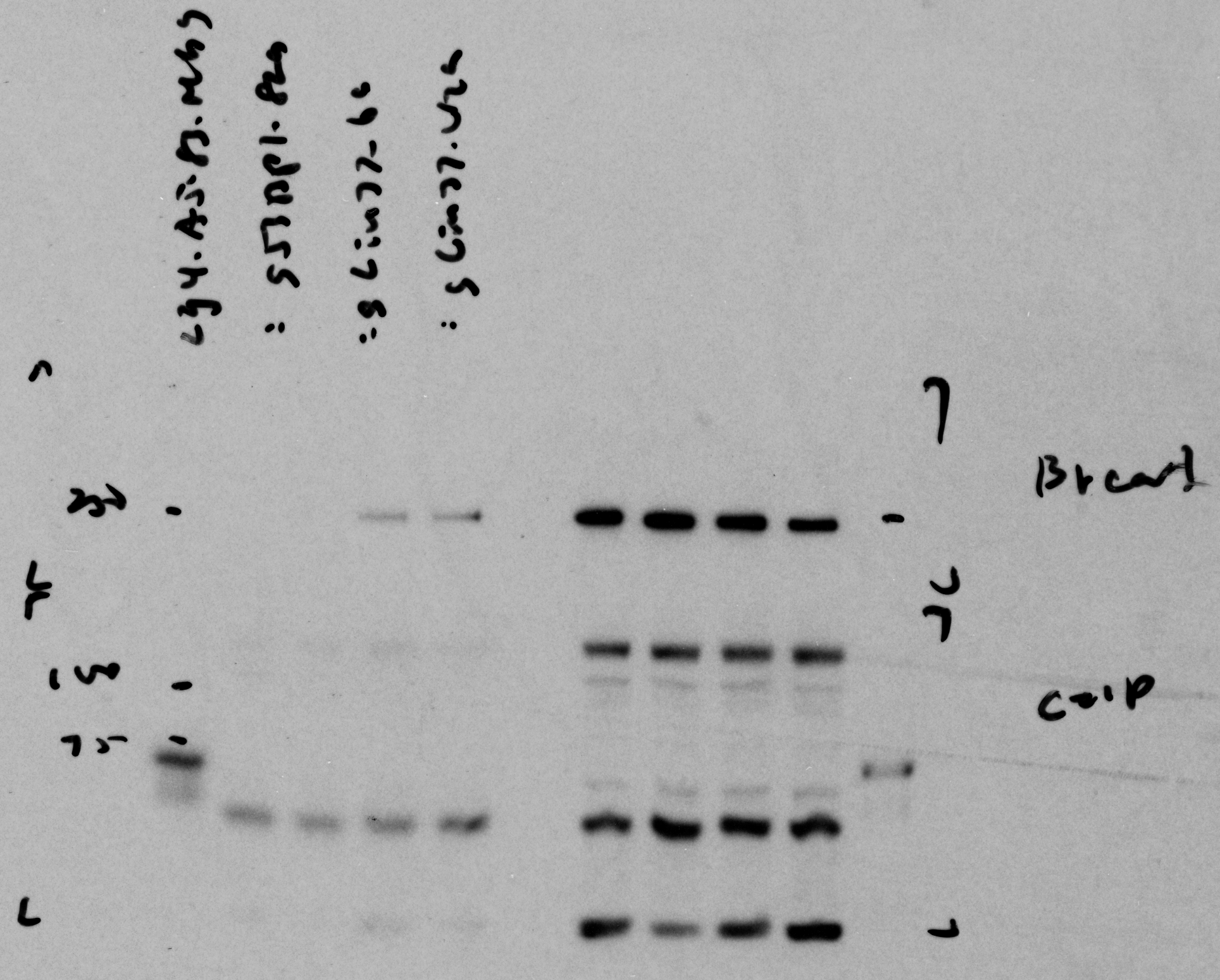

Supplement: Source data 4. [file elife-68466-data4.zip › Source data 4 - figure 5 part 1/Figure 5/032020_6_CtIP_Fig 5C.tif]

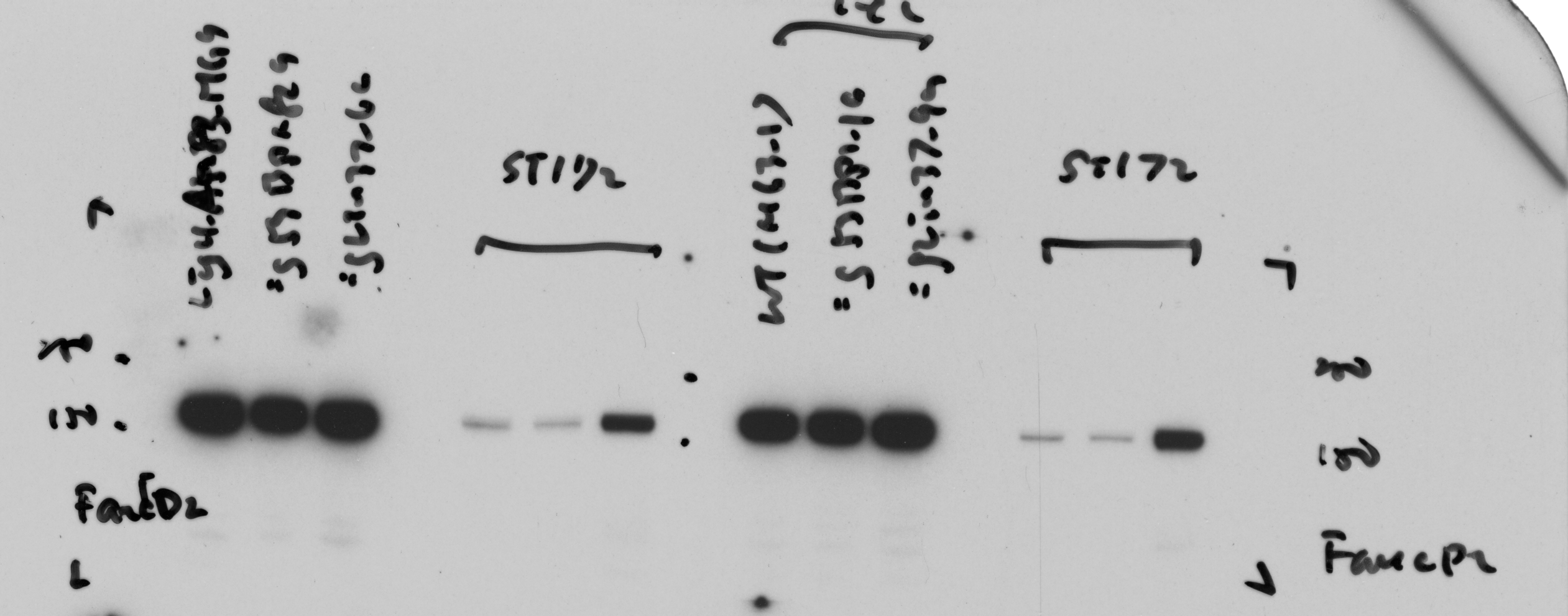

Supplement: Source data 4. [file elife-68466-data4.zip › Source data 4 - figure 5 part 1/Figure 5/083120_FANCD2_Fig 5C0001.tif]

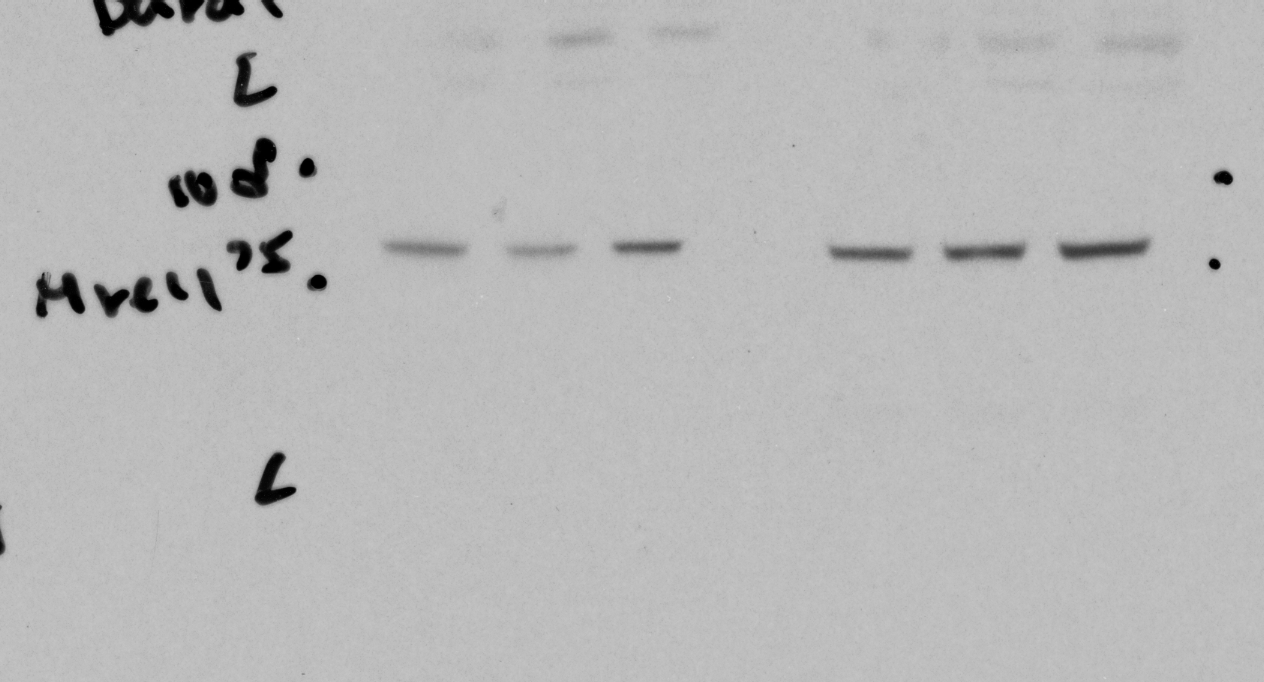

Supplement: Source data 4. [file elife-68466-data4.zip › Source data 4 - figure 5 part 1/Figure 5/051920_Mre11_Fig 5D0001.tif]

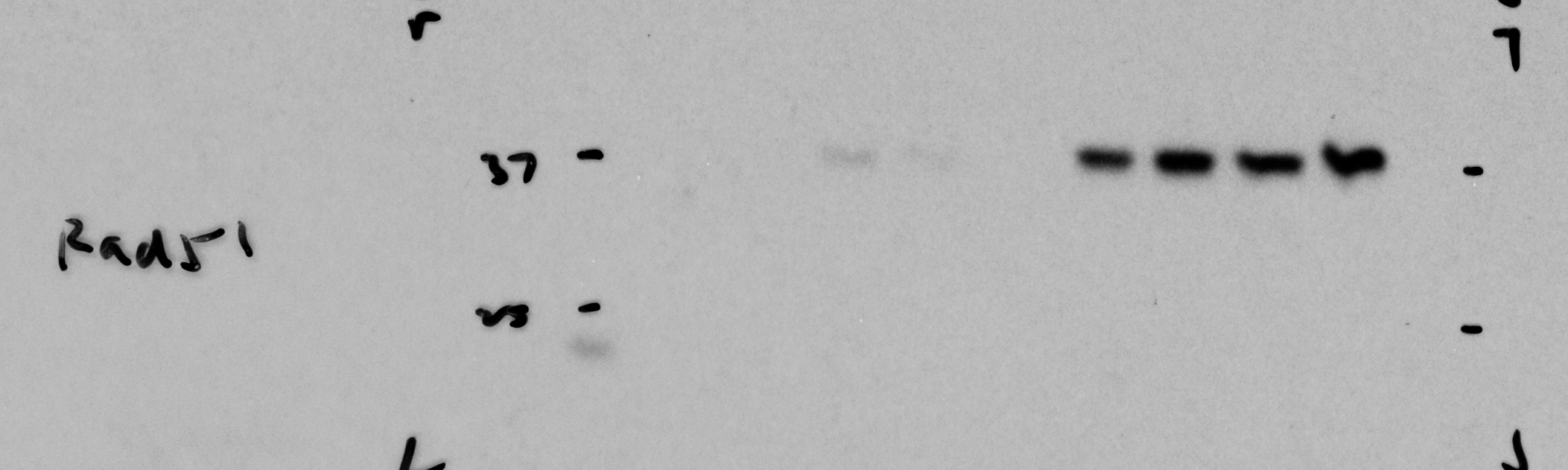

Supplement: Source data 4. [file elife-68466-data4.zip › Source data 4 - figure 5 part 1/Figure 5/032020_7_RAD51_Fig 5C copy.tif]

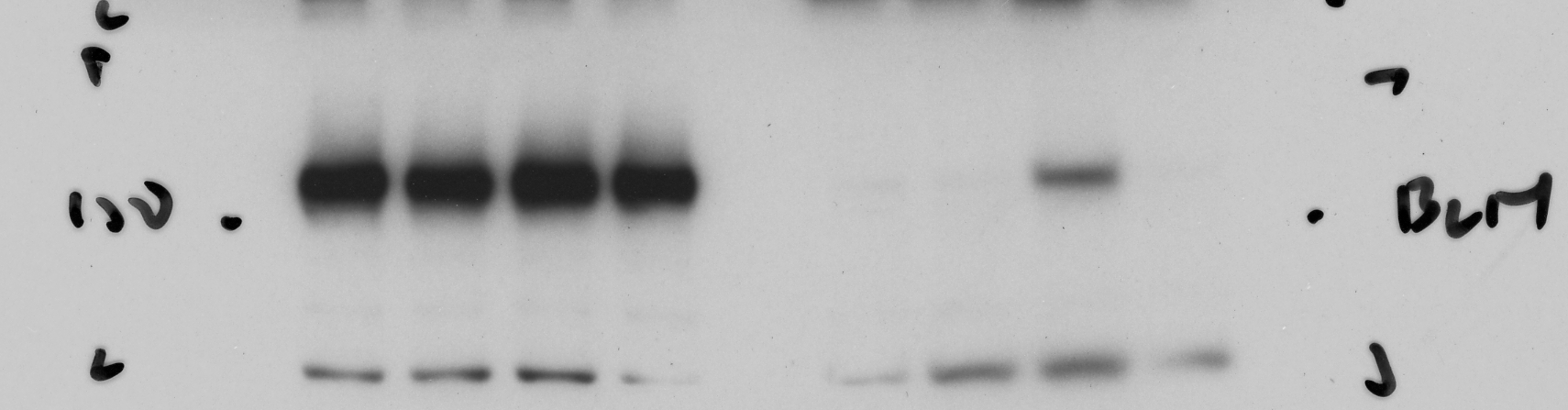

Supplement: Source data 4. [file elife-68466-data4.zip › Source data 4 - figure 5 part 1/Figure 5/0608200002_BLM_Fig 5D.tif]

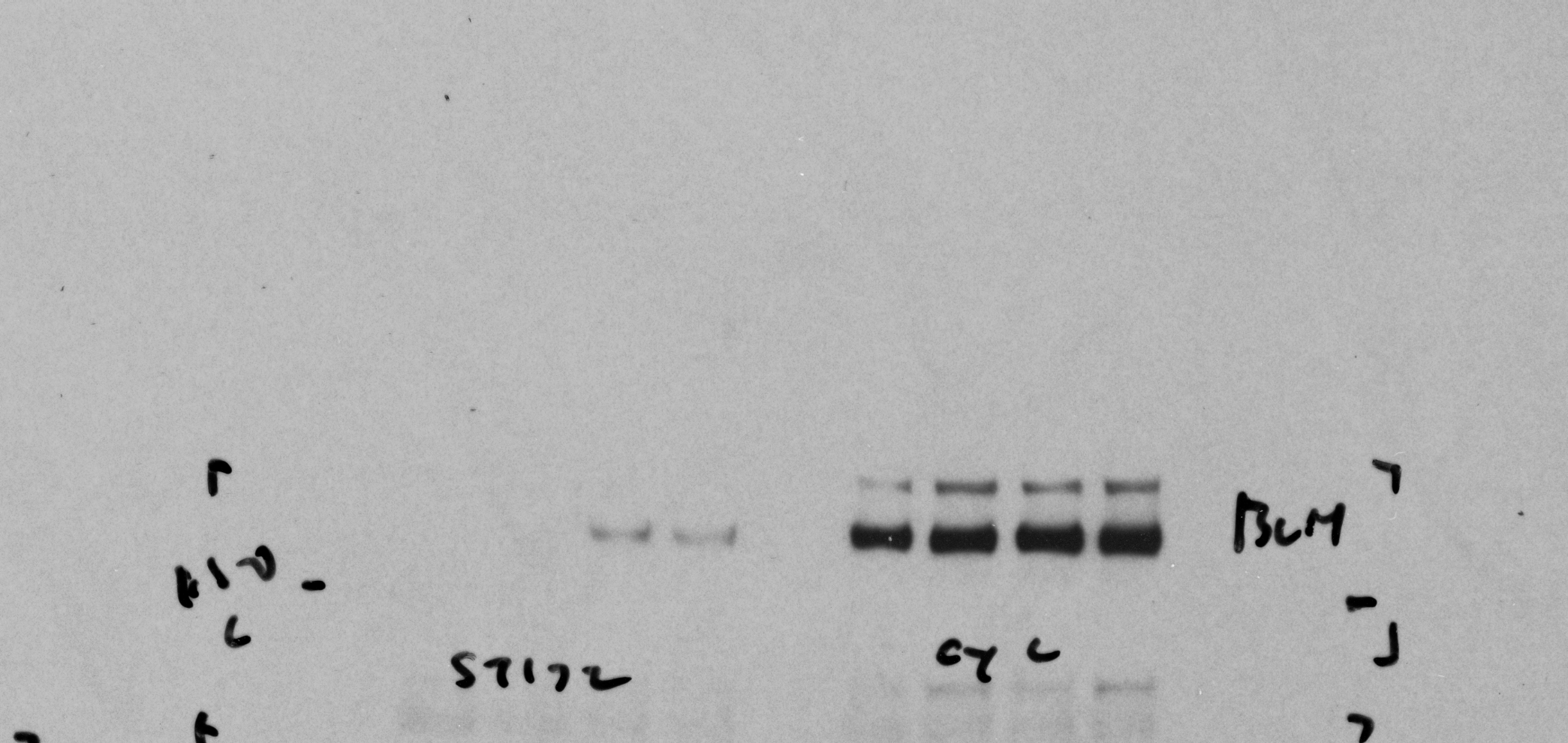

Supplement: Source data 4. [file elife-68466-data4.zip › Source data 4 - figure 5 part 1/Figure 5/031920_BLM_Fig 5C0001.tif]

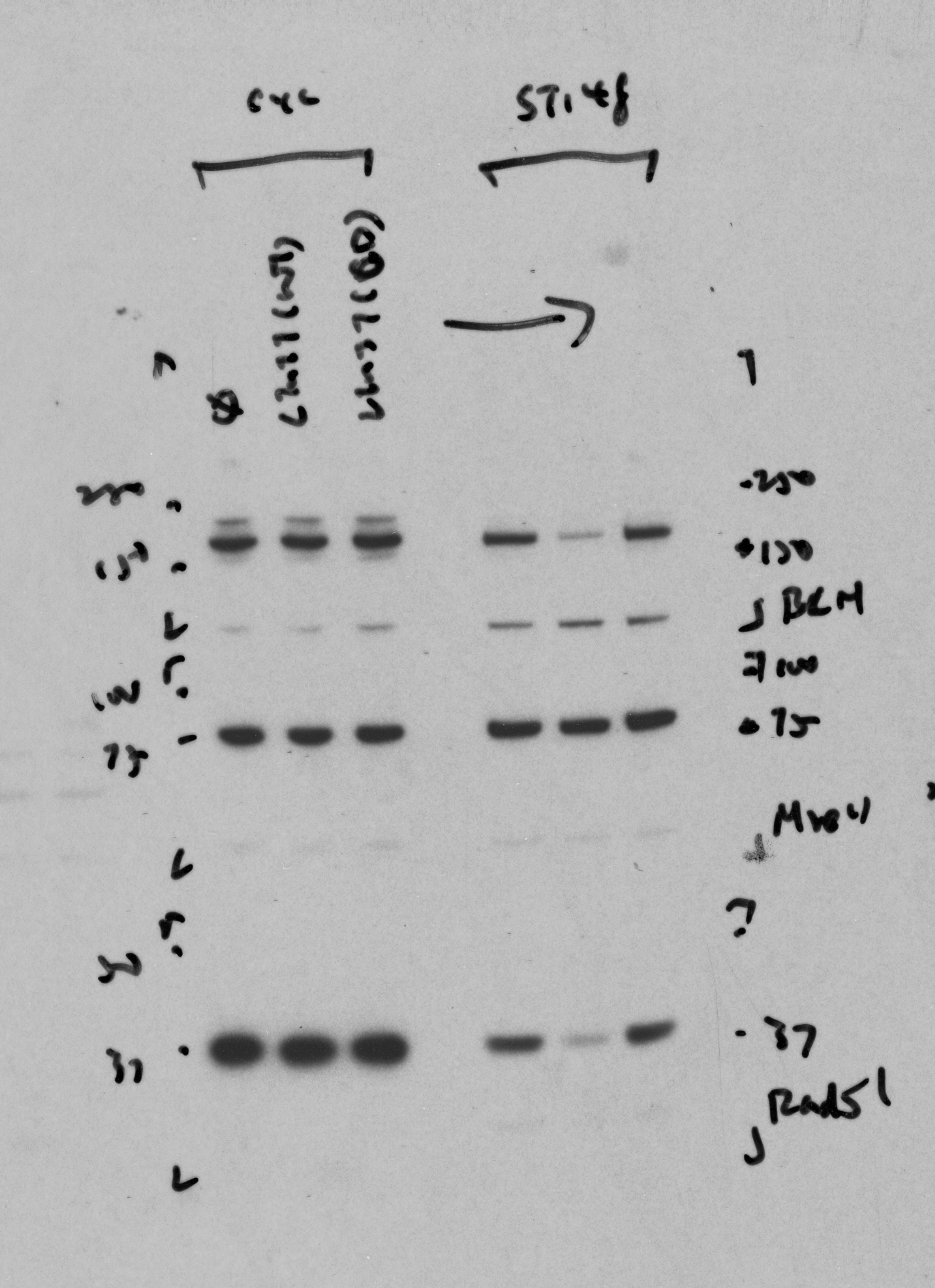

Supplement: Source data 5. [file elife-68466-data5.zip › Source data 5 - figure 5 part 2/Figure 5 S Source data/051120_BLM_Fig 5E.tif]

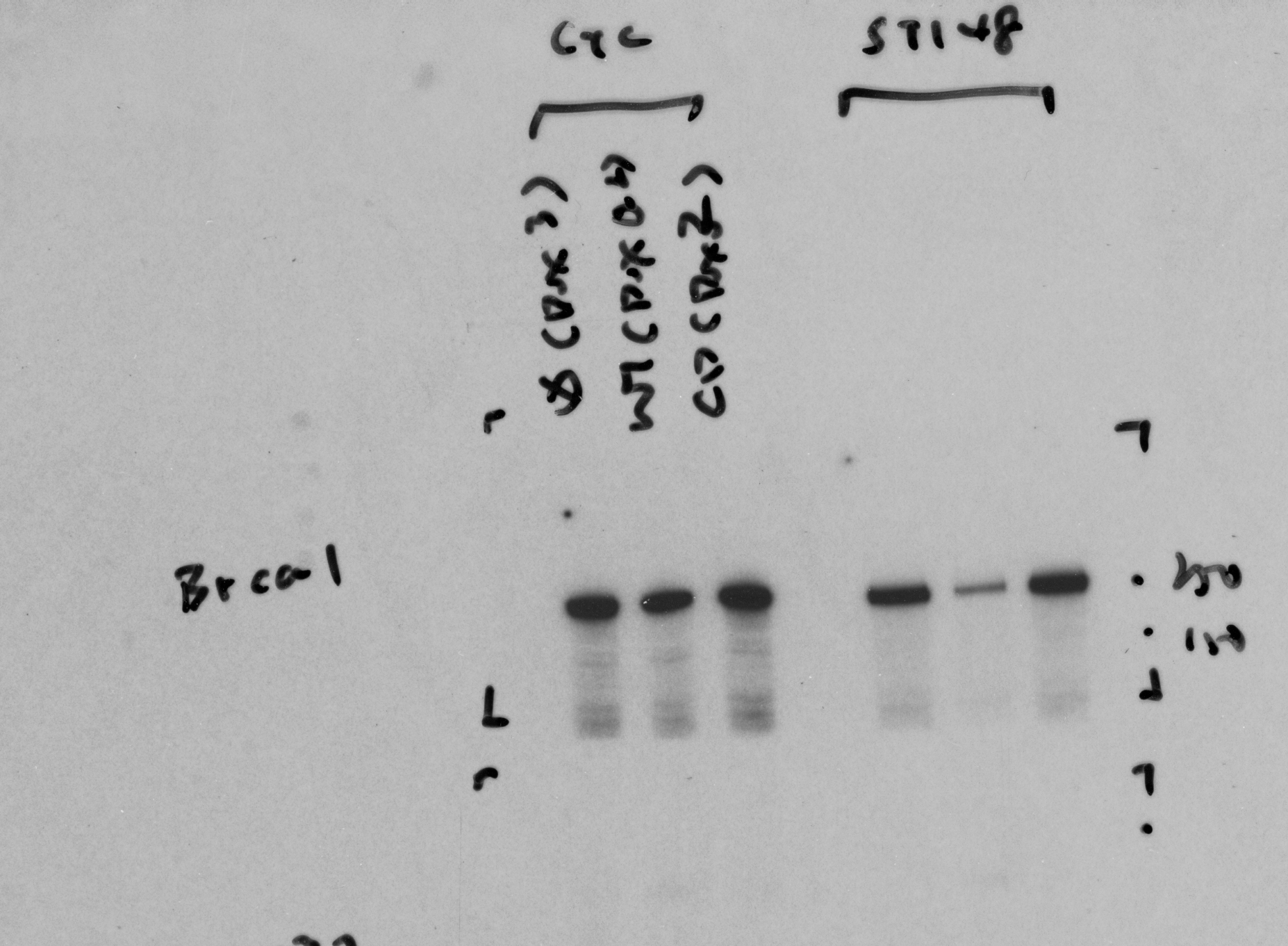

Supplement: Source data 5. [file elife-68466-data5.zip › Source data 5 - figure 5 part 2/Figure 5 S Source data/051120_BRCA1_Fig 5E0001.tif]

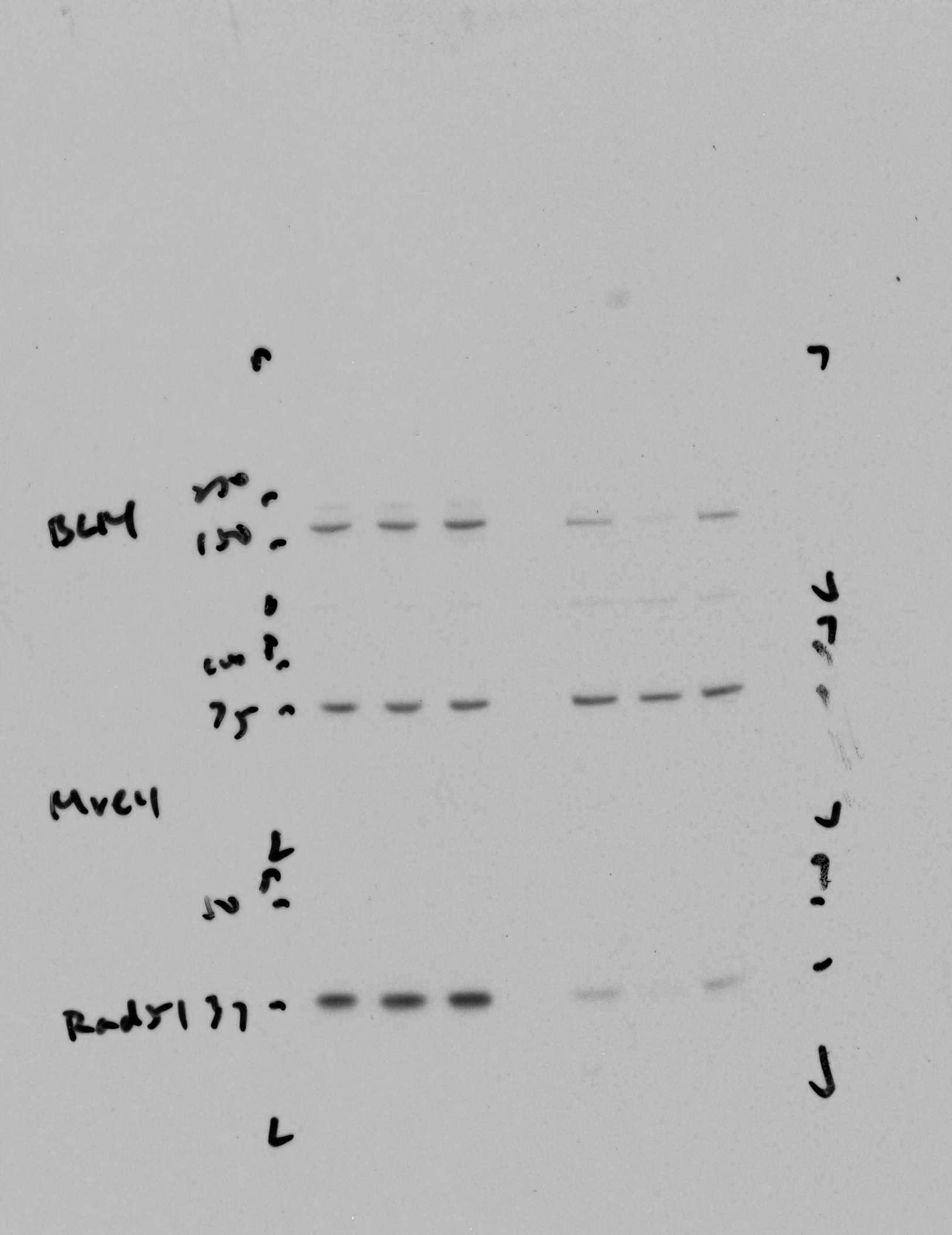

Supplement: Source data 5. [file elife-68466-data5.zip › Source data 5 - figure 5 part 2/Figure 5 S Source data/051120_Rad51_Fig 5E.tif]

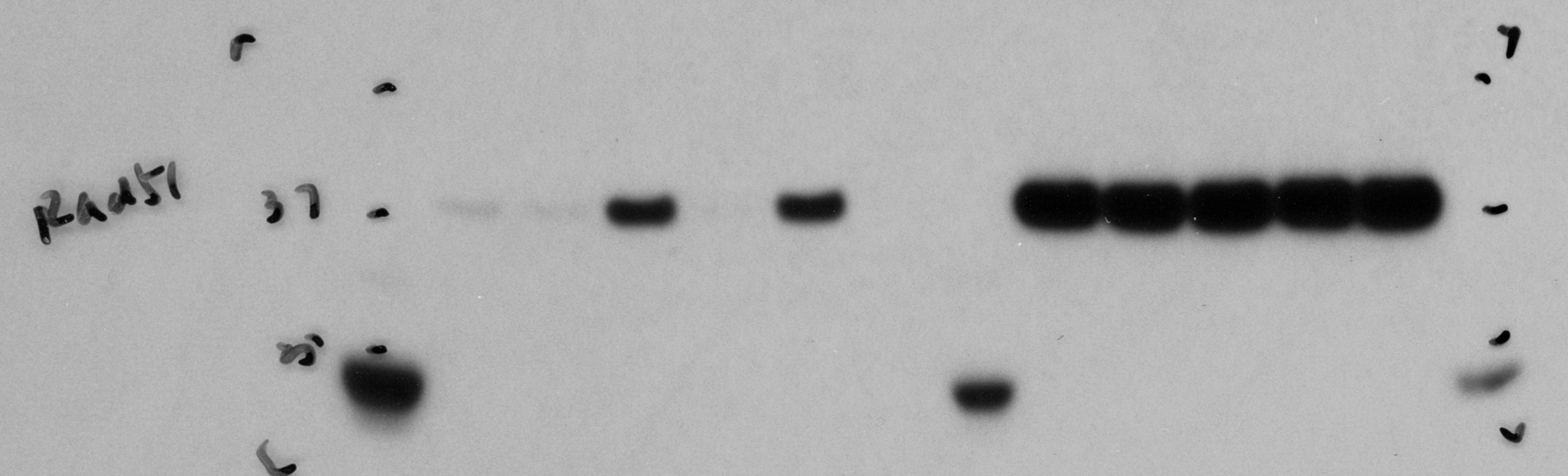

Supplement: Source data 5. [file elife-68466-data5.zip › Source data 5 - figure 5 part 2/Figure 5 S Source data/031220_9_RAD51_Fig 5 S1B.tif]

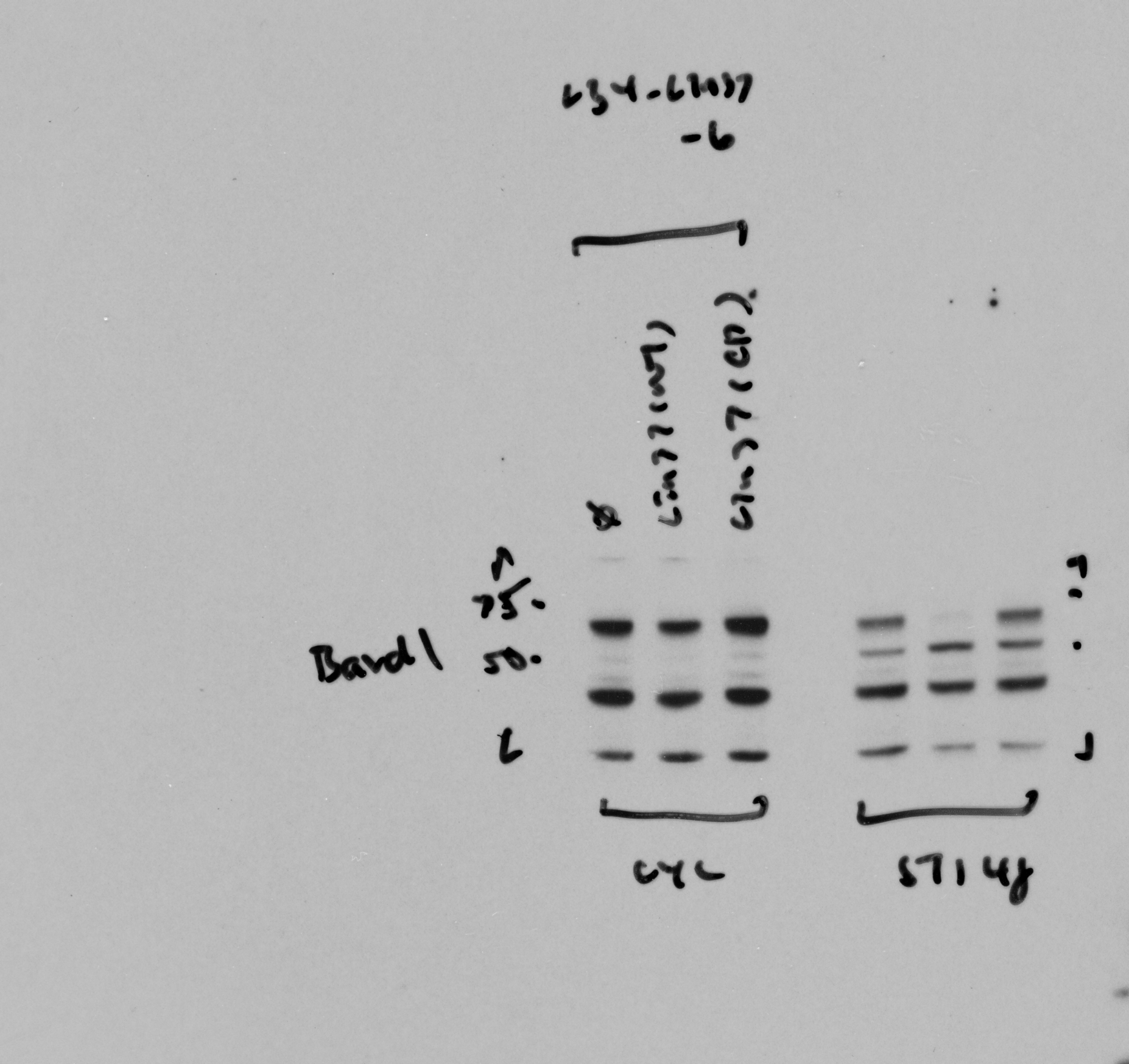

Supplement: Source data 5. [file elife-68466-data5.zip › Source data 5 - figure 5 part 2/Figure 5 S Source data/051120_BRCA1_Fig 5E0002.tif]

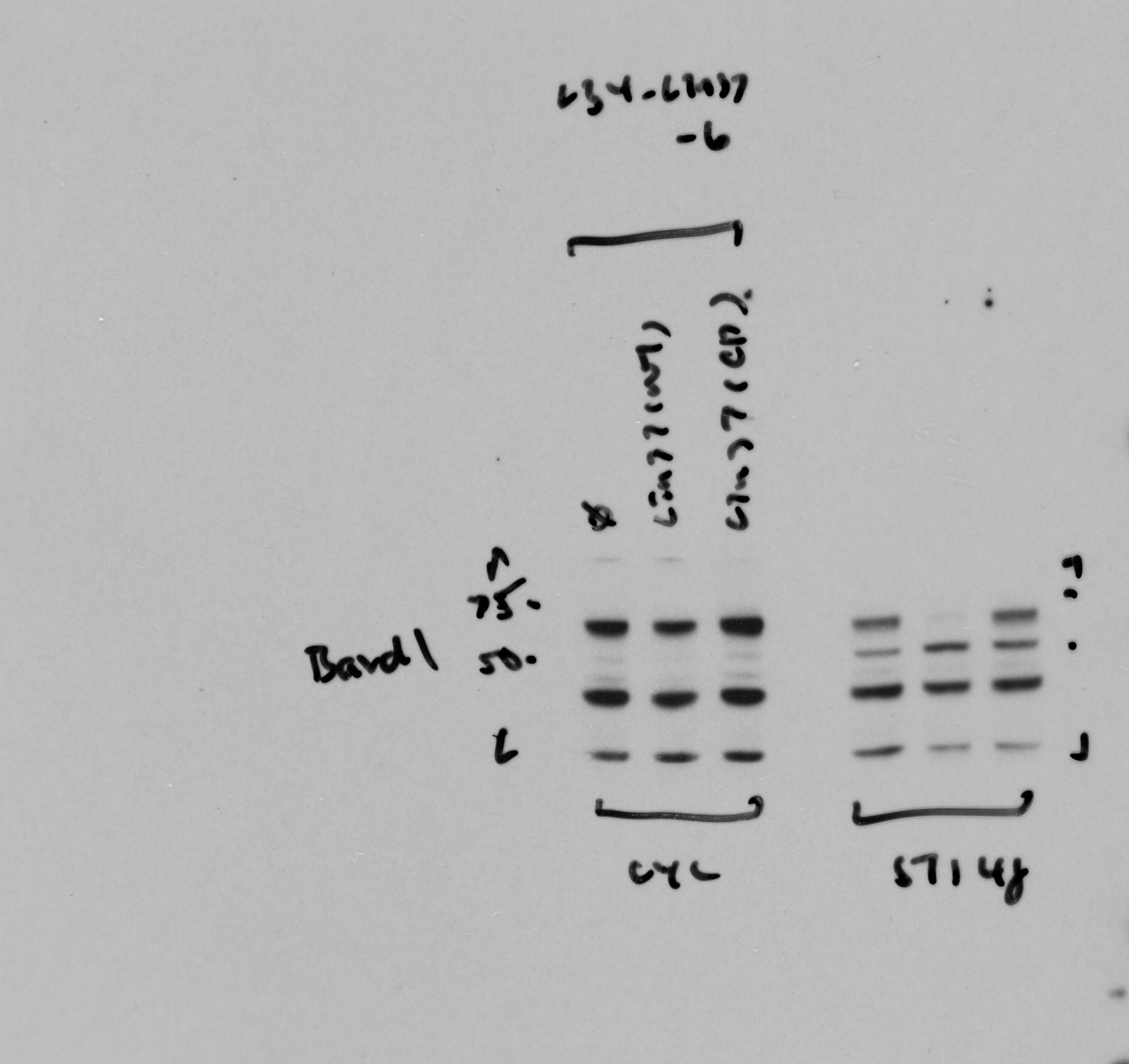

Supplement: Source data 5. [file elife-68466-data5.zip › Source data 5 - figure 5 part 2/Figure 5 S Source data/051120_Bard1_Fig 5E.tif]

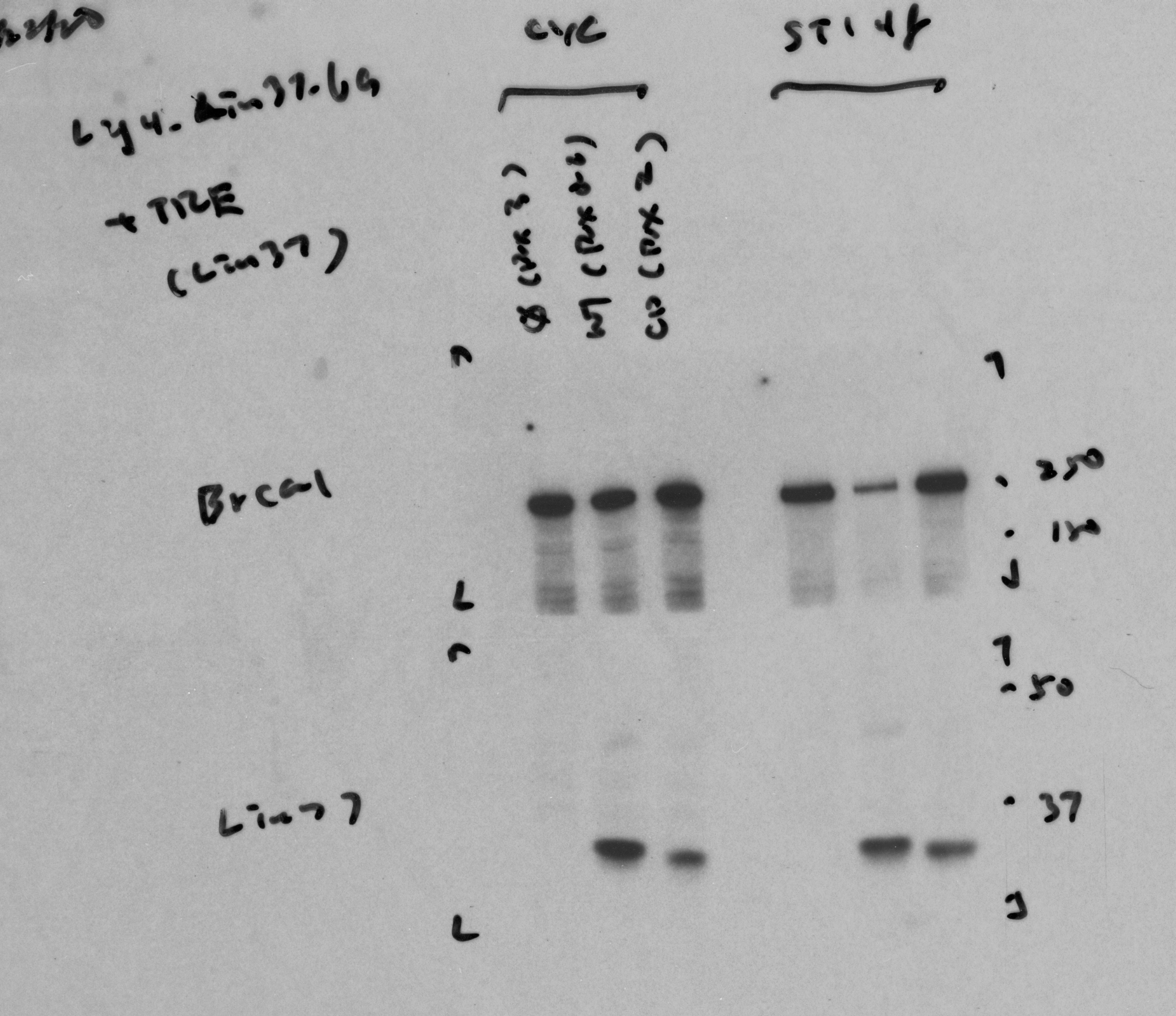

Supplement: Source data 5. [file elife-68466-data5.zip › Source data 5 - figure 5 part 2/Figure 5 S Source data/051120_Brca1+Lin371_Fig 5E.tif]

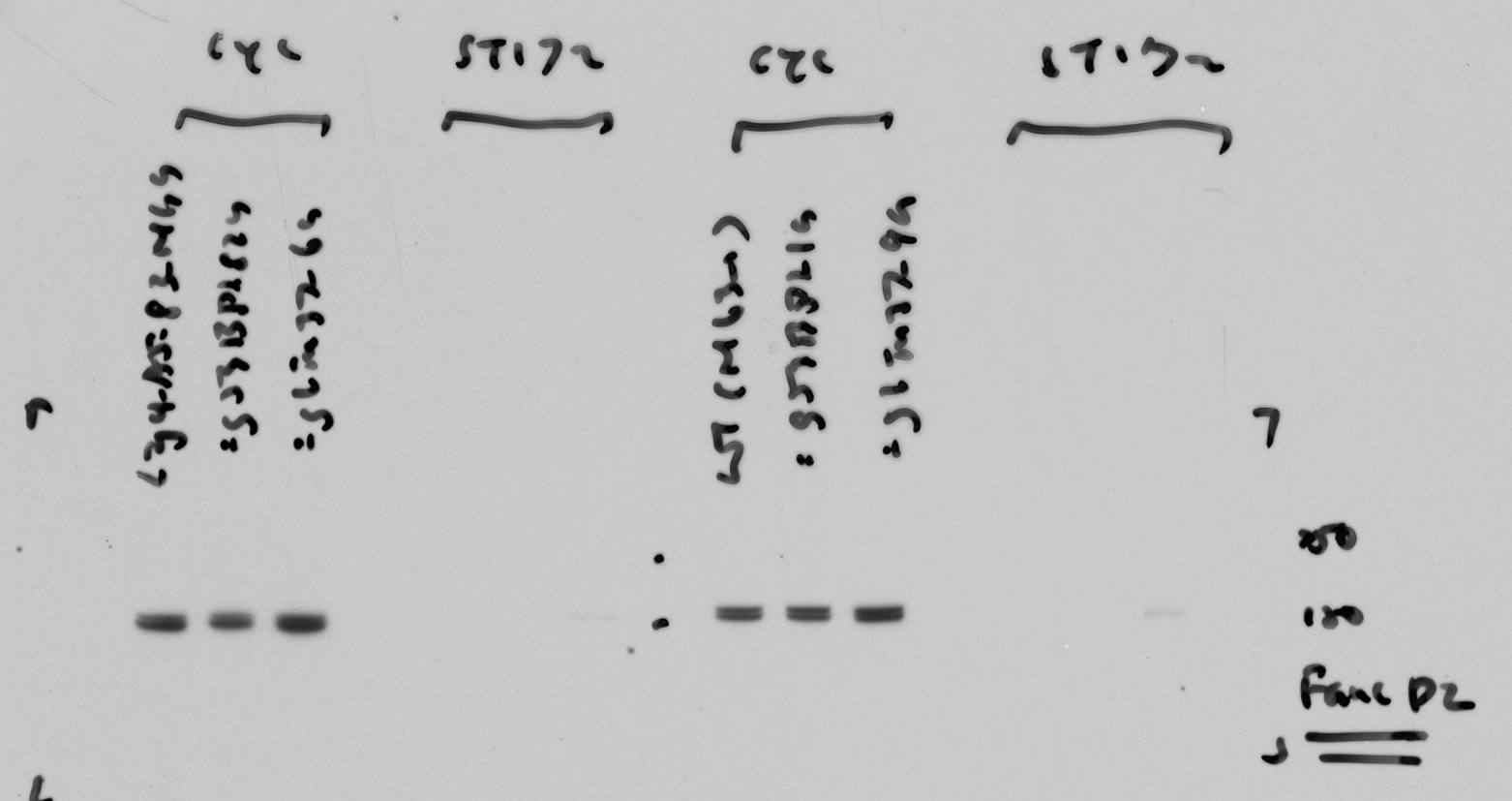

Supplement: Source data 5. [file elife-68466-data5.zip › Source data 5 - figure 5 part 2/Figure 5 S Source data/0831200004_FANCD2_Fig 5 S1B.tif]

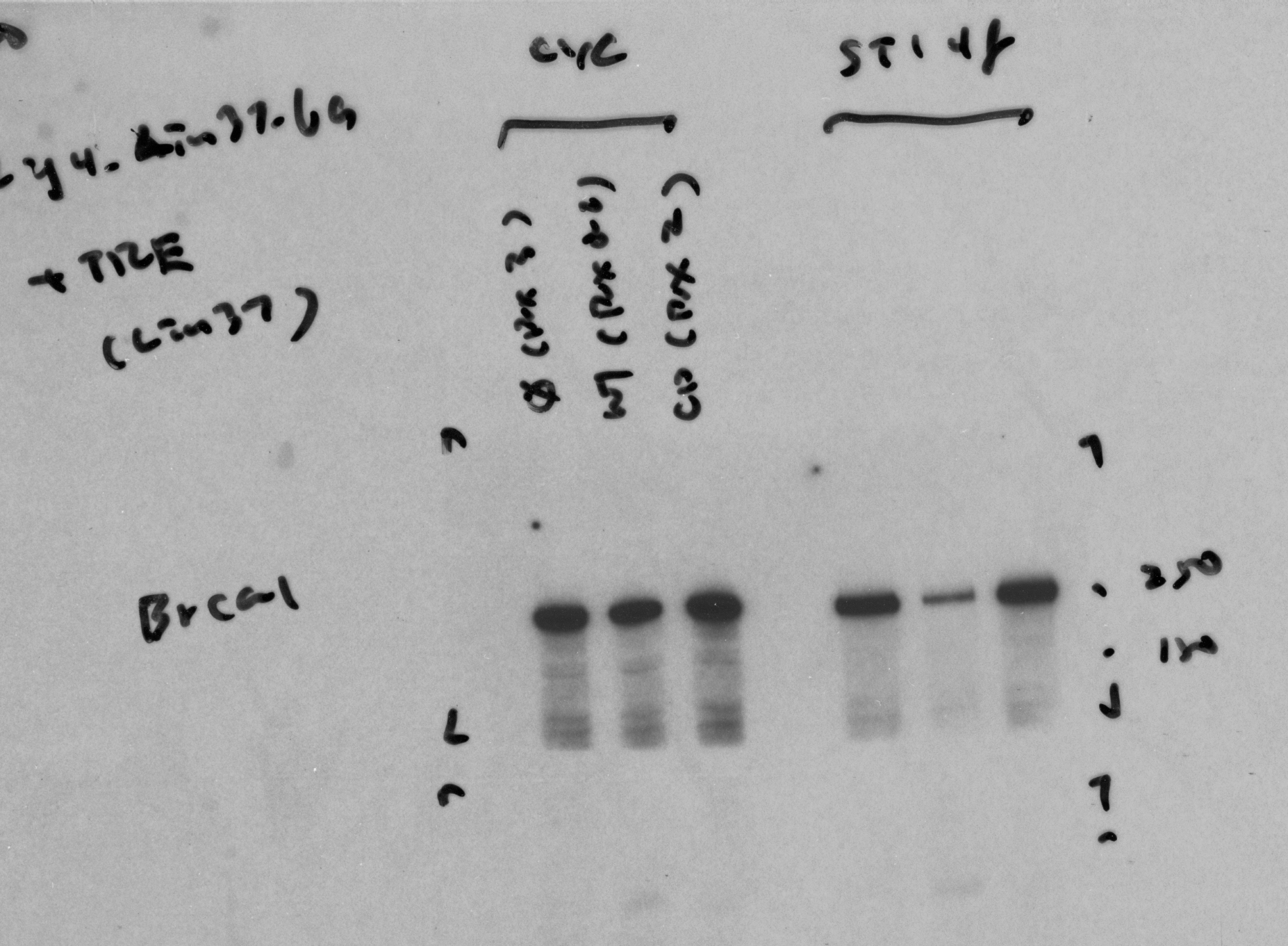

Supplement: Source data 5. [file elife-68466-data5.zip › Source data 5 - figure 5 part 2/Figure 5 S Source data/051120_BRCA1_Fig 5E.tif]

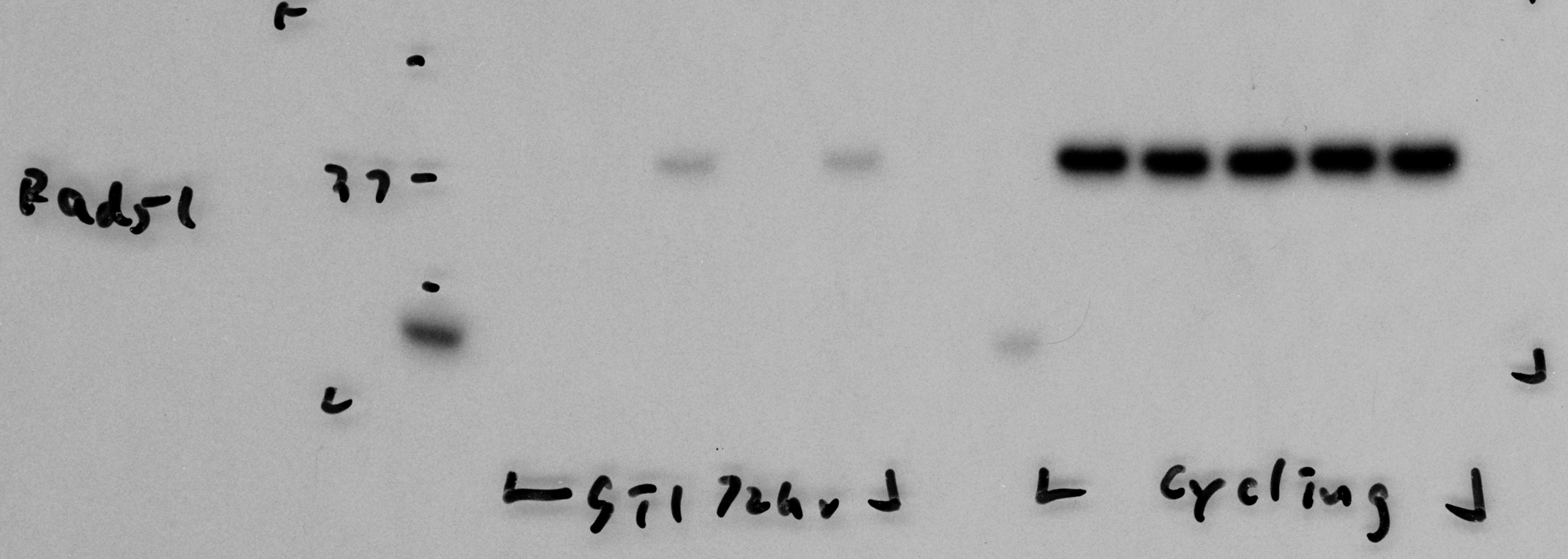

Supplement: Source data 5. [file elife-68466-data5.zip › Source data 5 - figure 5 part 2/Figure 5 S Source data/031220_1_RAD51_Fig 5 S1B.tif]

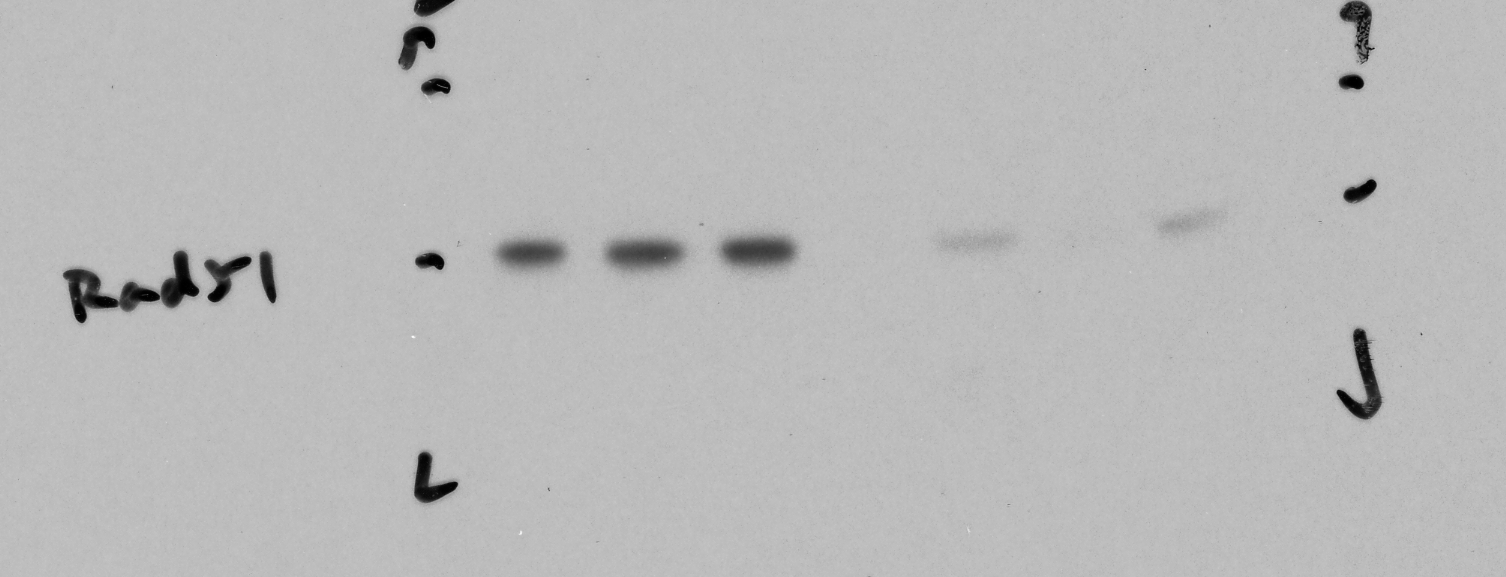

Supplement: Source data 5. [file elife-68466-data5.zip › Source data 5 - figure 5 part 2/Figure 5 S Source data/052120-3_RAD51_Fig 5E.tif]

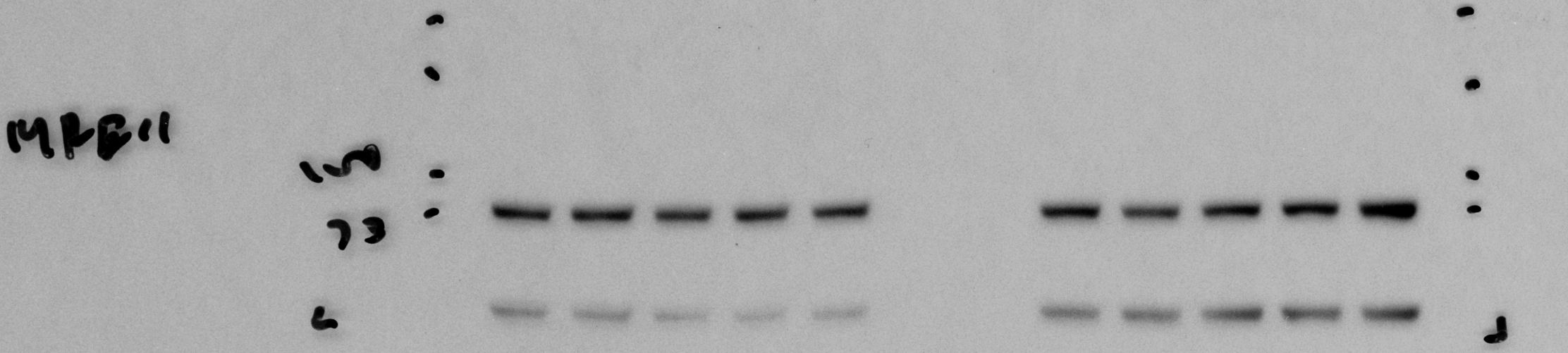

Supplement: Source data 5. [file elife-68466-data5.zip › Source data 5 - figure 5 part 2/Figure 5 S Source data/031220_1_MRE11_Fig 5 S1B.tif]

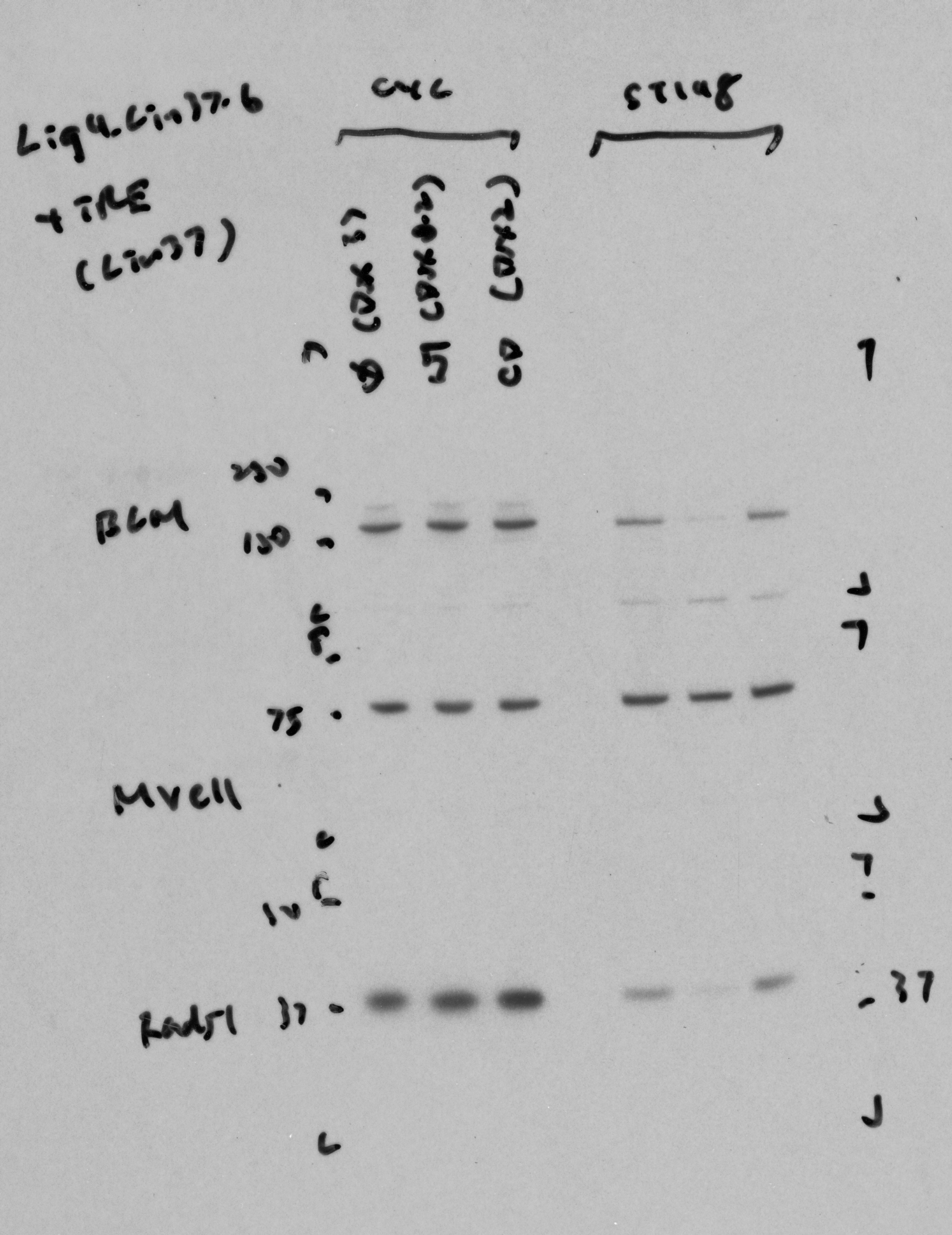

Supplement: Source data 5. [file elife-68466-data5.zip › Source data 5 - figure 5 part 2/Figure 5 S Source data/051120_Mre11_Fig 5E0001.tif]

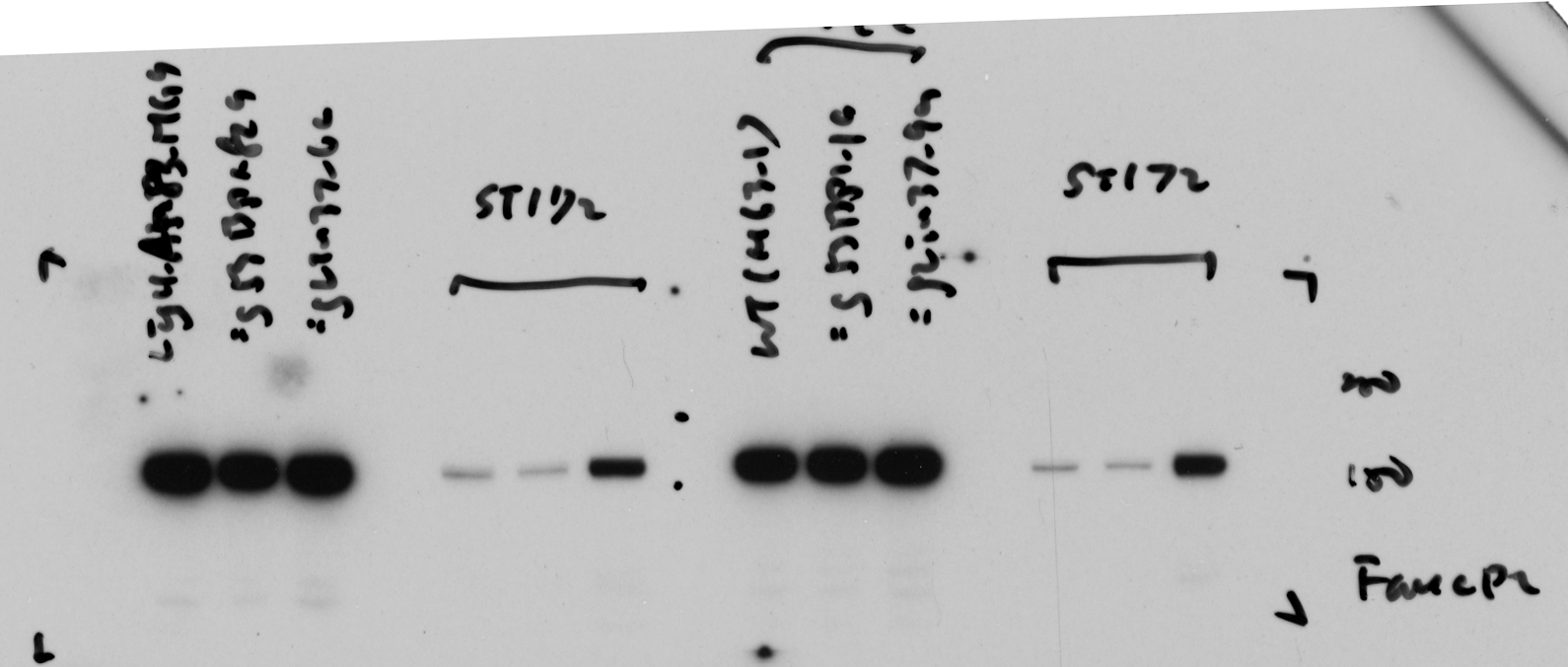

Supplement: Source data 5. [file elife-68466-data5.zip › Source data 5 - figure 5 part 2/Figure 5 S Source data/0831200007_FANCD2_Fig 5 S1B.tif]

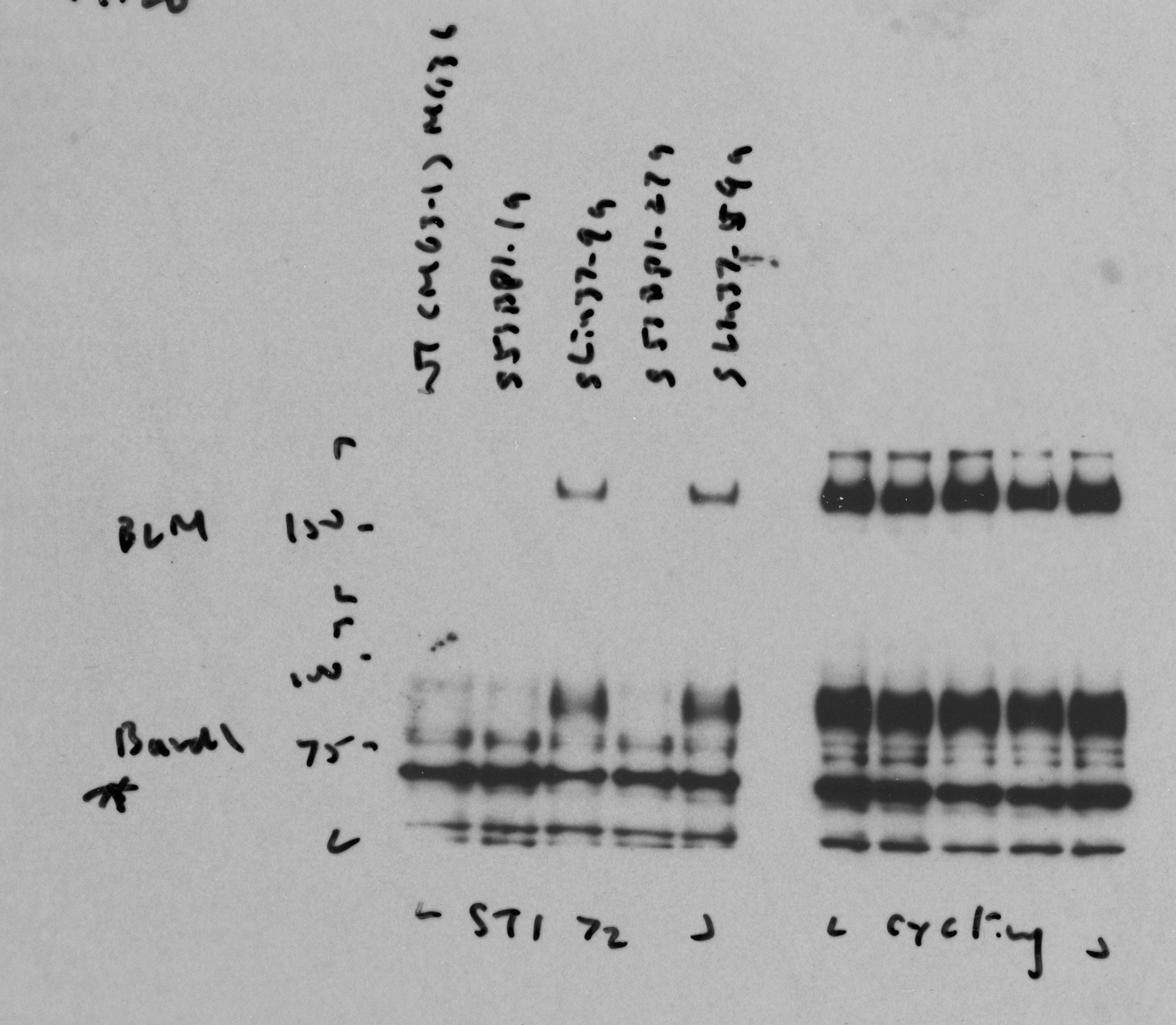

Supplement: Source data 5. [file elife-68466-data5.zip › Source data 5 - figure 5 part 2/Figure 5 S Source data/031920_BLM_Fig 5S1B0002.tif]

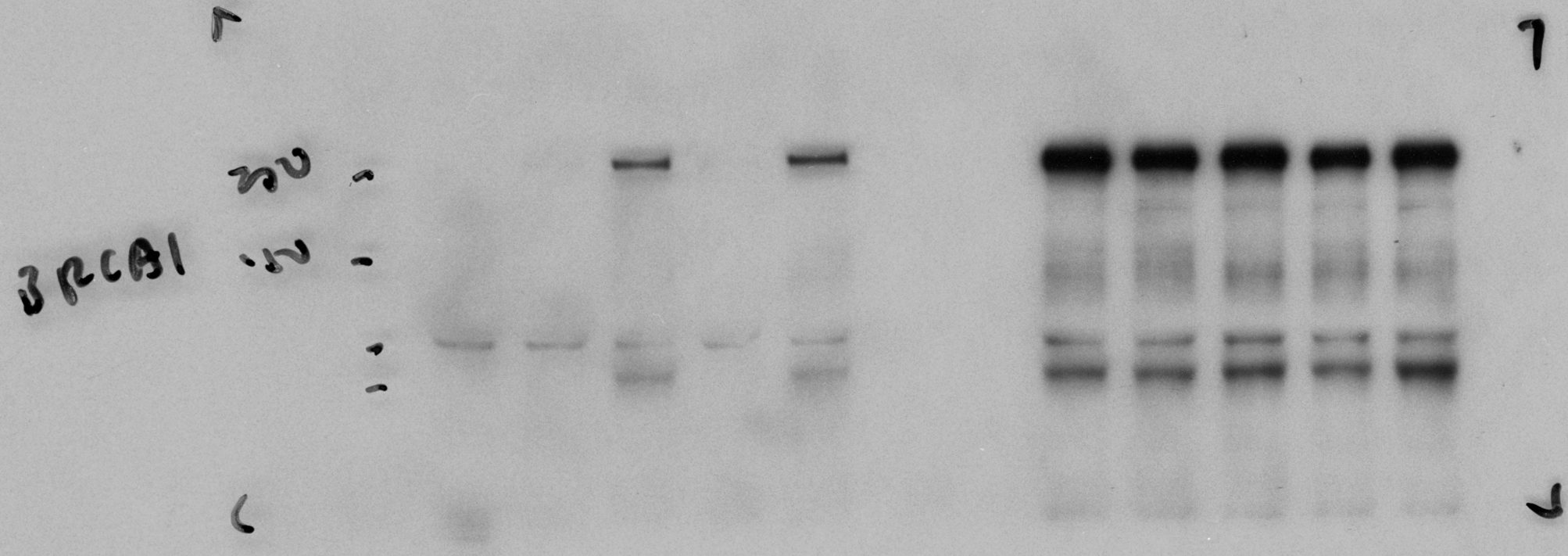

Supplement: Source data 5. [file elife-68466-data5.zip › Source data 5 - figure 5 part 2/Figure 5 S Source data/031220_6_BRCA1_Fig 5 S1B.tif]

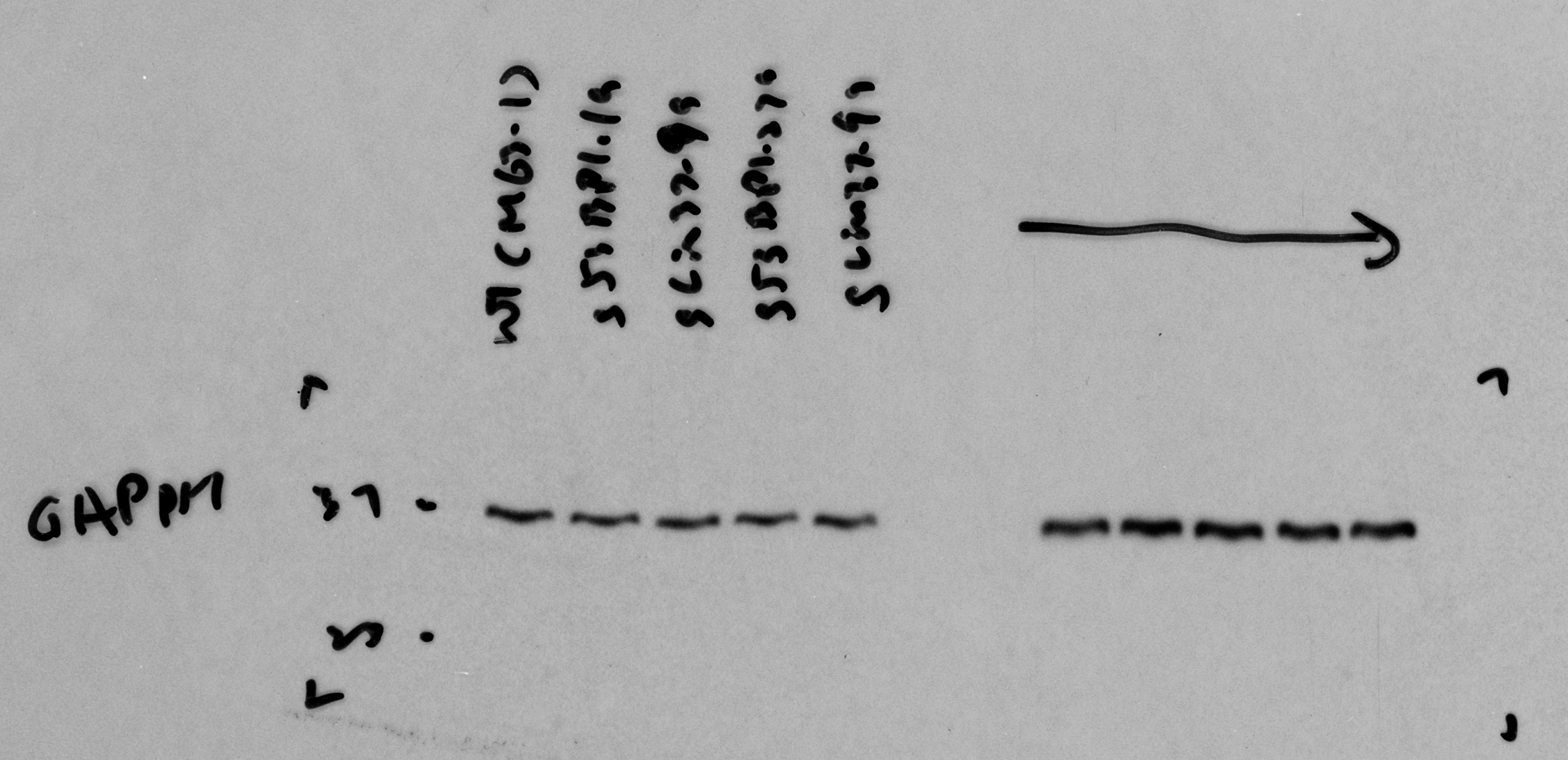

Supplement: Source data 5. [file elife-68466-data5.zip › Source data 5 - figure 5 part 2/Figure 5 S Source data/031220_1_GAPDH_Fig 5 S1B.tif]

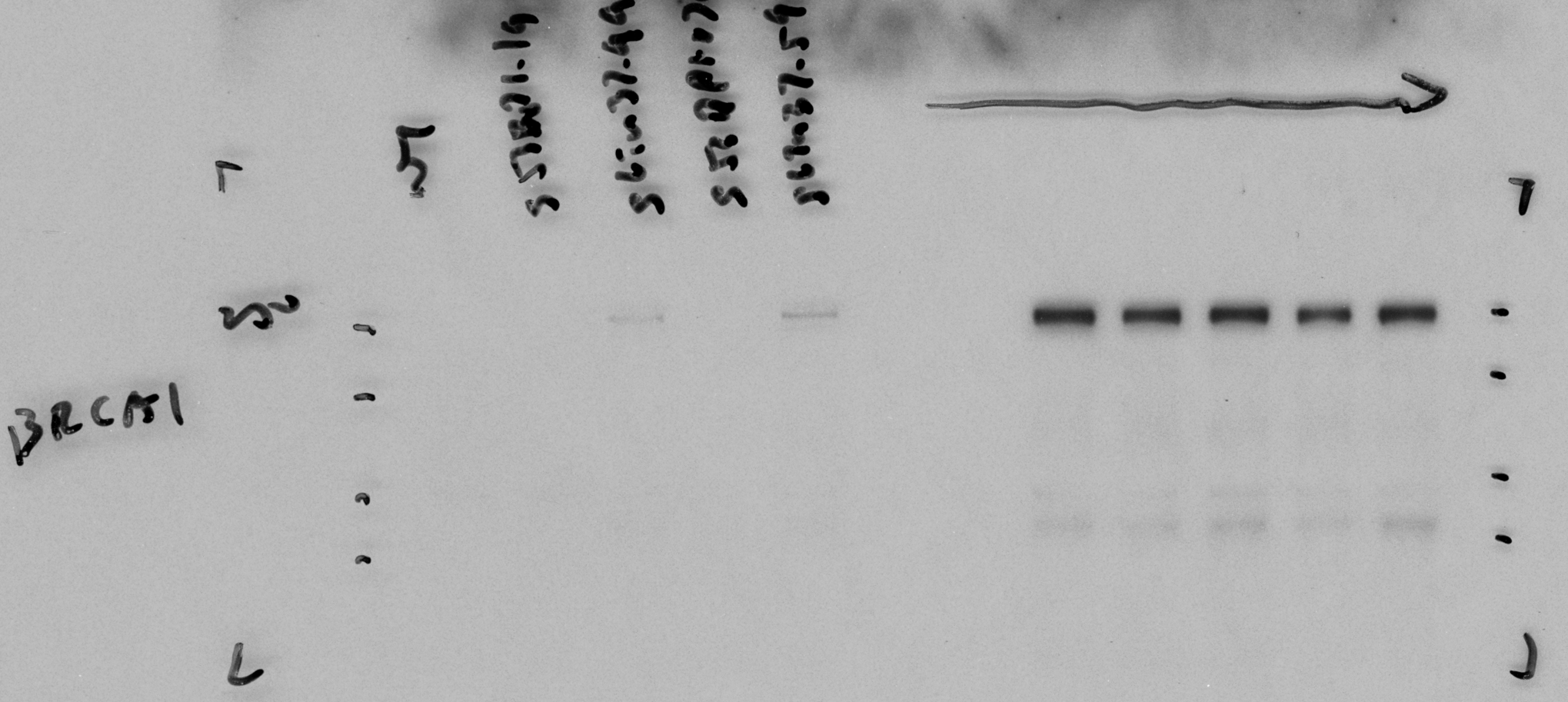

Supplement: Source data 5. [file elife-68466-data5.zip › Source data 5 - figure 5 part 2/Figure 5 S Source data/031220_7_BRCA1_Fig 5 S1B.tif]

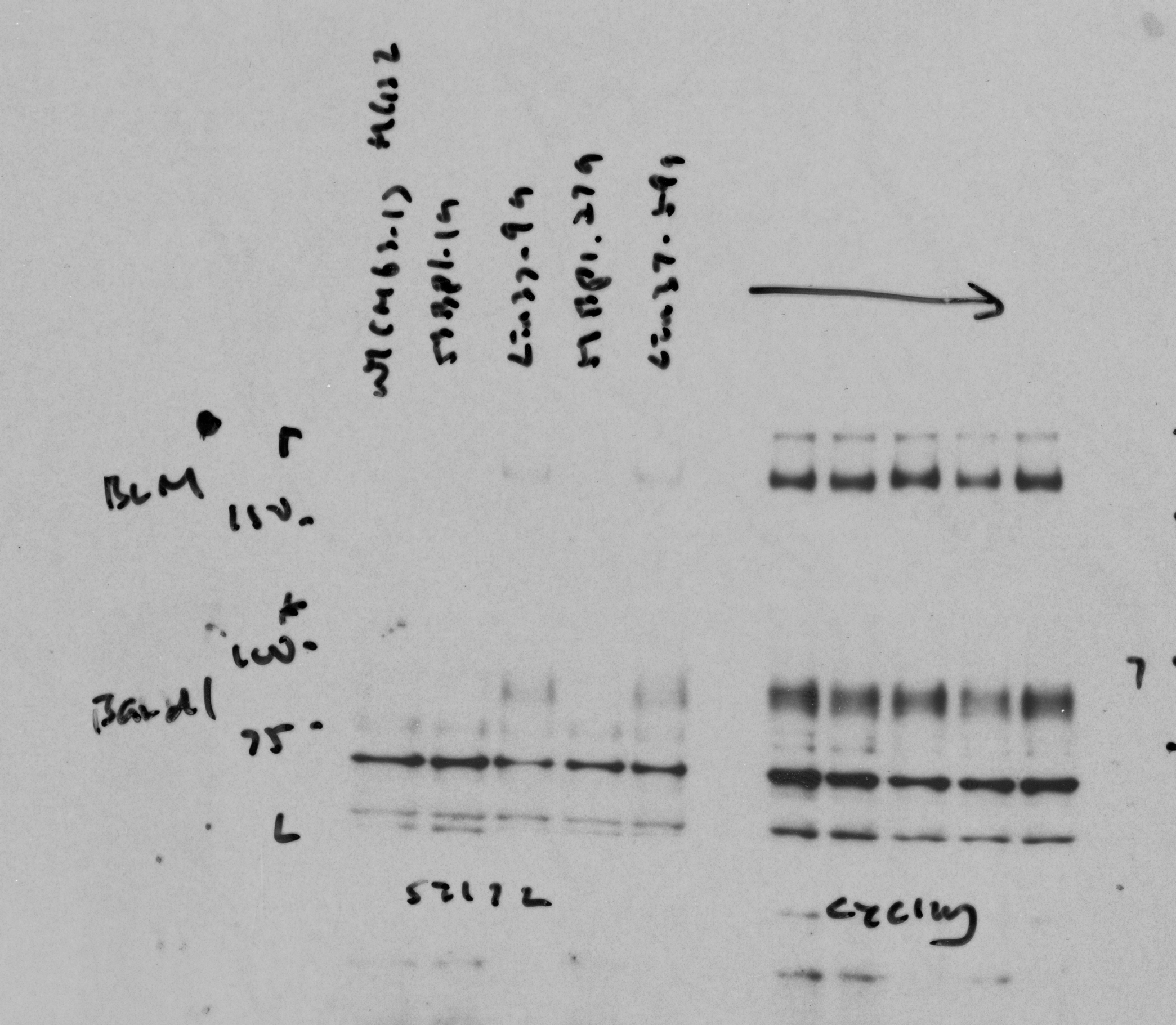

Supplement: Source data 5. [file elife-68466-data5.zip › Source data 5 - figure 5 part 2/Figure 5 S Source data/031920_BLM_Fig 5S1B0001.tif]

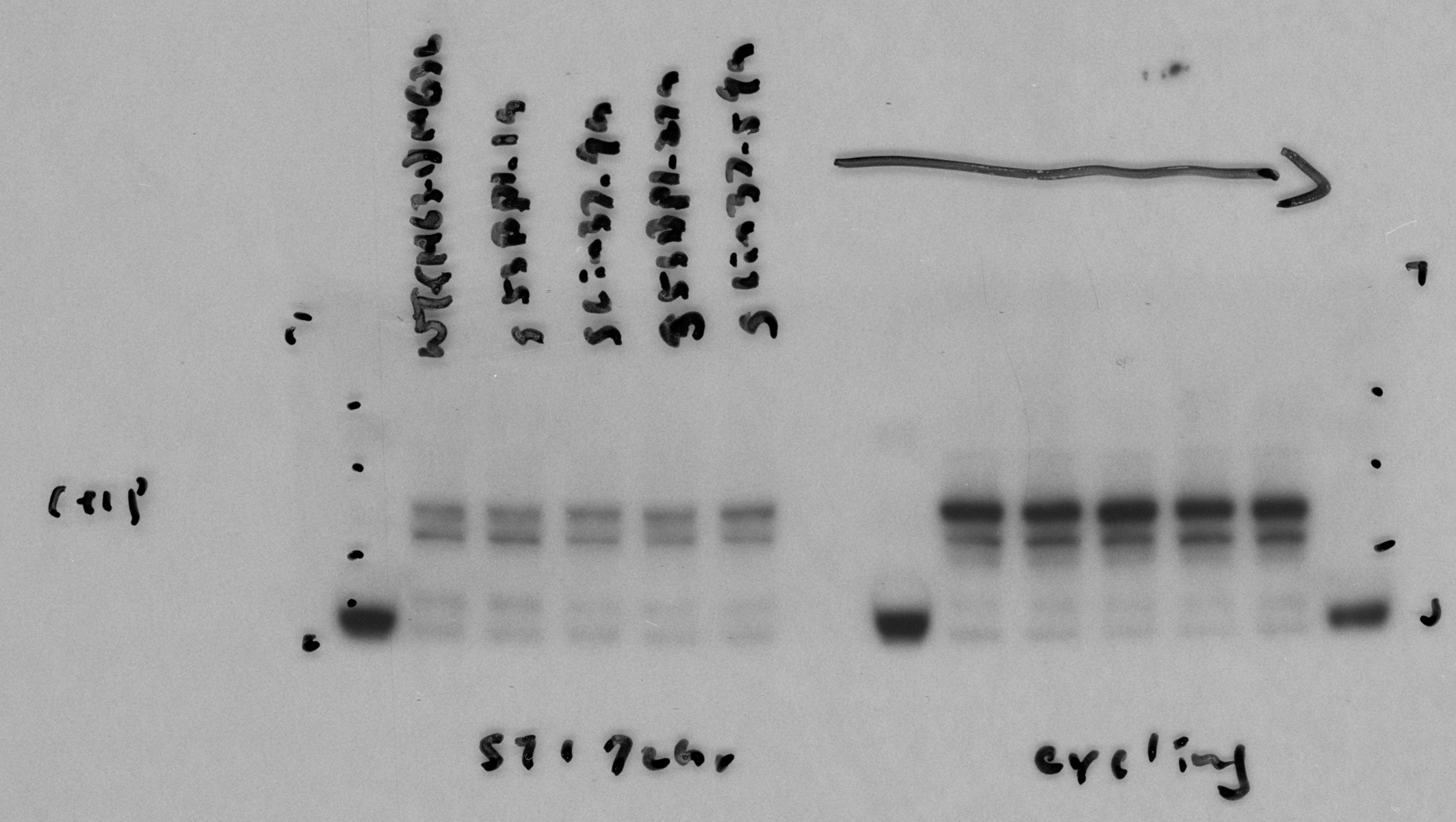

Supplement: Source data 5. [file elife-68466-data5.zip › Source data 5 - figure 5 part 2/Figure 5 S Source data/031320_1_CtIP_5 S1B.tif]

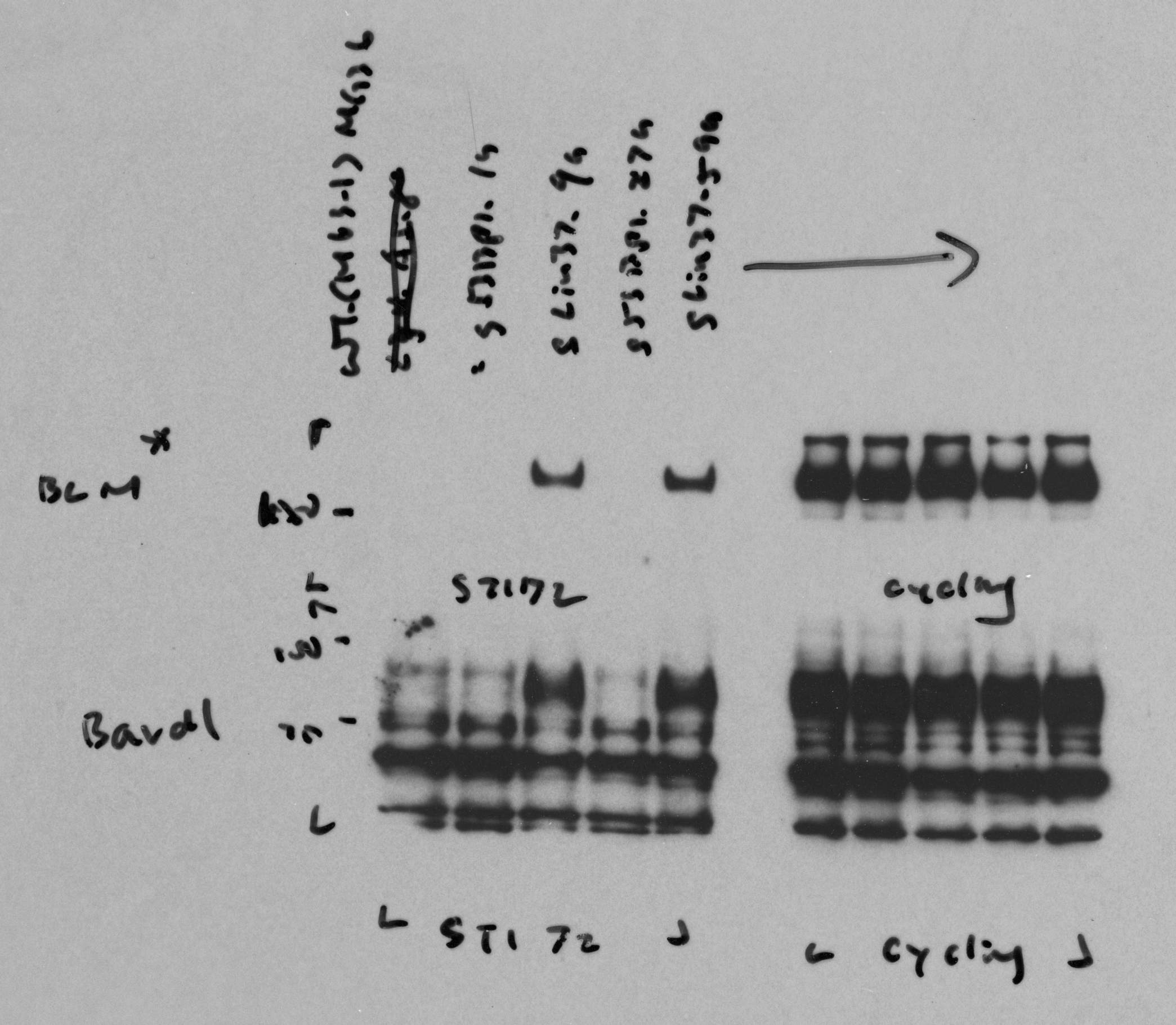

Supplement: Source data 5. [file elife-68466-data5.zip › Source data 5 - figure 5 part 2/Figure 5 S Source data/031920_BLM_Fig 5S1B.tif]

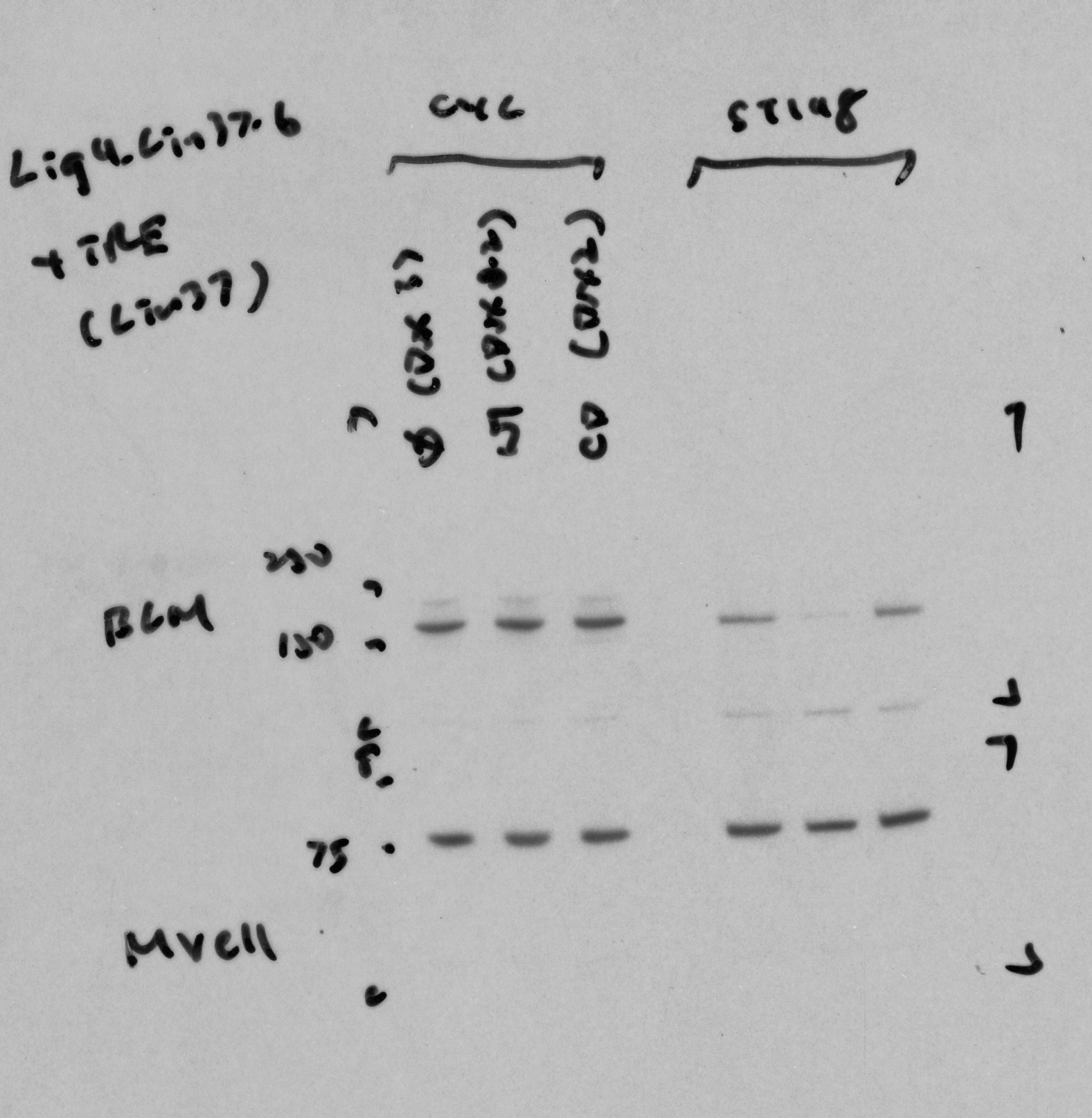

Supplement: Source data 5. [file elife-68466-data5.zip › Source data 5 - figure 5 part 2/Figure 5 S Source data/051120_Mre11_Fig 5E.tif]

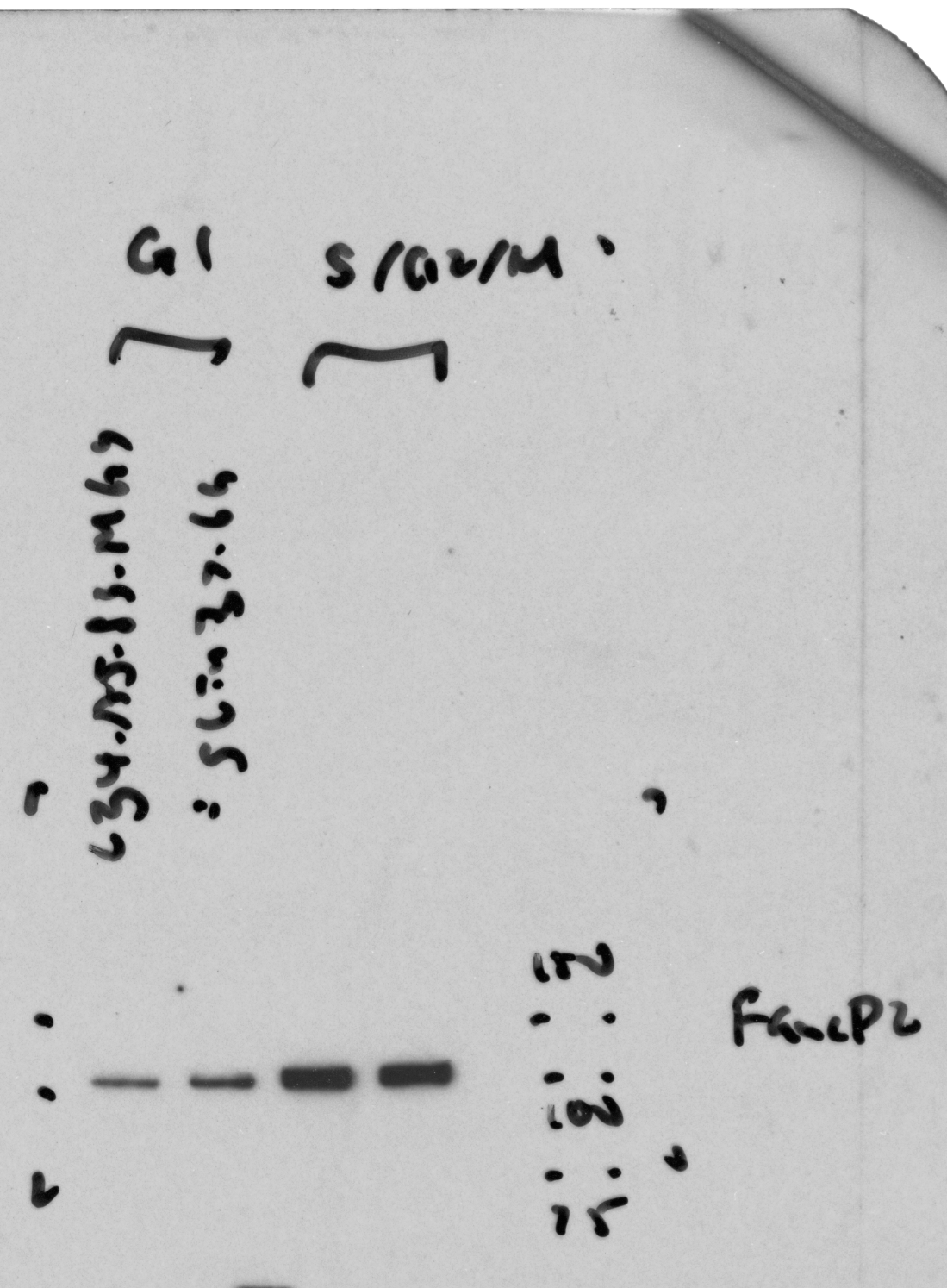

Supplement: Source data 6. [file elife-68466-data6.zip › Source data 6 - figure 6 and 7/Figure 7/100220_FANCD2_Fig 7C.tif]

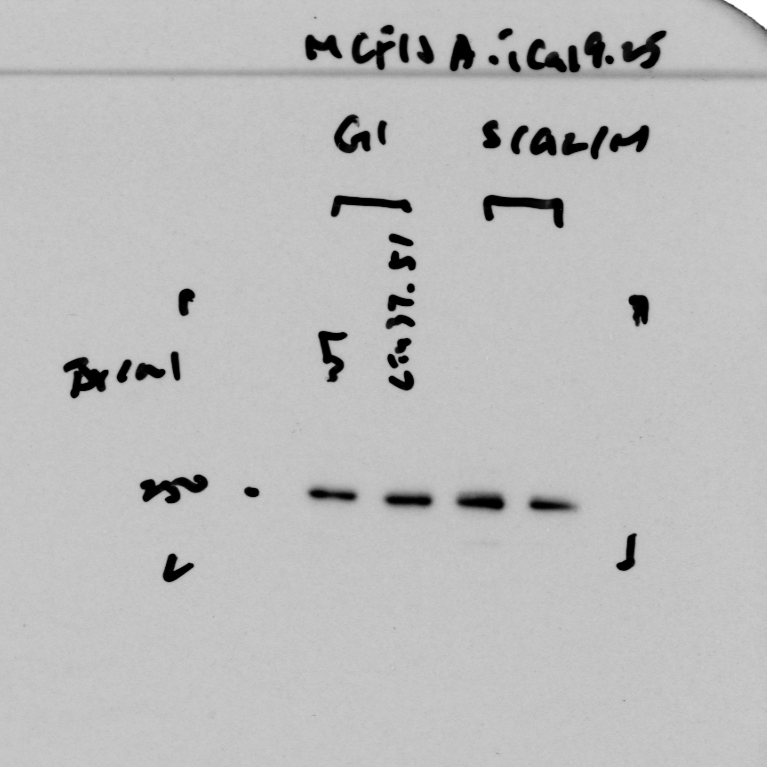

Supplement: Source data 6. [file elife-68466-data6.zip › Source data 6 - figure 6 and 7/Figure 7/1014200002_BRCA1_Fig 7D.tif]

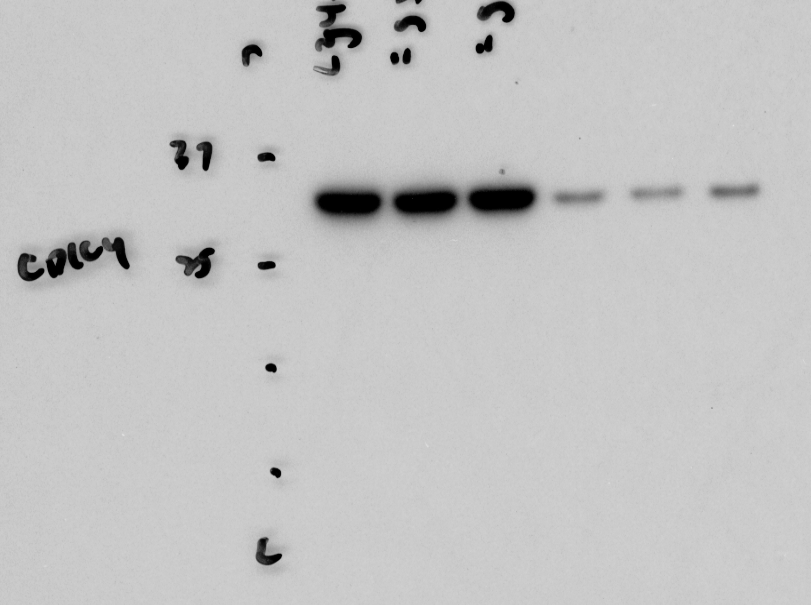

Supplement: Source data 6. [file elife-68466-data6.zip › Source data 6 - figure 6 and 7/Figure 7/0926200001_CDK4_Fig 7A.tif]

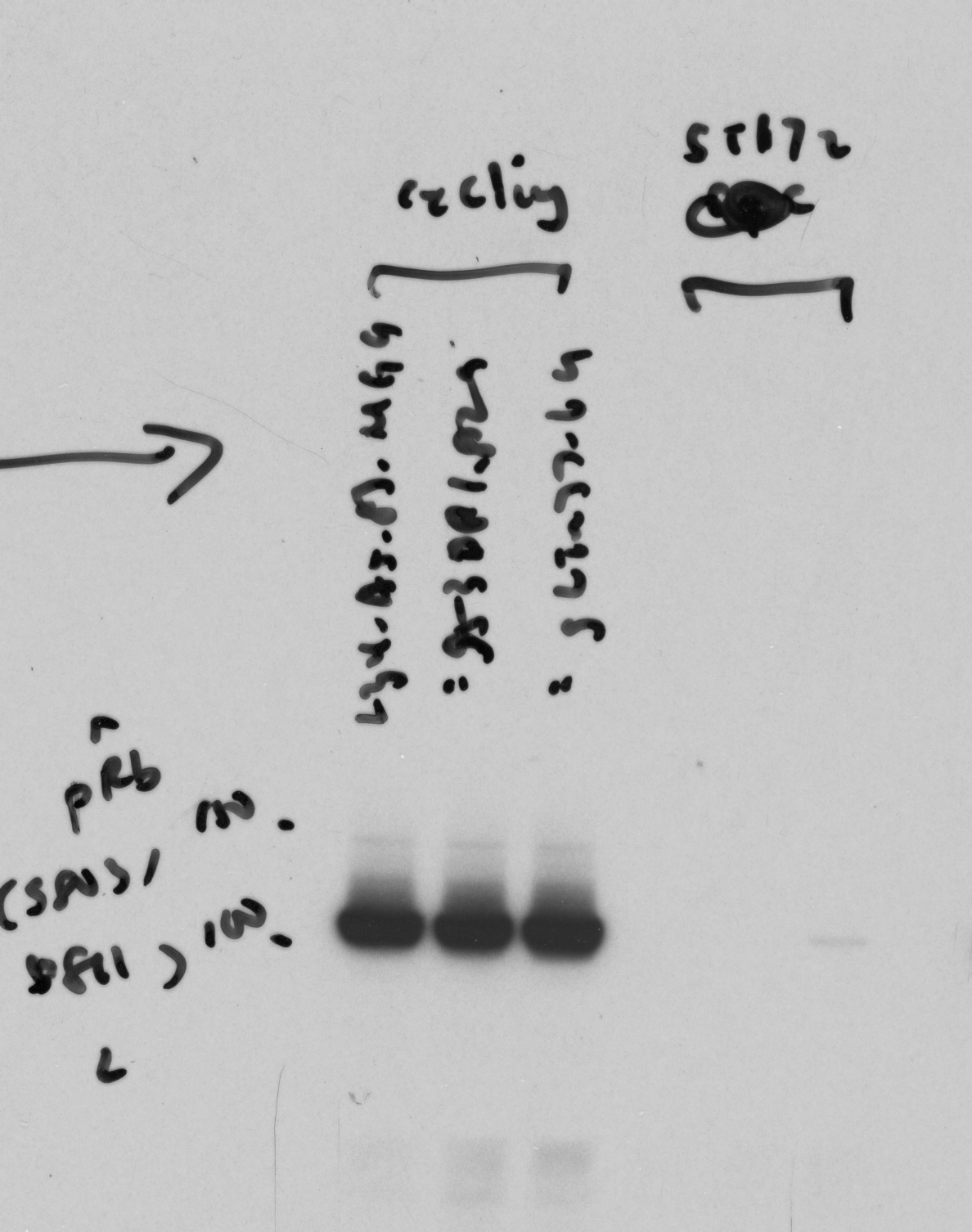

Supplement: Source data 6. [file elife-68466-data6.zip › Source data 6 - figure 6 and 7/Figure 7/091020_pRB S803_Fig 7A.tif]

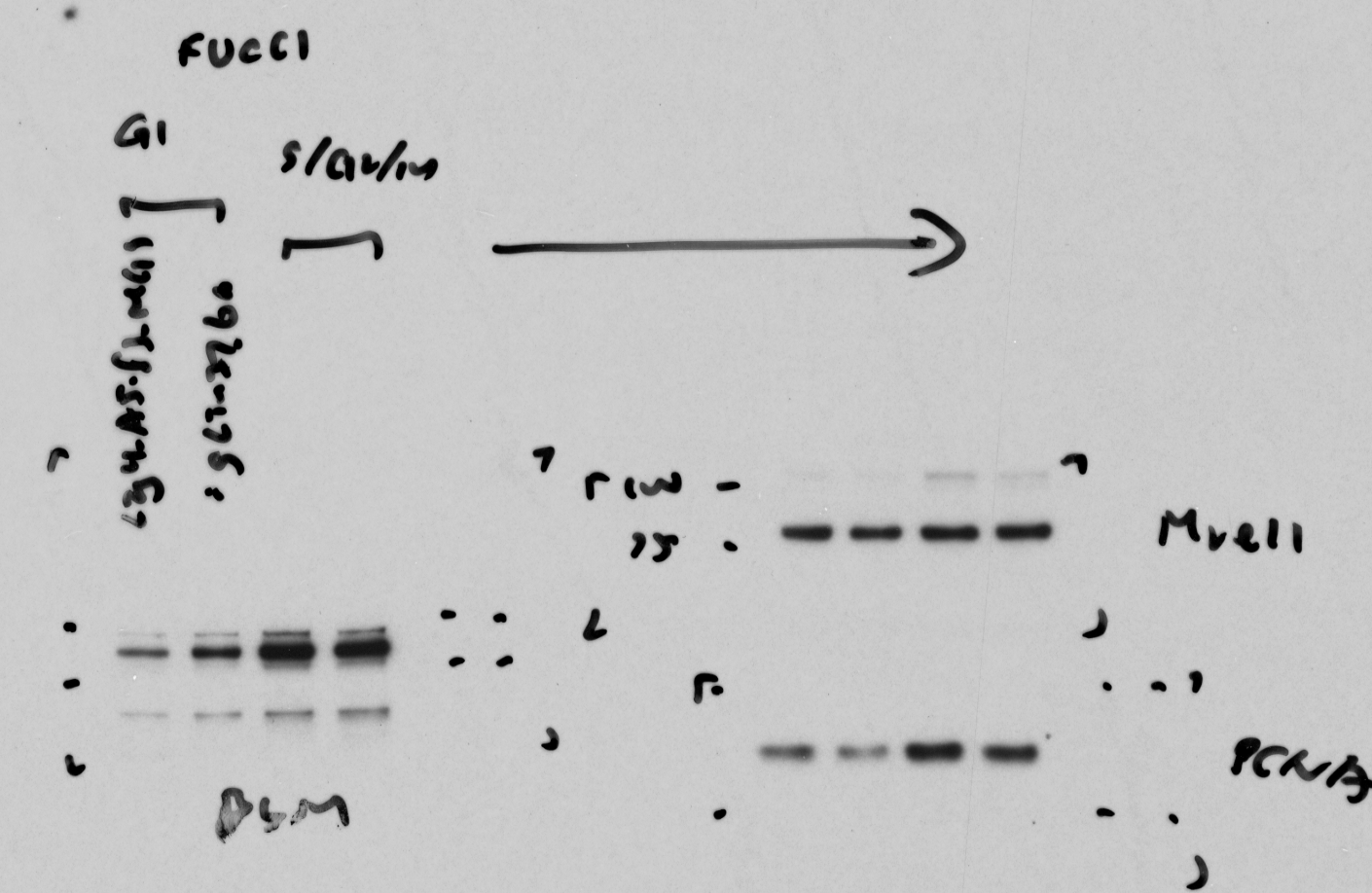

Supplement: Source data 6. [file elife-68466-data6.zip › Source data 6 - figure 6 and 7/Figure 7/1006200005_BLM, MRE11_Fig 7C.tif]

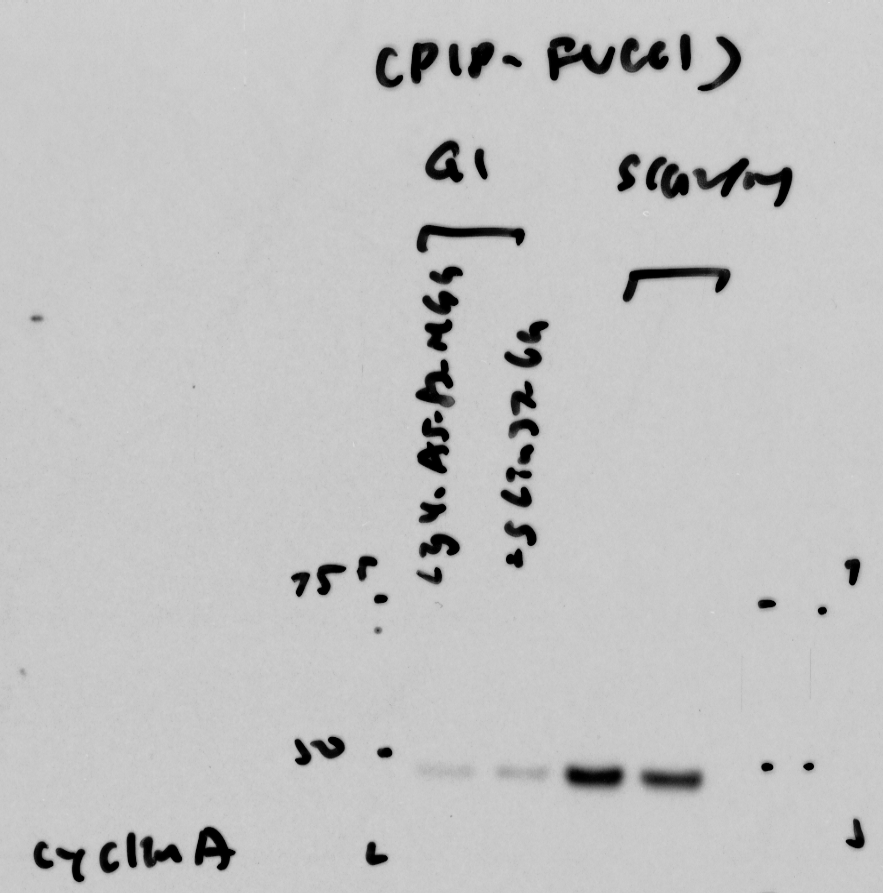

Supplement: Source data 6. [file elife-68466-data6.zip › Source data 6 - figure 6 and 7/Figure 7/1008200002_cyclin A_Fig 7C.tif]

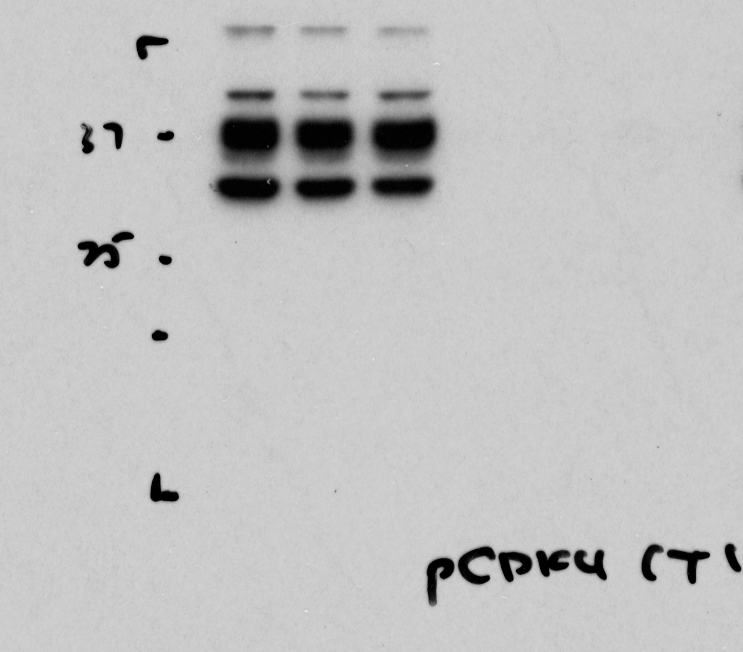

Supplement: Source data 6. [file elife-68466-data6.zip › Source data 6 - figure 6 and 7/Figure 7/0925200002_pCDK4_Fig 7A.tif]

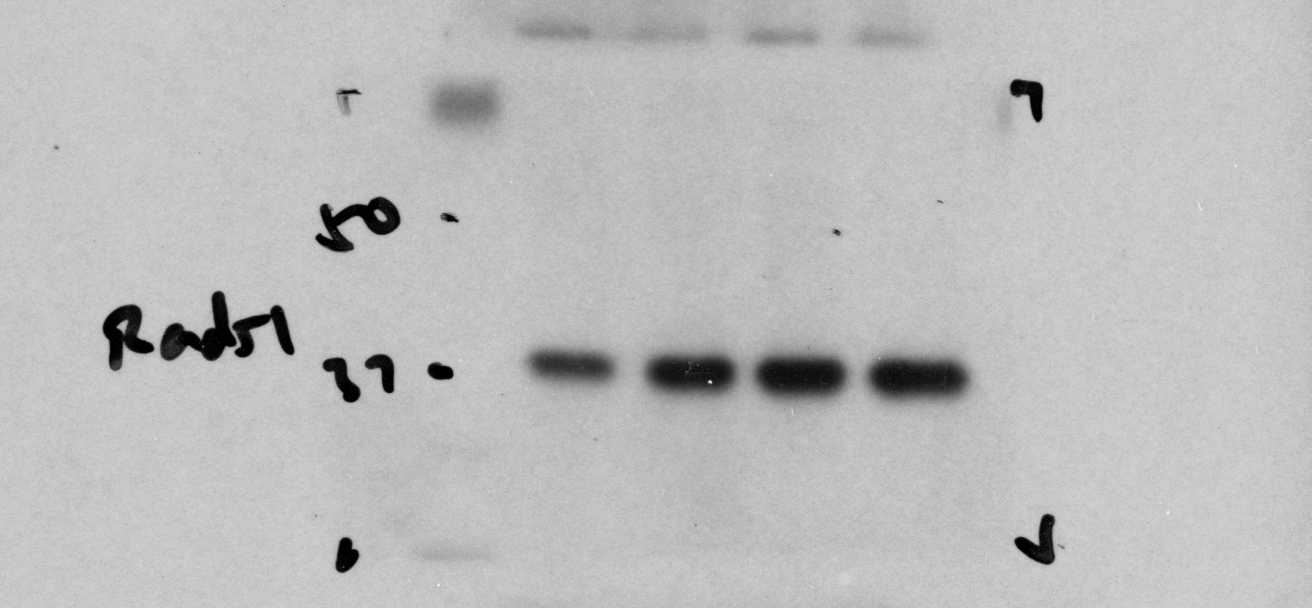

Supplement: Source data 6. [file elife-68466-data6.zip › Source data 6 - figure 6 and 7/Figure 7/101420_RAD51_Fig 7D.tif]

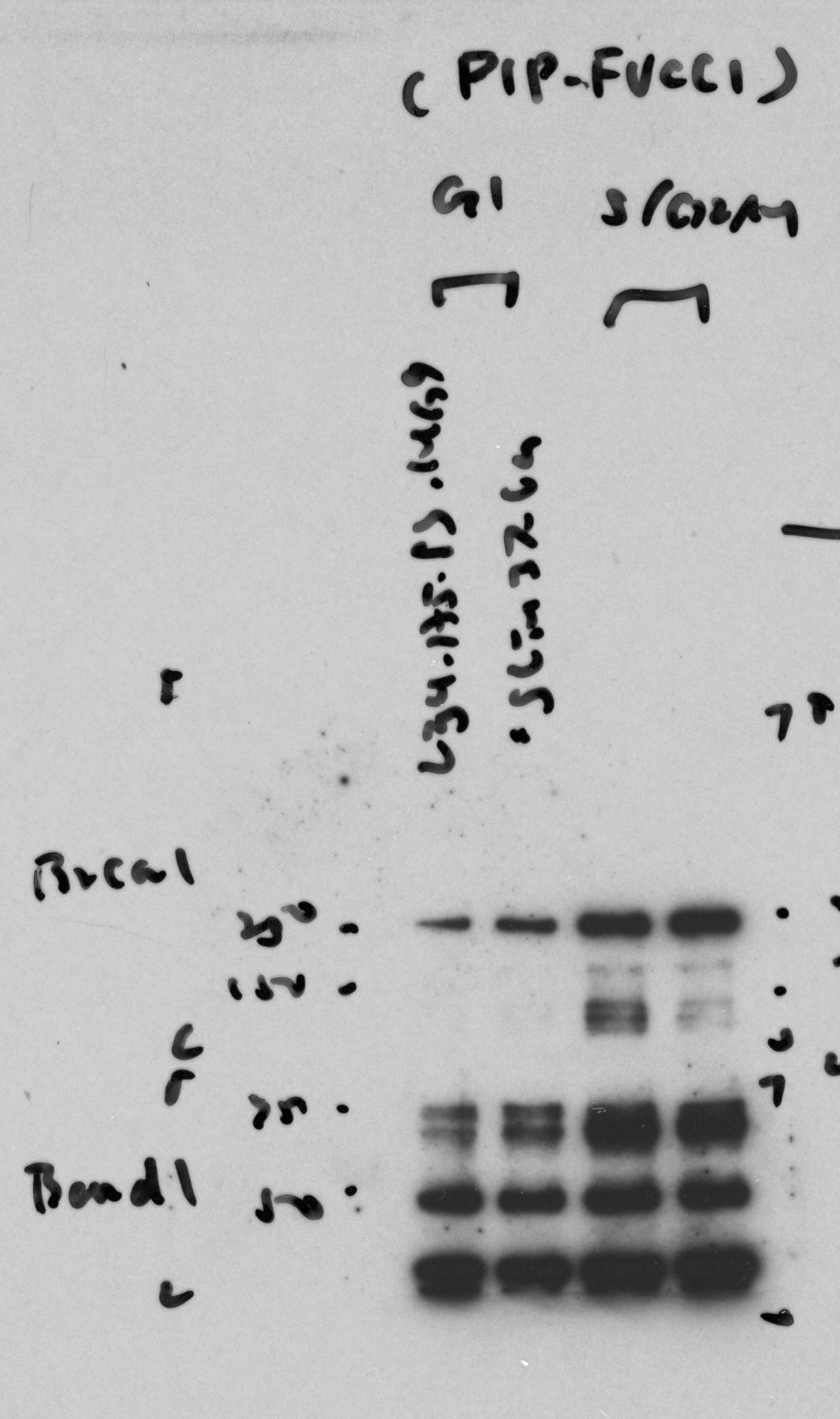

Supplement: Source data 6. [file elife-68466-data6.zip › Source data 6 - figure 6 and 7/Figure 7/100220_BRCA1-BARD1_Fig 7C.tif]

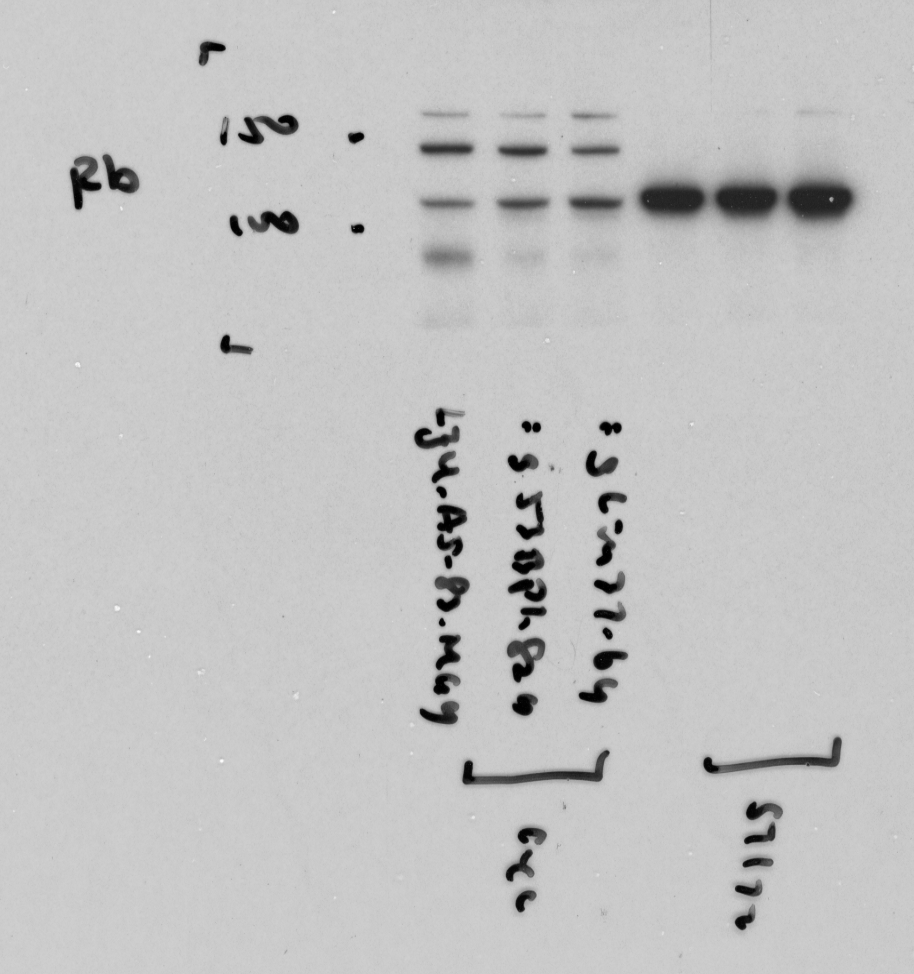

Supplement: Source data 6. [file elife-68466-data6.zip › Source data 6 - figure 6 and 7/Figure 7/0911200002_RB_Fig 7A.tif]

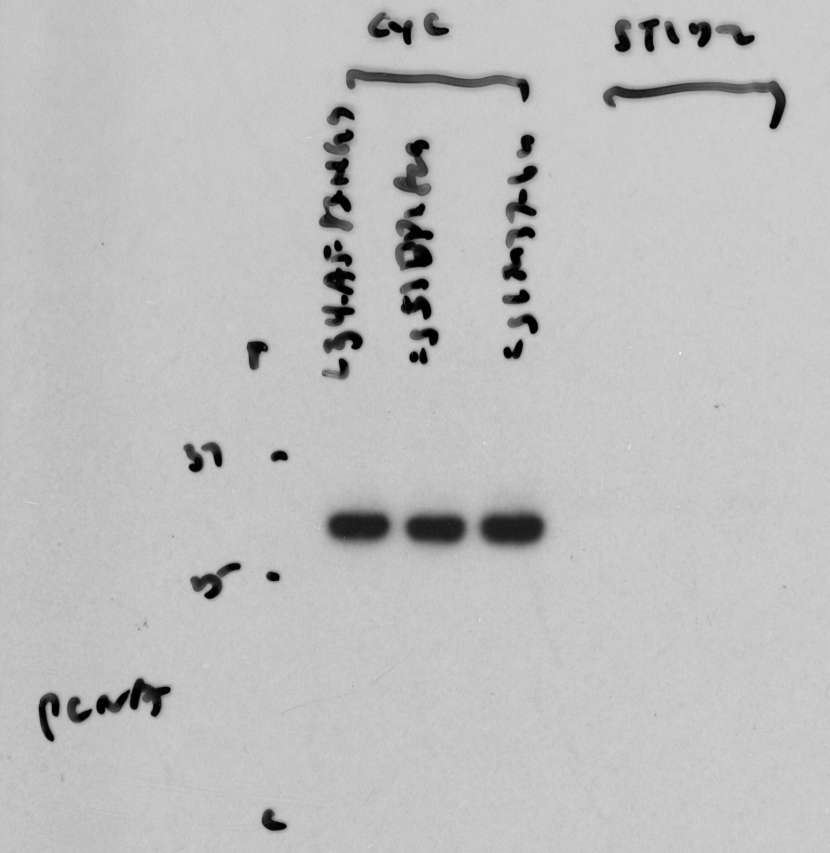

Supplement: Source data 6. [file elife-68466-data6.zip › Source data 6 - figure 6 and 7/Figure 7/1006200004_PCNA_Fig7A.tif]

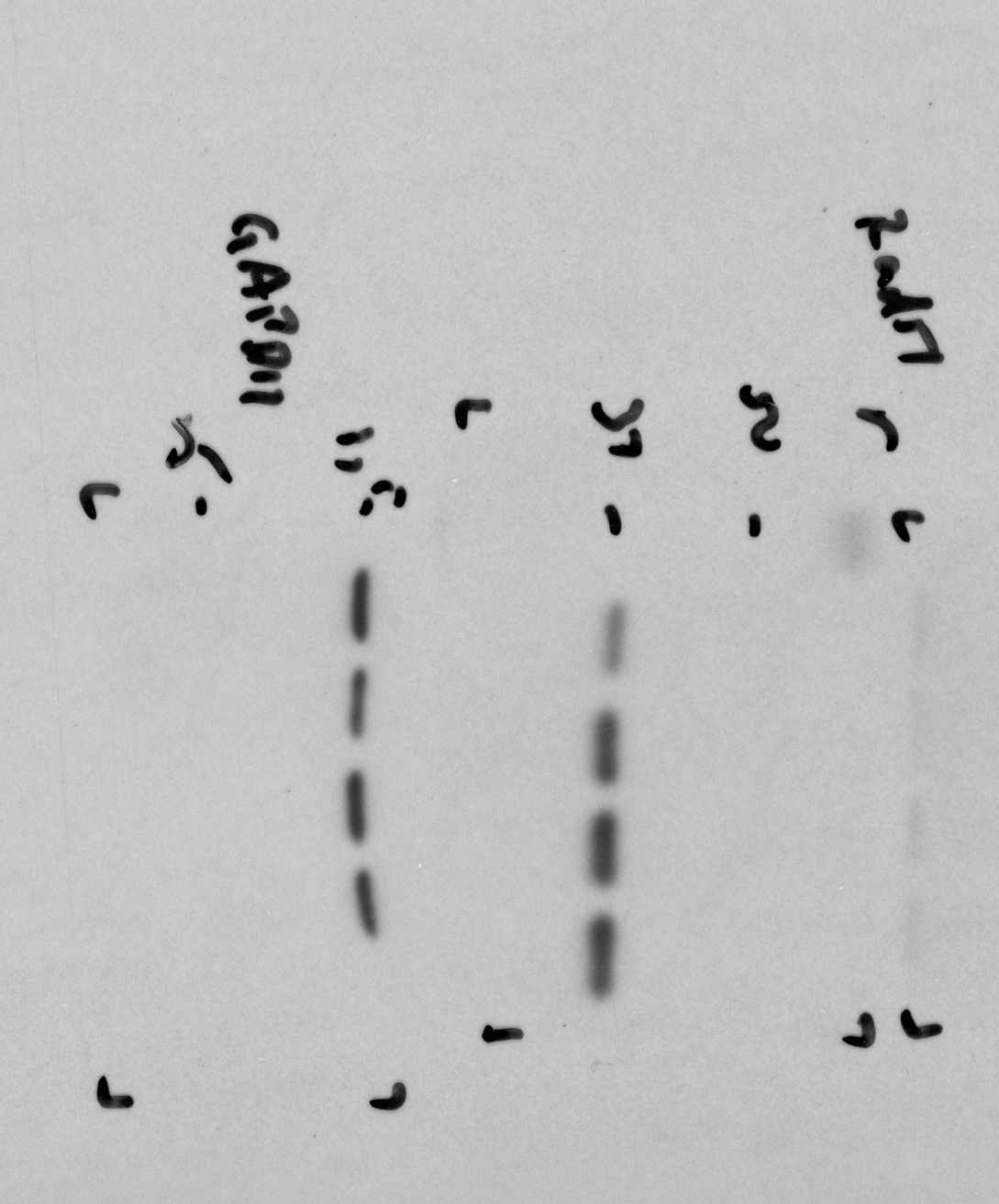

Supplement: Source data 6. [file elife-68466-data6.zip › Source data 6 - figure 6 and 7/Figure 7/101420_GAPDH_Fig 7D0001.tif]

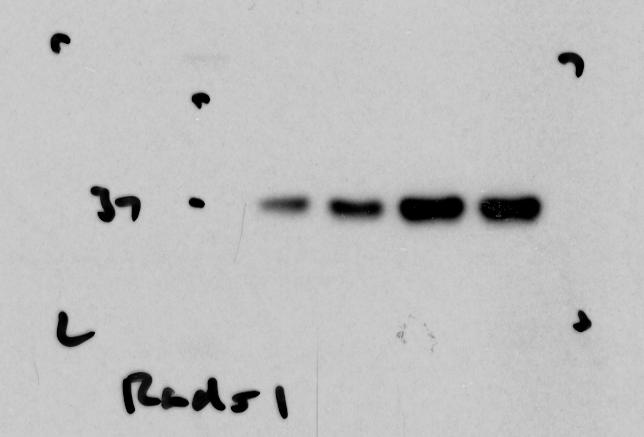

Supplement: Source data 6. [file elife-68466-data6.zip › Source data 6 - figure 6 and 7/Figure 7/1002200001_RAD51_Fig 7C.tif]

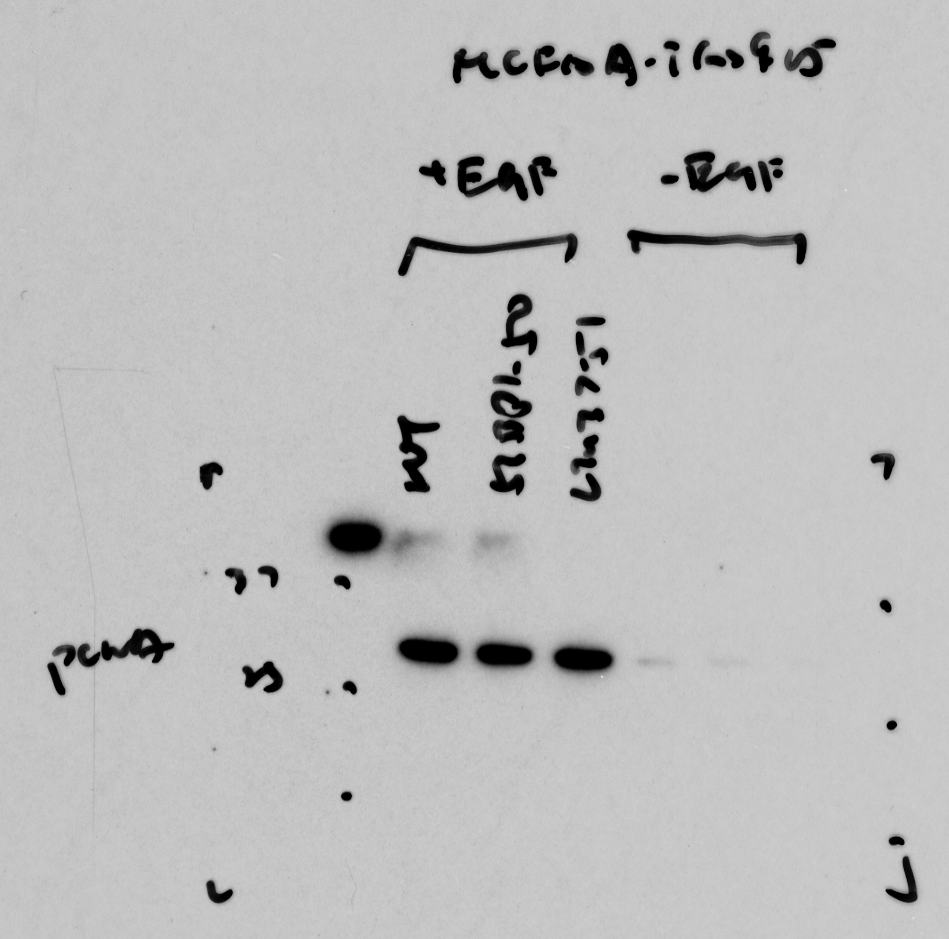

Supplement: Source data 6. [file elife-68466-data6.zip › Source data 6 - figure 6 and 7/Figure 7/100720_PCNA_Fig 7B.tif]

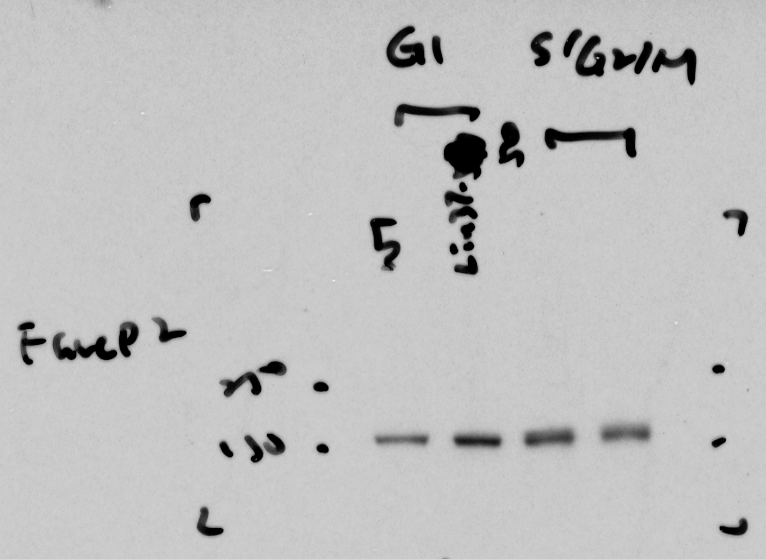

Supplement: Source data 6. [file elife-68466-data6.zip › Source data 6 - figure 6 and 7/Figure 7/102320_FANCD2_Fig 7D.tif]

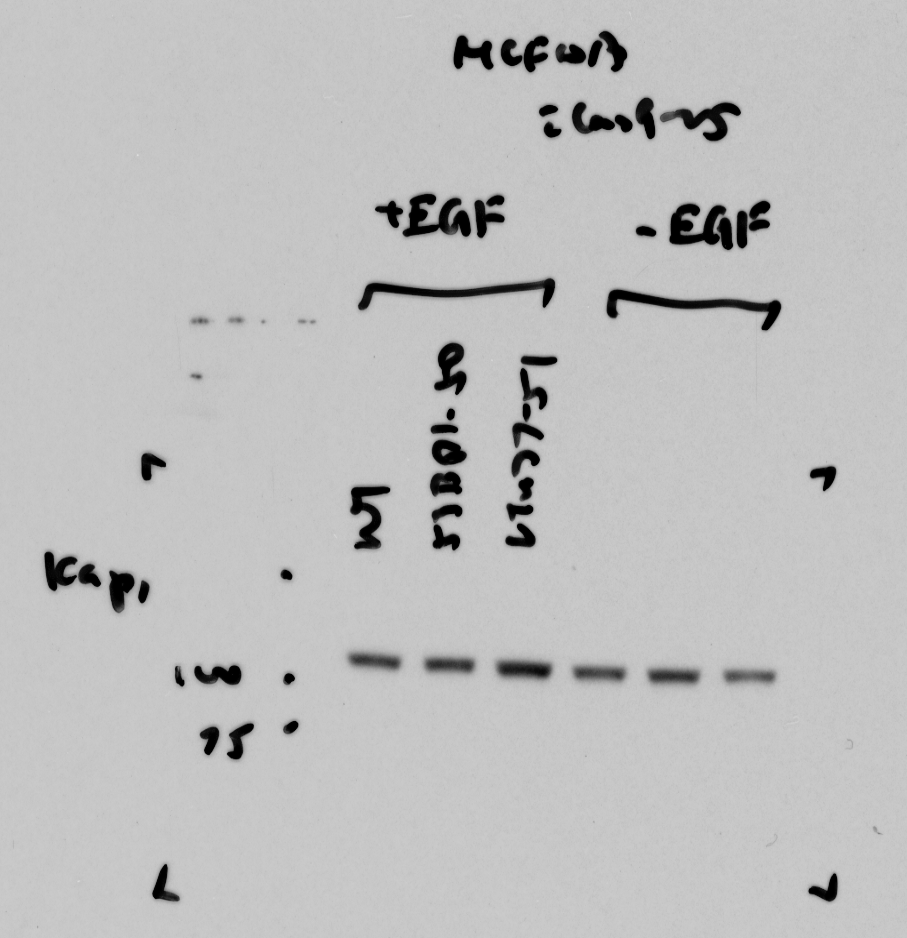

Supplement: Source data 6. [file elife-68466-data6.zip › Source data 6 - figure 6 and 7/Figure 7/1007200001_KAP1_Fig 7B.tif]
