# Supplementary figures and images for: LIN37-DREAM prevents DNA end resection and homologous recombination at DNA double-strand breaks in quiescent cells (part 2 of 2)
Source: eLife. 2021 Sep 3;10:e68466. doi: 10.7554/eLife.68466 (PMC8416021; doi:10.7554/eLife.68466)

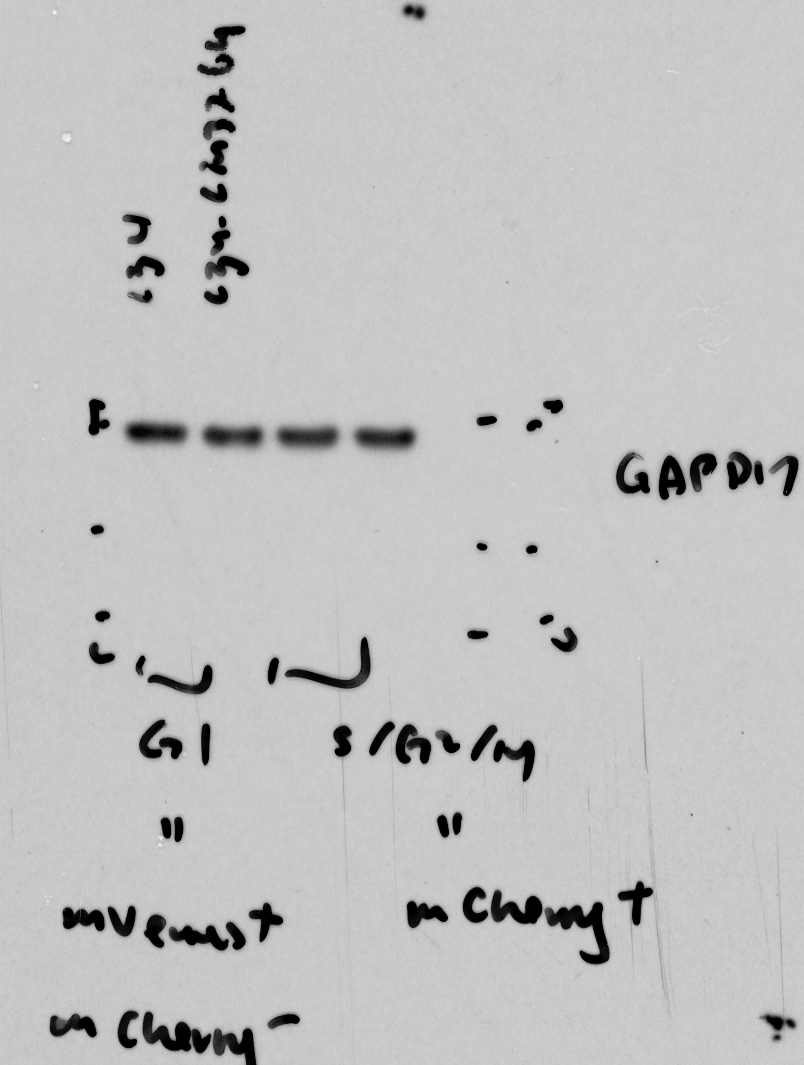

Supplement: Source data 6. [file elife-68466-data6.zip › Source data 6 - figure 6 and 7/Figure 7/1002200005_GAPDH_Fig 7C.tif]

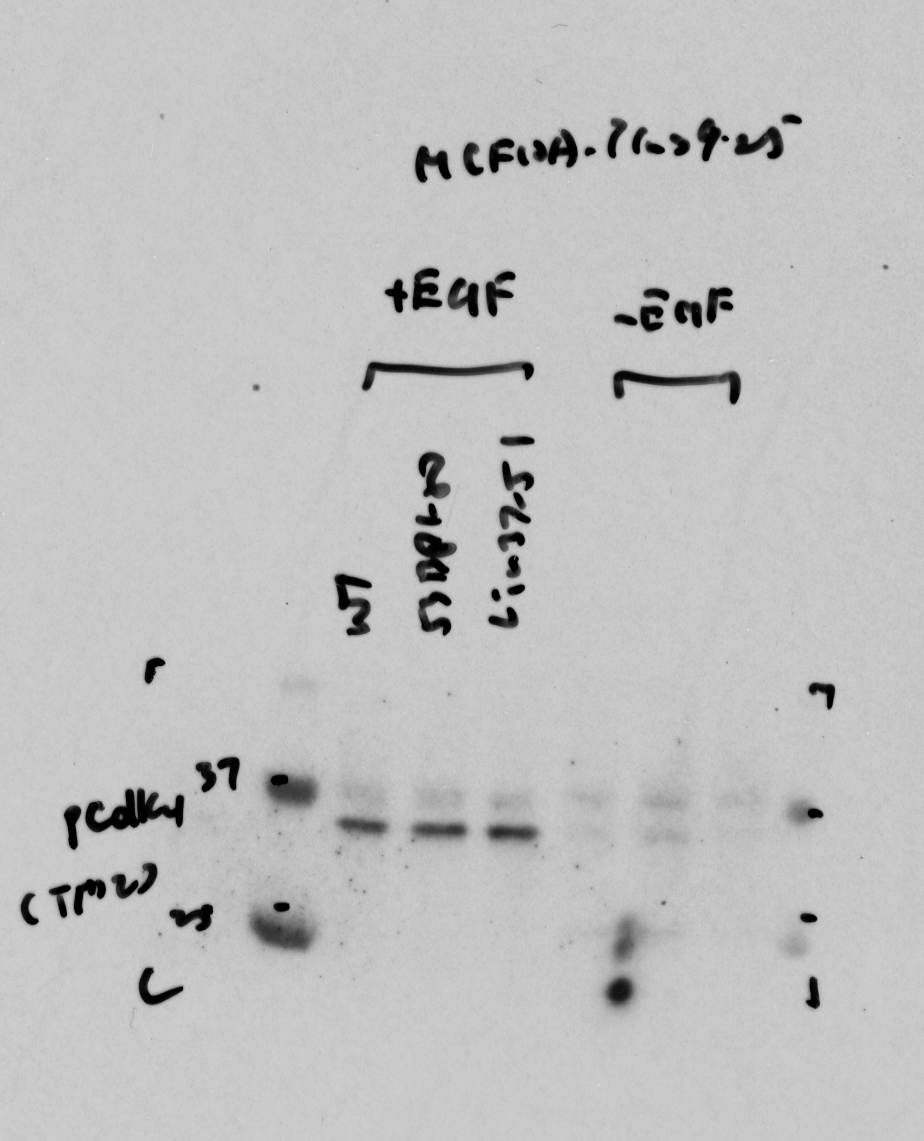

Supplement: Source data 6. [file elife-68466-data6.zip › Source data 6 - figure 6 and 7/Figure 7/1008200001_pCDK4_Fig 7B.tif]

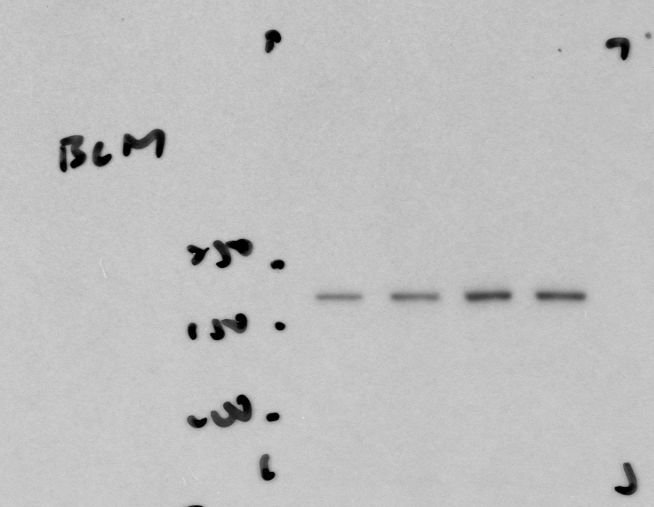

Supplement: Source data 6. [file elife-68466-data6.zip › Source data 6 - figure 6 and 7/Figure 7/1014200004_BLM_Fig 7D.tif]

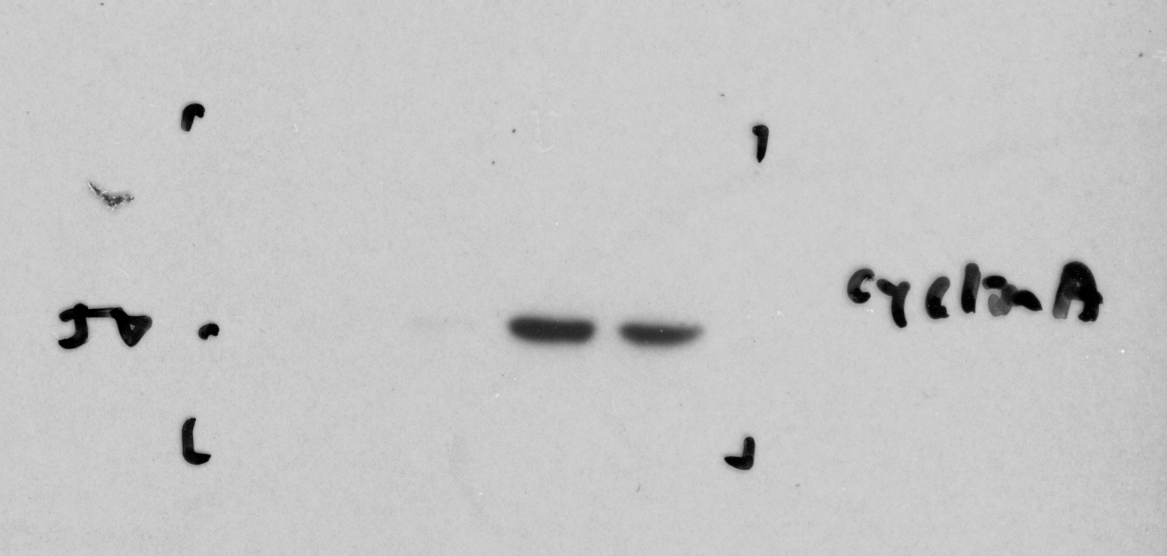

Supplement: Source data 6. [file elife-68466-data6.zip › Source data 6 - figure 6 and 7/Figure 7/101420_Gcyclin A_Fig 7D.tif]

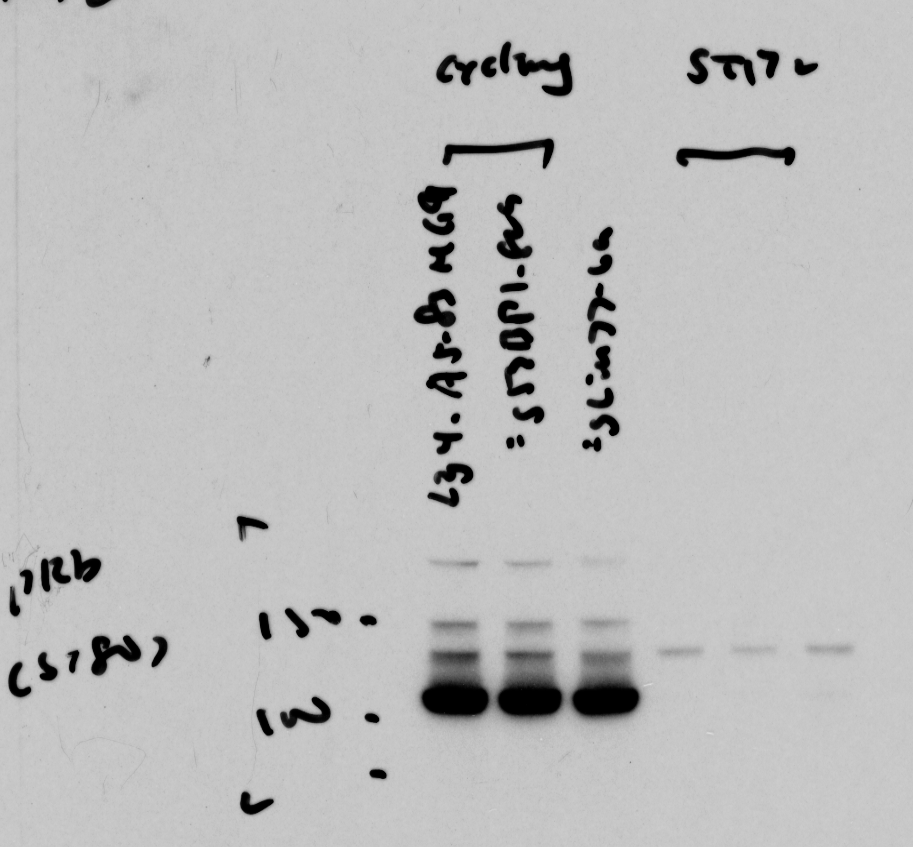

Supplement: Source data 6. [file elife-68466-data6.zip › Source data 6 - figure 6 and 7/Figure 7/0910200001_pRB_Fig 7A.tif]

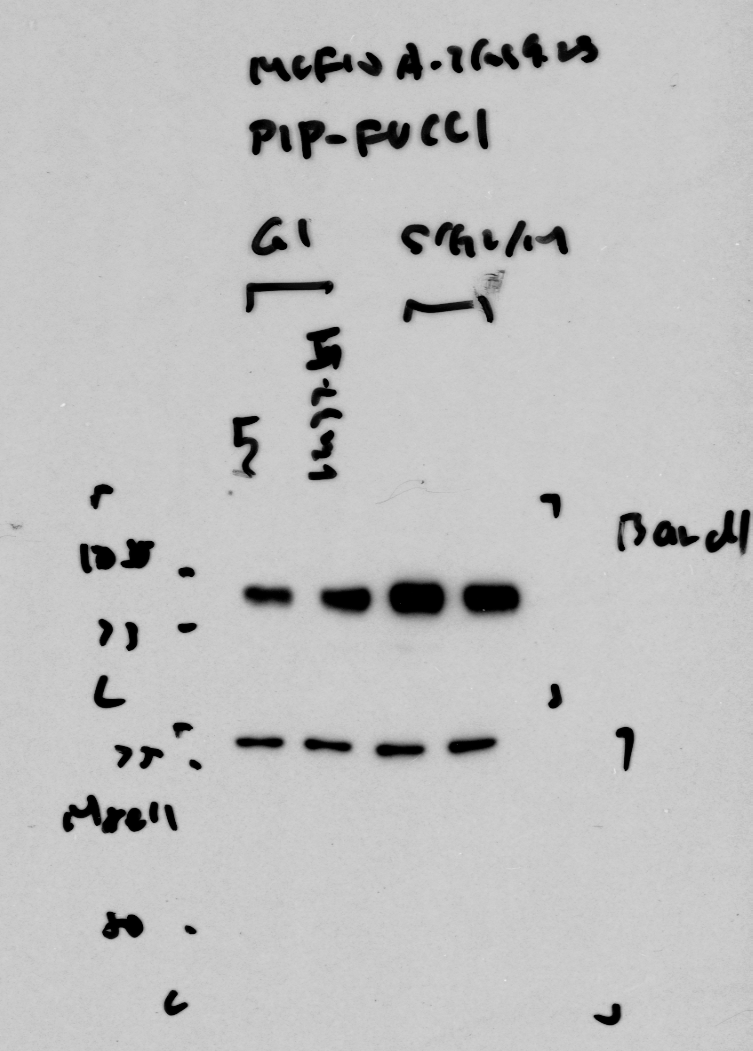

Supplement: Source data 6. [file elife-68466-data6.zip › Source data 6 - figure 6 and 7/Figure 7/101420._Bar1, MRE11_Fig 7Dtif.tif]

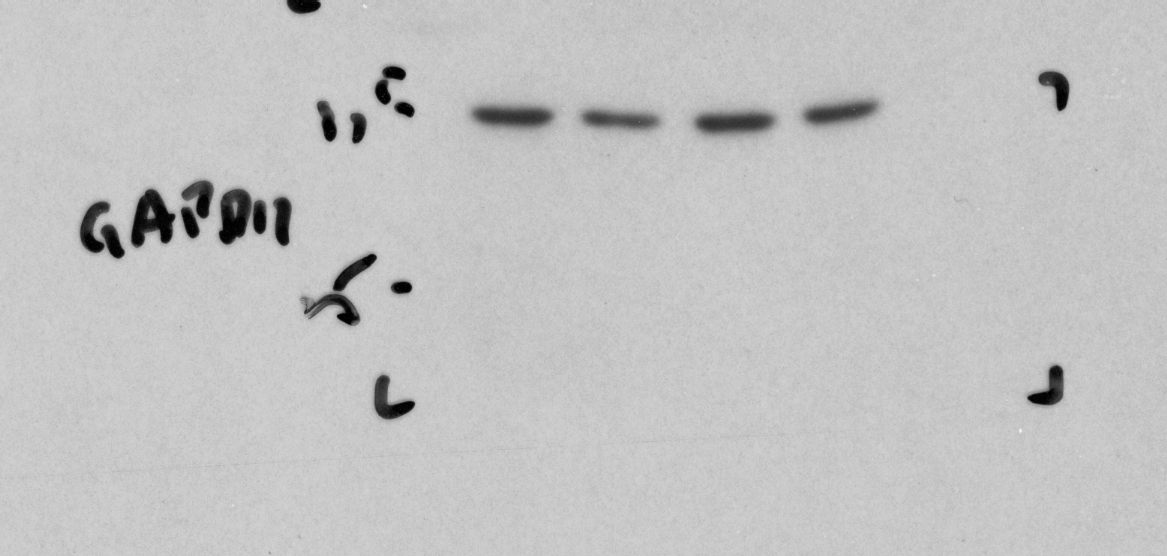

Supplement: Source data 6. [file elife-68466-data6.zip › Source data 6 - figure 6 and 7/Figure 7/101420_GAPDH_Fig 7D.tif]

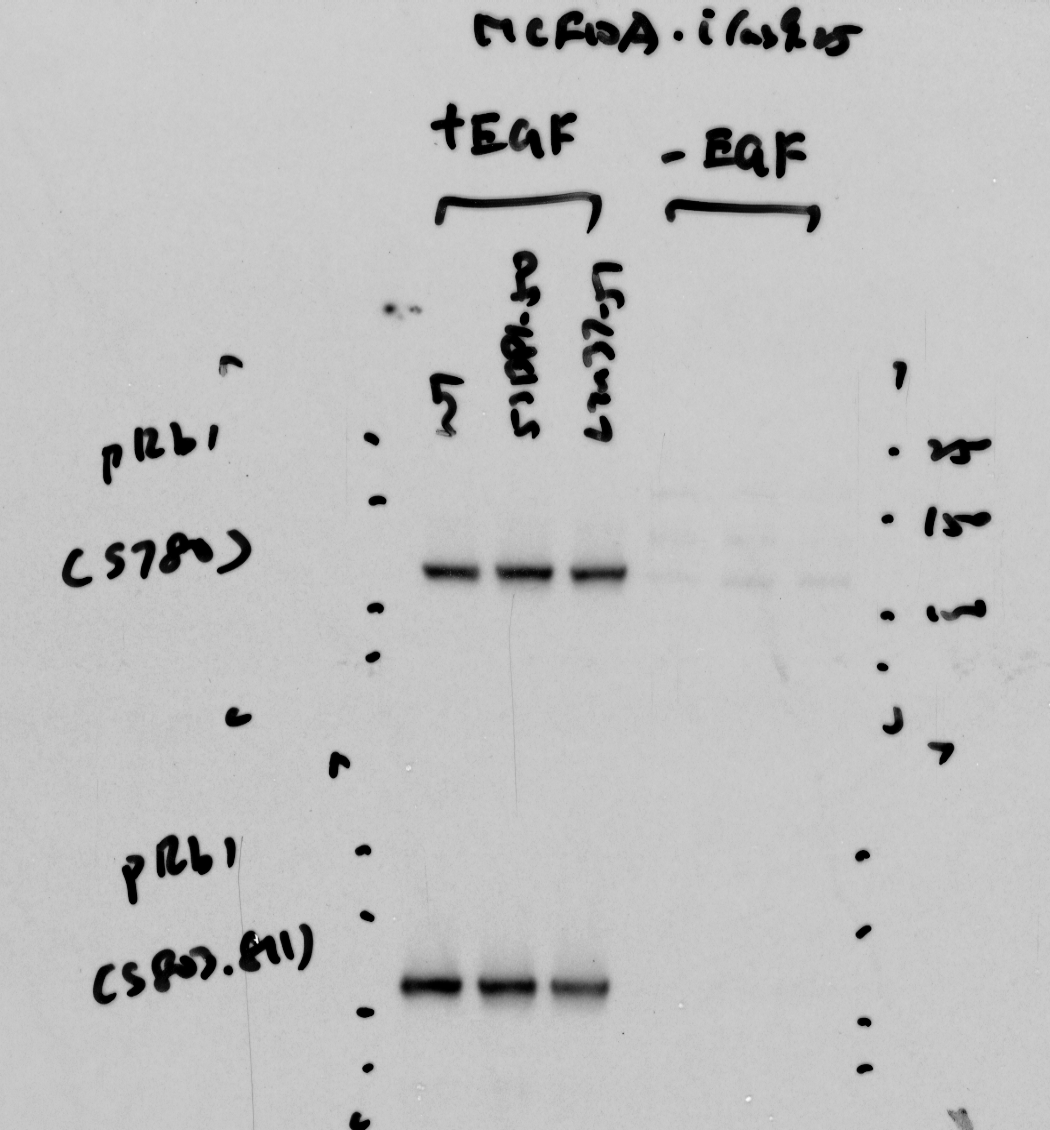

Supplement: Source data 6. [file elife-68466-data6.zip › Source data 6 - figure 6 and 7/Figure 7/100620_pRB_Fig 7B.tif]

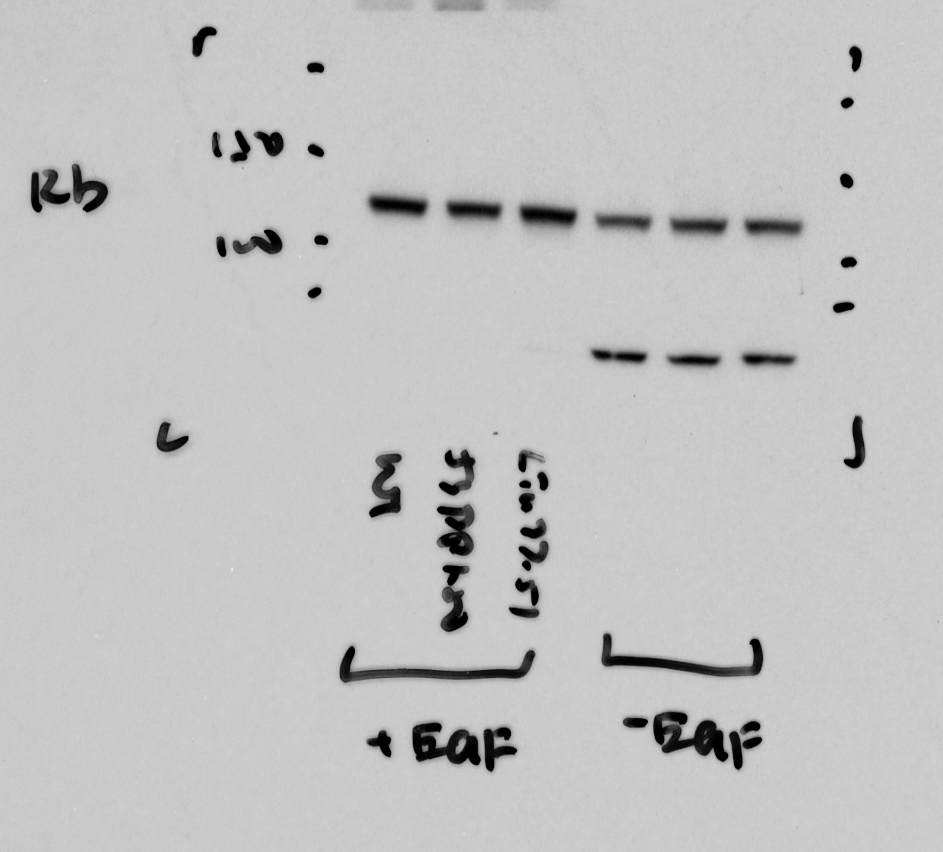

Supplement: Source data 6. [file elife-68466-data6.zip › Source data 6 - figure 6 and 7/Figure 7/1006200001_RB_7B.tif]

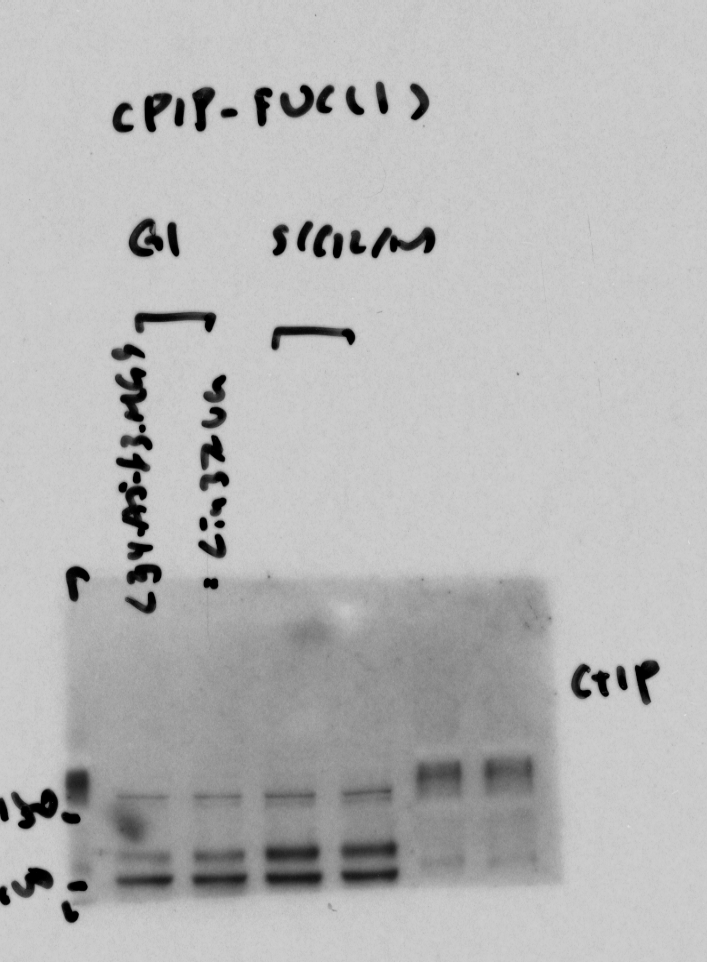

Supplement: Source data 6. [file elife-68466-data6.zip › Source data 6 - figure 6 and 7/Figure 7/100820_CTIP_Fig 7C.tif]

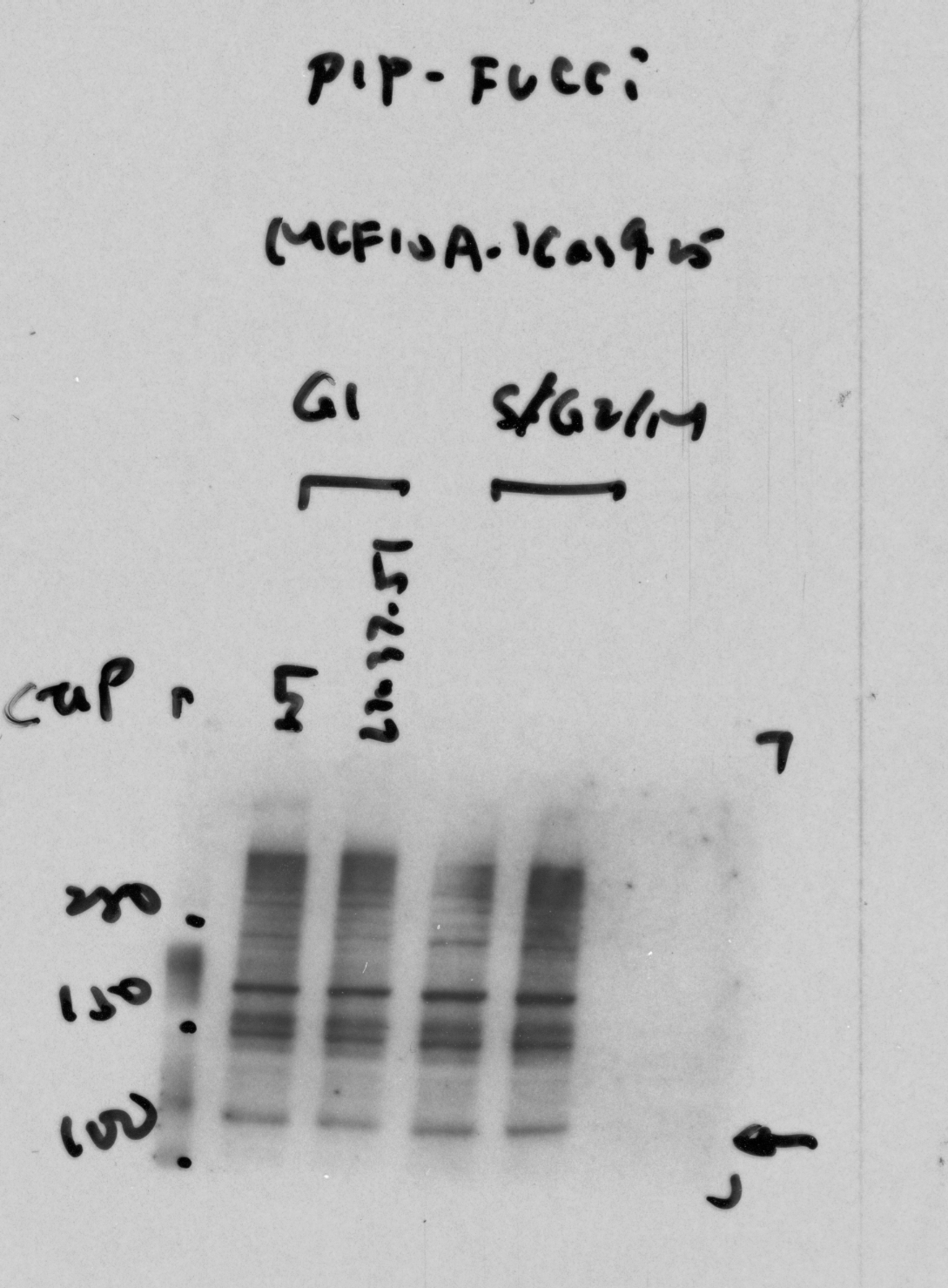

Supplement: Source data 6. [file elife-68466-data6.zip › Source data 6 - figure 6 and 7/Figure 7/101520_CtIP_Fig 7D.tif]

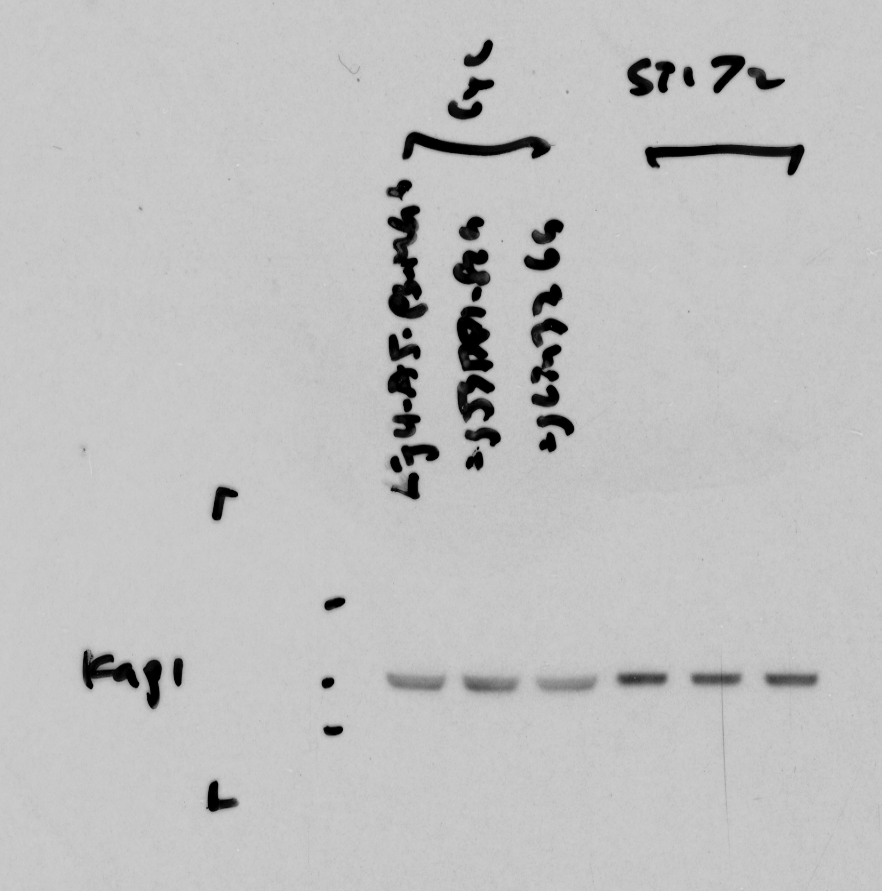

Supplement: Source data 6. [file elife-68466-data6.zip › Source data 6 - figure 6 and 7/Figure 7/0911200003_KAP1_Fig 7A.tif]

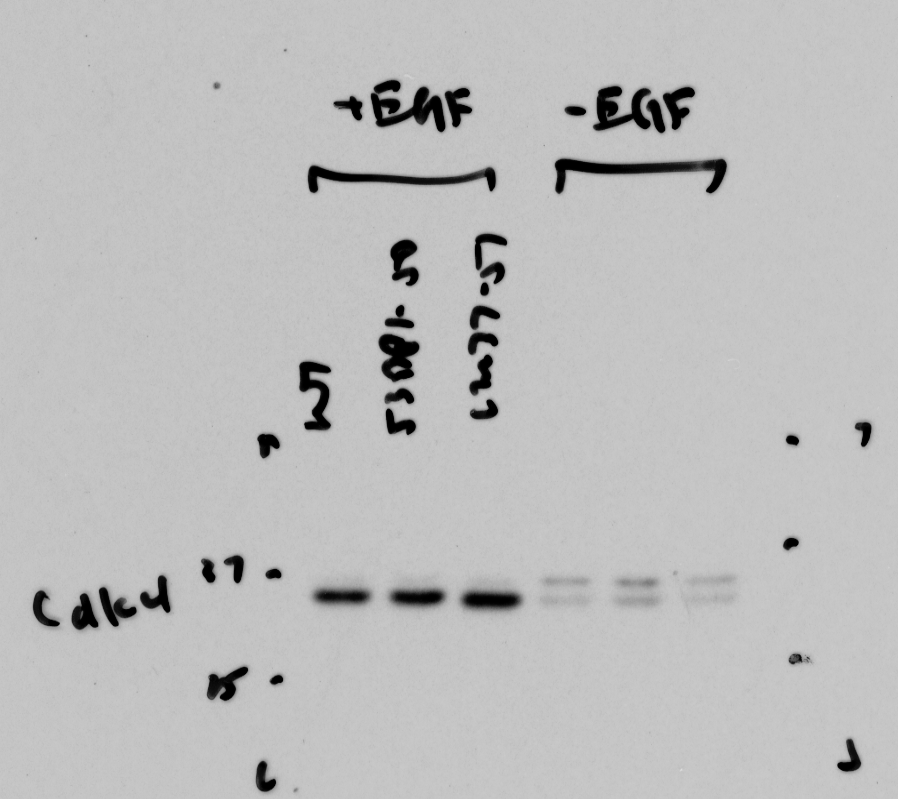

Supplement: Source data 6. [file elife-68466-data6.zip › Source data 6 - figure 6 and 7/Figure 7/1007200002_CDK4_Fig 7B.tif]

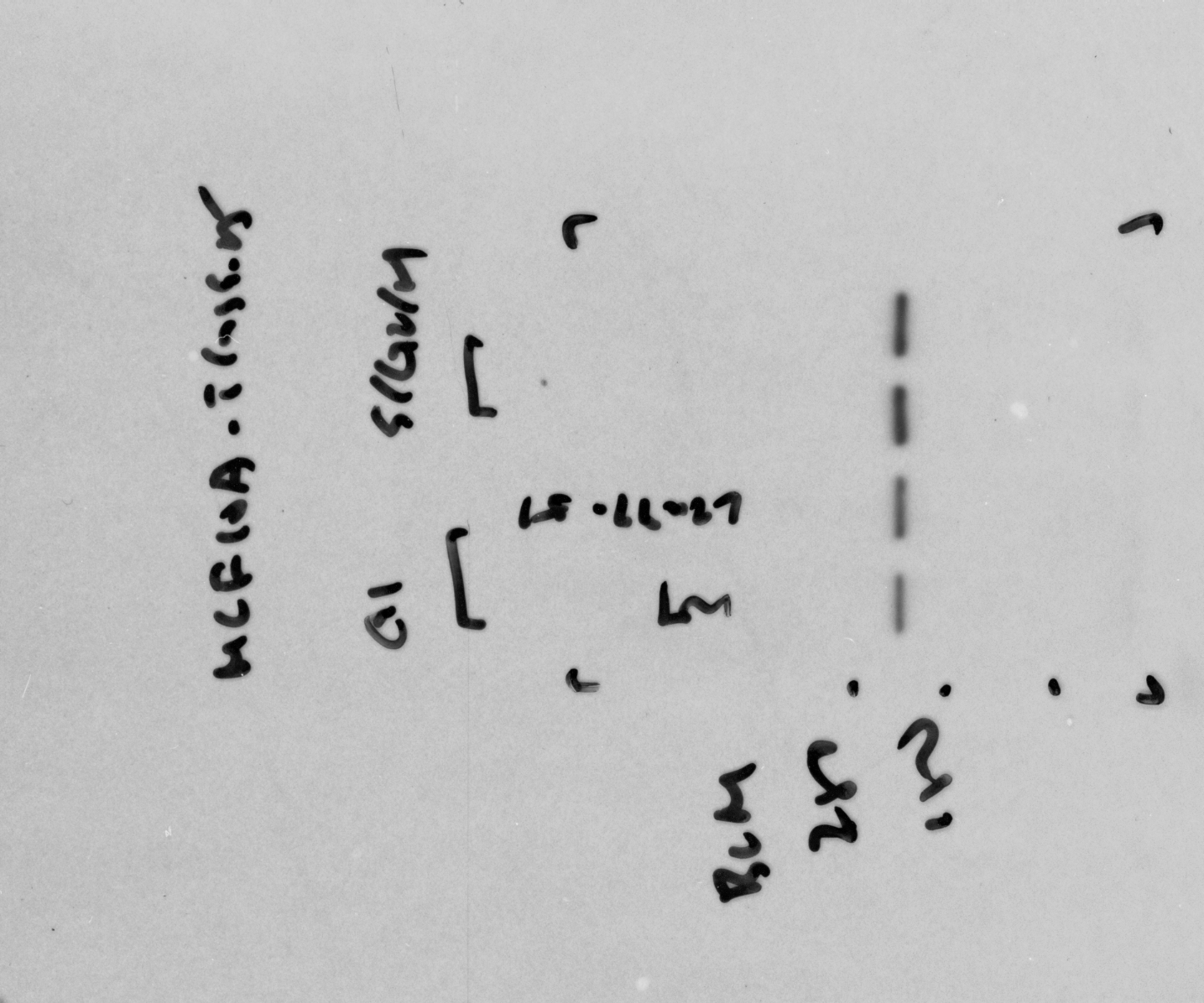

Supplement: Source data 6. [file elife-68466-data6.zip › Source data 6 - figure 6 and 7/Figure 7/101420_BLM_Fig 7D.tif]

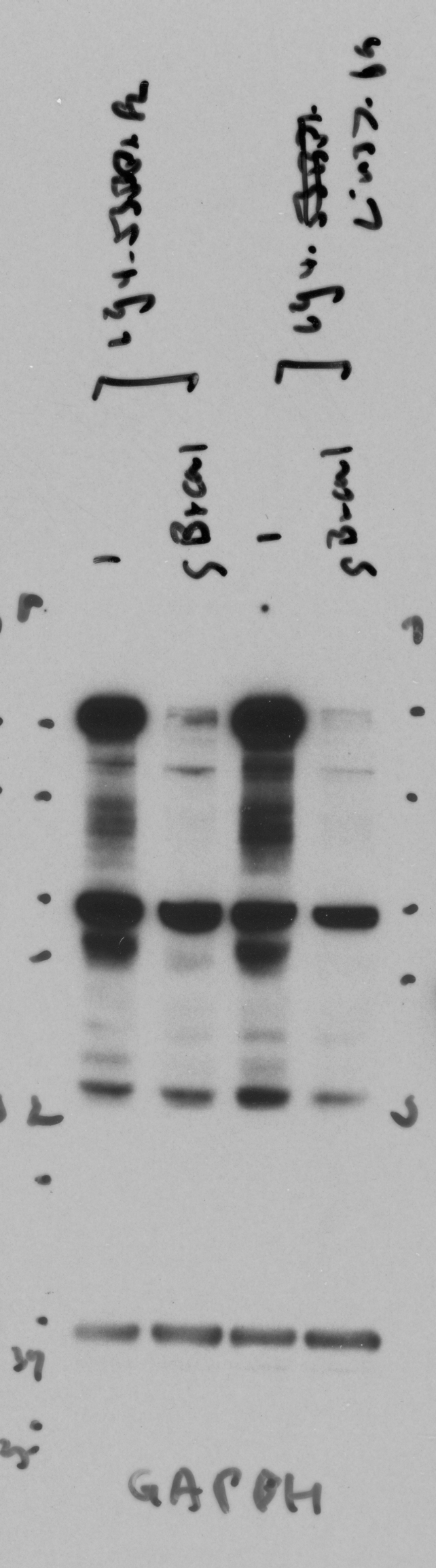

Supplement: Source data 6. [file elife-68466-data6.zip › Source data 6 - figure 6 and 7/Figure 6/042420_GAPDH_Fig 6A.tif]

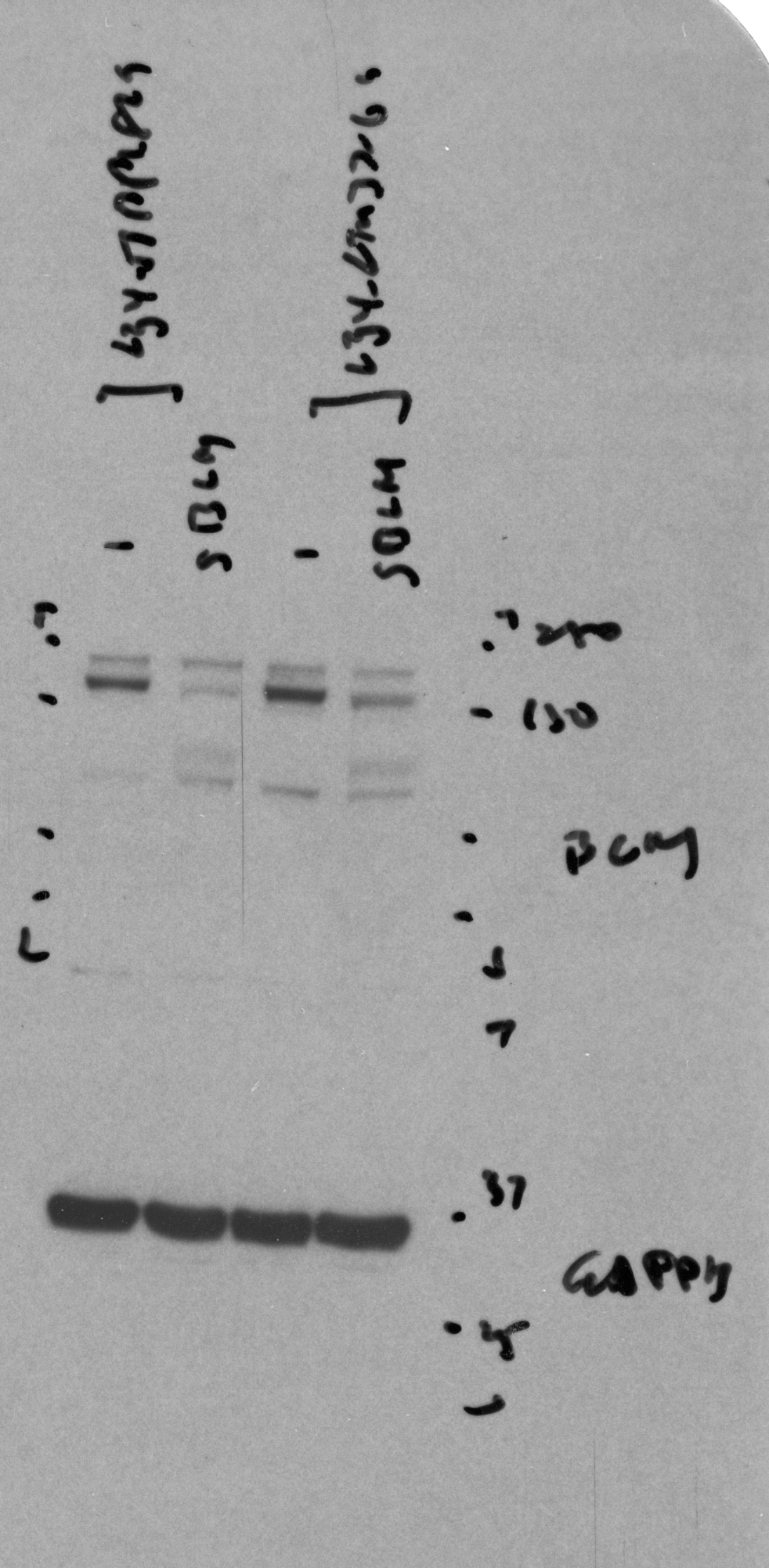

Supplement: Source data 6. [file elife-68466-data6.zip › Source data 6 - figure 6 and 7/Figure 6/050820_BLM-GAPDH_GAPDH_Fig 6A.tif]

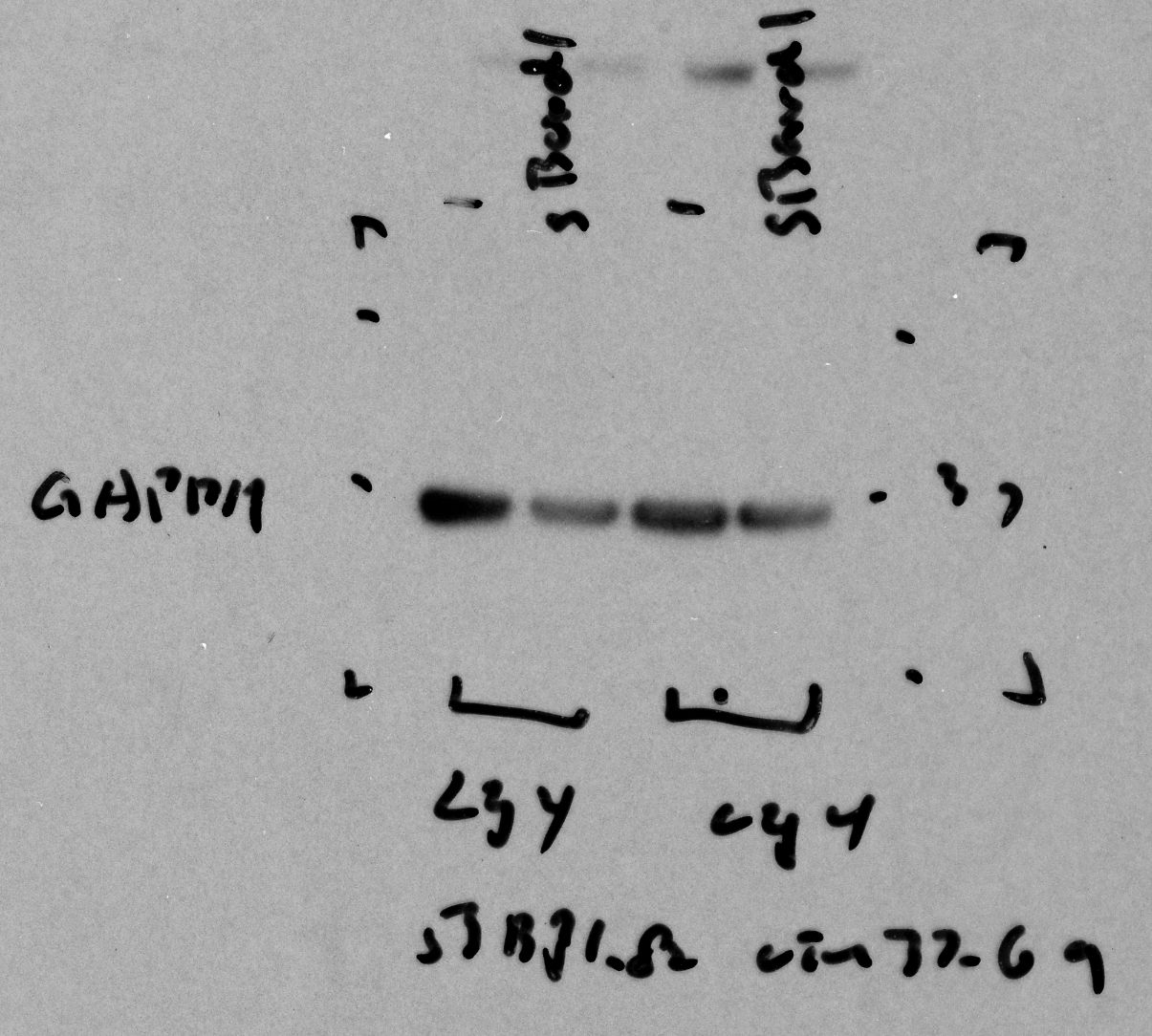

Supplement: Source data 6. [file elife-68466-data6.zip › Source data 6 - figure 6 and 7/Figure 6/0519200008_GAPDH_Fig 6A.tif]

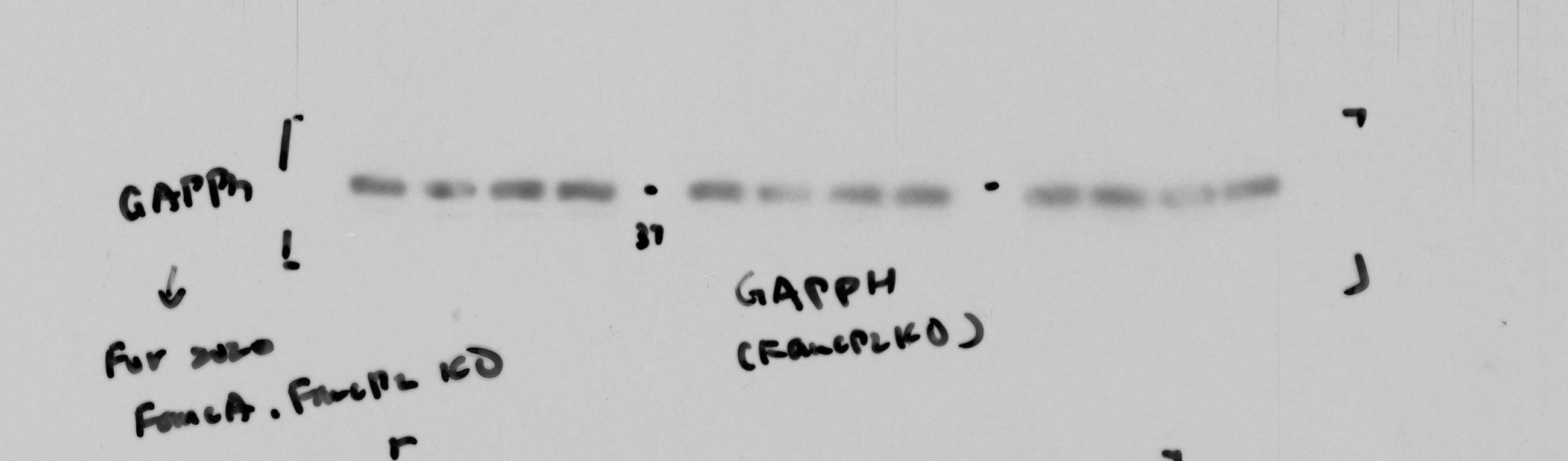

Supplement: Source data 6. [file elife-68466-data6.zip › Source data 6 - figure 6 and 7/Figure 6/08042020_GAPDH_Fig 6A.tif]

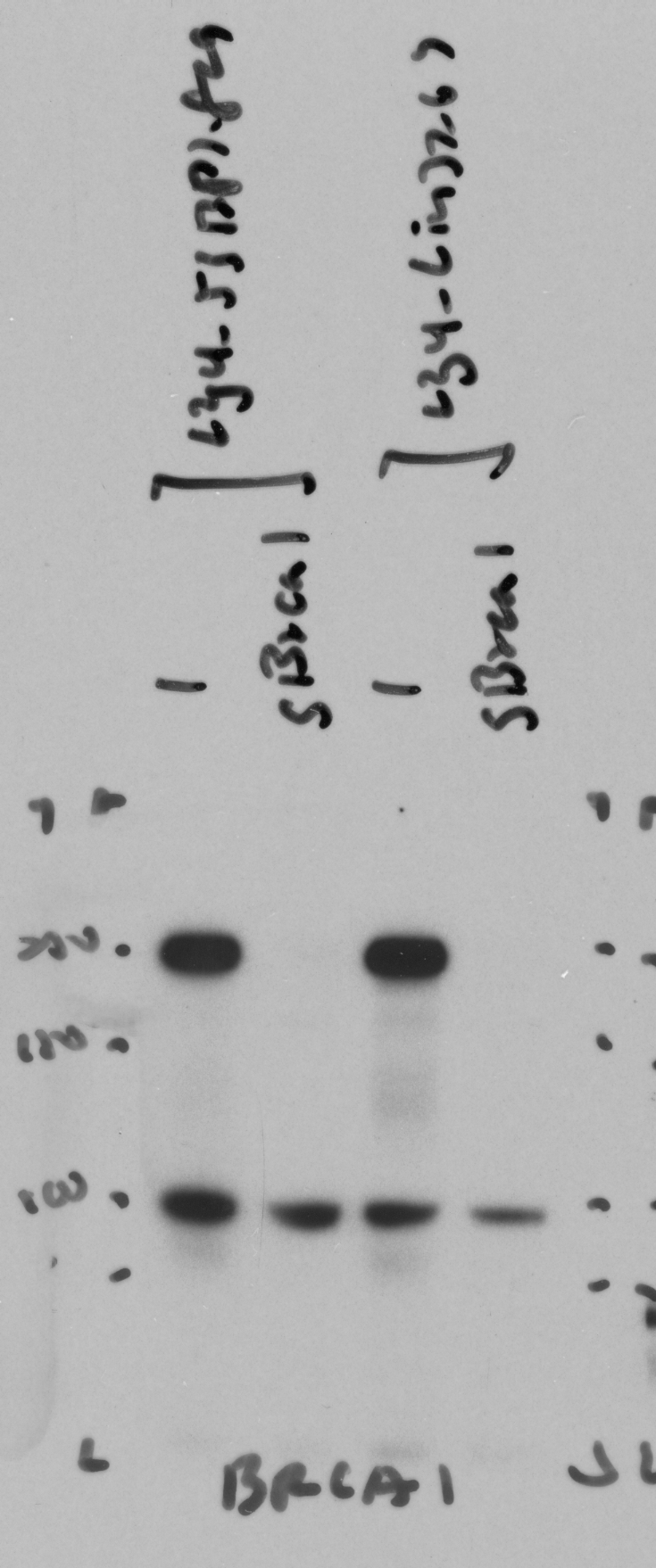

Supplement: Source data 6. [file elife-68466-data6.zip › Source data 6 - figure 6 and 7/Figure 6/042420_BRCA1_Fig 6A.tif]

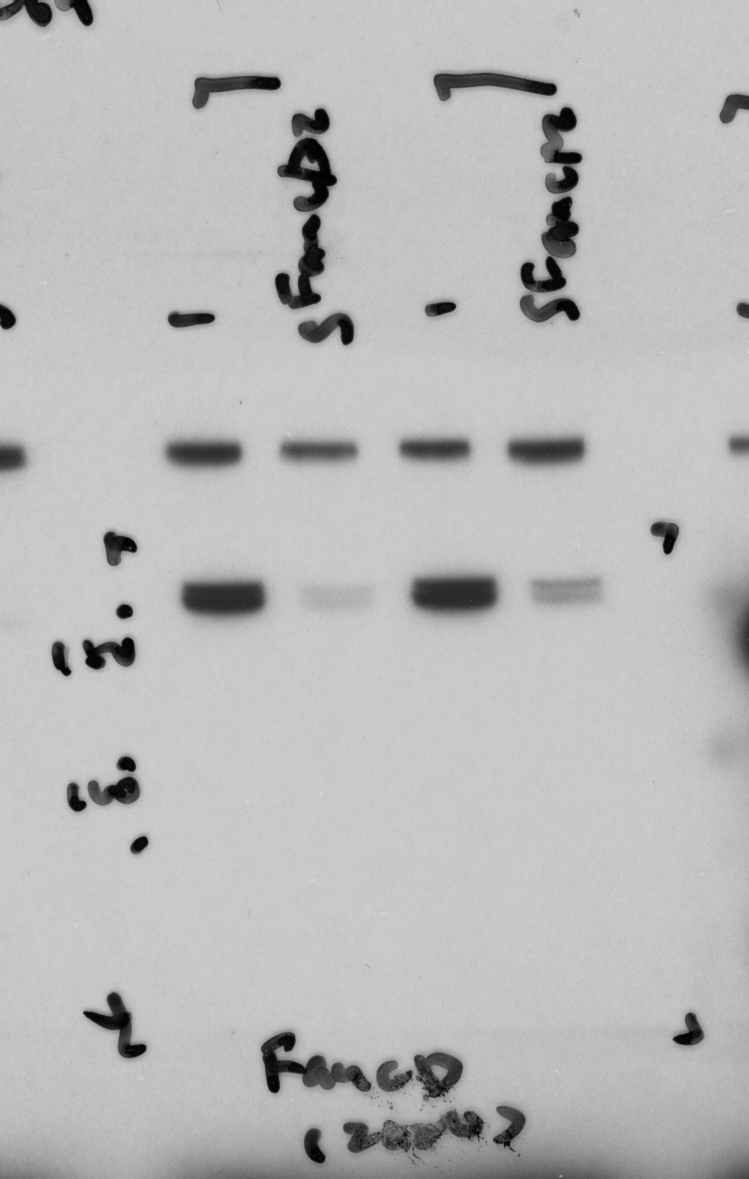

Supplement: Source data 6. [file elife-68466-data6.zip › Source data 6 - figure 6 and 7/Figure 6/08042020_FANCd2_Fig 6A.tif]

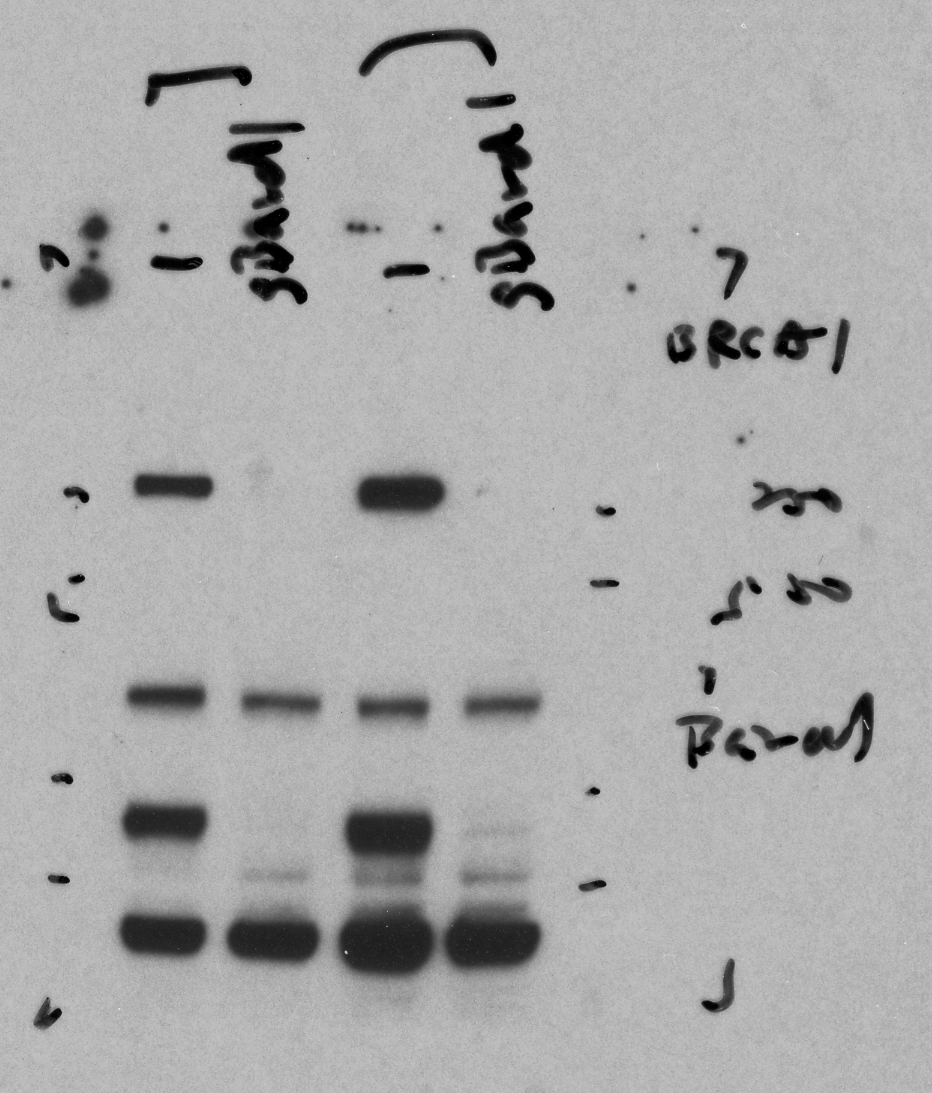

Supplement: Source data 6. [file elife-68466-data6.zip › Source data 6 - figure 6 and 7/Figure 6/0519200007_Bard1_Fig 6A.tif]
